# Benchmarking progress in non-communicable diseases: a global analysis of cause-specific mortality from 2001 to 2019

**DOI:** 10.1016/S0140-6736(25)01388-1

**Published:** 2025-09-10

**Authors:** NCD Countdown 2030 Collaborators, Data, methodology, analysis, presentation, and writing, James E Bennett, Olivia N O’Driscoll, Gretchen A Stevens, Nestor Aldea-Ramos, Michel Guillot, Majid Ezzati, Clinical, epidemiological, and health system interpretation, Freddie Bray, Farshad Farzadfar, Jürgen Rehm, Vikram Patel, Gill Livingston, Pablo Perel, Shekhar Saxena, Margaret E Kruk, Ole F Norheim, Rachel Nugent, Jean Claude Mbanya, Jonathan Pearson-Stuttard, Amirhossein Takian, Translation and dissemination, Leanne M Riley, Robert Beaglehole, Katie Dain

**Affiliations:** Imperial College London, London, UK; Imperial College London, London, UK; WHO, Geneva, Switzerland; French Institute for Demographic Studies, Aubervilliers, France; University of Pennsylvania, Philadelphia, PA, USA; French Institute for Demographic Studies, Aubervilliers, France; Imperial College London, London, UK; University of Ghana, Accra, Ghana; International Agency for Research on Cancer, Lyon, France; WHO, Geneva, Switzerland; Centre for Addiction and Mental Health, Toronto, ON, Canada; University of Toronto, Toronto, ON, Canada; Harvard Medical School, Boston, MA, USA; University College London, London, UK; London School of Hygiene & Tropical Medicine, London, UK; Harvard T H Chan School of Public Health, Boston, MA, USA; Washington University in St Louis, MO, USA; Harvard T H Chan School of Public Health, Boston, USA; University of Washington, Seattle, WA, USA; University of Yaoundé 1, Yaoundé, Cameroon; Lane Clark & Peacock, London, UK; Tehran University of Medical Sciences, Tehran, Iran; WHO, Geneva, Switzerland; University of Auckland, Auckland, New Zealand; NCD Alliance, Geneva, Switzerland

## Abstract

**Background:**

Non-communicable diseases (NCDs) have received substantial policy attention globally and in most countries. Our aim was to quantify how much NCD mortality changed from 2010 to 2019 in different countries, especially compared with the preceding decade and with the best-performing country in each region, and the specific NCD causes of death that contributed to change.

**Methods:**

We used data on NCD mortality by sex, age group, and underlying cause of death for 185 countries and territories from the 2021 WHO Global Health Estimates. Our primary outcome was the probability of dying from an NCD between birth and age 80 years in the absence of competing causes of death, and was calculated using age-specific death rates from NCDs and lifetable methods. We calculated change in the probability of death as the difference between values in the final and first year of each period (2001–10 and 2010–19). For 51 countries with high-quality mortality data and 12 countries with large populations within their region, we used the Horiuchi method of decomposition to calculate how much specific causes of death and 5-year age groups contributed towards: (1) increases or decreases in NCD mortality from 2010 to 2019; (2) improvements or deteriorations compared with the preceding decade (2001–10); and (3) differences from the country that had the largest reduction in each region.

**Findings:**

From 2010 to 2019, the probability of dying from an NCD between birth and age 80 years decreased in 152 (82%) of 185 countries for females and in 147 (79%) countries for males; it increased in the remaining 33 (18%) countries for females and 38 (21%) countries for males. The countries where NCD mortality declined for females accounted for 72% of the world female population in 2019, and those where NCD mortality declined for males accounted for 73% of the world male population. NCD mortality declined in all high-income western countries, with Denmark experiencing the largest decline for both sexes and the USA experiencing the smallest decline. Among the largest countries in other regions, NCD mortality declined for both sexes in China, Egypt, Nigeria, Russia, and Brazil, and increased for both sexes in India and Papua New Guinea. On average, females in countries in the central Asia, Middle East and north Africa region had the greatest reduction in NCD mortality followed by those in central and eastern Europe. For males, the largest reduction was among countries in central and eastern Europe, followed by those in central Asia, Middle East and north Africa. The smallest declines were those in the Pacific Island nations. Circulatory diseases were the greatest contributors to declines in NCD mortality from 2010 to 2019 in most countries, with some cancers (eg, stomach and colorectal cancers for both sexes, cervical and breast cancers for females, and lung and prostate cancers for males) also contributing towards lower NCD mortality in 2019 than in 2010 in many countries. Neuropsychiatric conditions and pancreatic and liver cancers contributed towards higher NCD mortality from 2010 to 2019 in most countries. In some countries, NCD mortality in working and older (≥65 years) ages changed in the same direction leading to large overall declines or increases; in others, it changed in opposite directions, diminishing the magnitude of the overall change. In 75 (41%) of 185 countries for females and in 73 (39%) countries for males, the change in NCD mortality from 2010 to 2019 was an improvement (ie, larger decline, smaller increase, or reversal of an increase) compared with the change from 2001 to 2010. These countries accounted for 29% and 63% of the world female and male population, respectively, and included both sexes in Russia and Egypt, and males in China, India, and Brazil. Decadal changes saw a deterioration (ie, smaller decline, larger increase, or reversal of a decline) in the remaining 110 (59%) countries for females and 112 (61%) countries for males, including in both sexes in the USA, Nigeria, and Papua New Guinea, and females in China, India, and Brazil. Change from 2010 to 2019 saw deterioration in direction or size compared with the preceding decade for both sexes in most high-income western countries, most countries in Latin America and the Caribbean, and in east and southeast Asia, and for females in south Asia. There was a decadal improvement in the direction or size of change for many countries in central and eastern Europe (eg, Russia) and central Asia, and in parts of the Middle East and north Africa. Improvements or deteriorations in the direction or size of change in NCD mortality between the two decades resulted from multiple NCD causes of death. Among causes of death, the decline in mortality from circulatory diseases was smaller from 2010 to 2019 than from 2001 to 2010 in most countries, except in countries in central and eastern Europe and some countries in central Asia, where these declines were larger from 2010 to 2019 than from 2001 to 2010. Change in lung cancer saw a decadal improvement in many countries, especially for males, and many other cancers saw a mix of improvement and deterioration.

**Interpretation:**

From 2010 to 2019, NCD mortality declined in four of every five countries in the world. These improvements were not as large as the preceding decade for most countries, driven by smaller declines in mortality from multiple NCDs.

**Funding:**

UK Medical Research Council, UK National Institute for Health and Care Research, and NCD Alliance.

## Introduction

Non-communicable diseases (NCDs) include cancers; cardiovascular diseases; diabetes; endocrine, blood, and immune disorders; non-infectious respiratory, digestive, and genitourinary diseases; neurological conditions; mental and substance use disorders; congenital anomalies; and sense organ, skin, musculoskeletal, and oral or dental conditions.^1,2^ In 2019, NCDs accounted for 42 million of the 57 million deaths globally; of these, 27 million were of those aged younger than 80 years.^1–3^

NCDs have received increasing policy attention globally and in most countries following the UN High-level Meetings on the prevention and control of NCDs. There have been numerous political pledges and national, regional, and global plans and recommendations on clinical and public health interventions to help reduce the health burden of NCDs. We do not know whether these efforts have accelerated the decline in NCDs (where they were already declining) or attenuated and reversed their increase (where they were rising). A comparative assessment of changes over time across countries is essential for evaluating the national impact of the political commitments and policy recommendations and their implementation, and can help draw lessons from countries that achieved greater mortality reductions.

In this Article, we benchmark changes in NCD mortality from 2010 until before the COVID-19 pandemic (2019) across countries and compare them with the previous decade (2001–10) to determine the extent of national progress in NCD control since the beginning of the millennium (panel 1). For countries with high-quality mortality data and for countries with the largest population within their region, we attribute change in overall NCD mortality, and its variations between time periods and countries, to specific causes of death and age groups.

## Methods

### Overview

We used data on mortality by underlying cause of death and lifetable methods to answer the following questions regarding changes in NCD mortality. (1a) How much did NCD mortality change from 2010 to 2019 in different countries? (1b) Which specific (NCD) causes of death and age groups drove the 2010–19 change? (2a) Which countries had more favourable changes (ie, a larger decline or smaller rise) versus less favourable changes from 2010 to 2019 compared with the preceding decade (2001–10)? (2b) Which NCD causes of death and age groups drove the decadal difference in changes? (3a) In each region, how did national changes in NCD mortality from 2010 to 2019 compare with the regional benchmark (ie, the country with the largest reduction)? (3b) Which causes of death and age groups were responsible for each country’s performance gap compared with the regional benchmark? We restricted the analysis up to and including 2019 because the COVID-19 pandemic, and the responses to it, affected NCD mortality in 2020 and 2021 for reasons stated in the appendix (p 7).

### Primary outcome

We used the probability of dying from an NCD between birth and age 80 years in the absence of competing causes of death to measure NCD mortality; this is referred to as unconditional probability of death. The probability of death depends only on age-specific death rates and is independent of the age structure of the population. Therefore, it is not affected by ageing of populations, except for the relatively small changes in age structure within 5-year age bands. The probability is calculated in the absence of competing causes of death so that it is only a function of age-specific mortality rates from the causes of interest, namely NCDs, and hence its calculation is not affected by differences across countries in mortality from non-NCD causes, namely infections or injuries.^4^ These properties make it an ideal measure for analysis of NCD mortality across global populations. Globally, the probability of dying from an NCD before age 80 years was 38% for females and 51% for males.

For reasons stated in the appendix (pp 3–6), the age range of birth to age 80 years is broader than the range used in the Sustainable Development Goal (SDG) target 3.4 (between age 30 and 70 years), and we consider all NCDs, whereas the SDG target 3.4 is restricted to cancers, cardiovascular diseases, chronic respiratory diseases, and diabetes.

### Data sources

We used data on deaths from NCDs by sex and age group for 185 countries and territories (referred to as countries hereafter) from the 2021 WHO Global Health Estimates as detailed in the appendix (pp 3–6).^3^ We divided these countries into eight reporting regions (appendix pp 9–10) using the regional assignment of the NCD Risk Factor Collaboration (NCD-RisC), which is based on their geography and epidemiology as relevant for NCDs.^5,6^ WHO also uses the completeness of death registration, the quality of the cause of death information, and the timeliness and frequency of data provided to WHO to classify countries’ death registration data quality as high, medium, low, or very low (appendix pp 9–10).

We report changes over time in the probability of dying from all NCDs for all 185 countries. We also attribute change in the probability of dying from any NCD to specific NCD causes of death for two groups of countries. The first group consisted of 51 countries with high-quality data and a 2019 population of more than 2 million. We used countries with high-quality data because estimates of specific causes of death require reliable medical certification of deaths. Of these 51 countries, 47 are classified as having high-quality data, and four (China, Russia, Taiwan, and Ukraine) have other data quality classifications (appendix pp 9–10). We included these four countries in the high-quality group because their mortality estimates also incorporate information from the Global Burden of Diseases, Injuries, and Risk Factors Study,^2,7^ which are based on more data than reported to WHO, resulting in estimates that reflect a higher data quality than their classification (appendix pp 9–10). We excluded countries with high-quality data but with a population of less than 2 million from the cause decomposition analysis because the number of deaths in each age group and from each cause is zero or small and can result in highly variable death rates and probabilities of death from year to year. The second group included 12 countries either with medium-quality data that were among the five largest countries by 2019 population size in their region, or were the largest country in each region irrespective of data quality. This process led to selection of 63 countries for cause-specific analysis, representing 69% of the global population in 2019.

### Statistical analysis

We used age-specific death rates and lifetables to calculate the primary outcome—ie, unconditional probability of dying from an NCD between birth and age 80 years—by country and sex (appendix pp 3–6).^4^ We calculated change in the unconditional probabilities of death from 2001 to 2010 and from 2010 to 2019 as the difference between the corresponding values in the final and first year of each period so that there is no assumption about linearity of change.

We quantified the contributions of specific NCD causes of death and age groups to changes (question 1b), as well as to the difference in changes over time (question 2b) and across countries (question 3b), in the unconditional probability of dying from all NCDs between birth and age 80 years. For these analyses, we used the 16 leading NCD causes or aetiologically related groups of causes of death based on the total number of deaths before age 80 years from 2010 to 2019 in the aforementioned 51 countries (appendix pp 11–12). We also included four residual categories: all other circulatory diseases, all other malignant neoplasms (cancers), all other neuropsychiatric conditions and all other NCDs (appendix pp 11–12). This classification created 20 mutually exclusive and collectively exhaustive groups of NCD causes of death.

We applied the Horiuchi method of decomposition for the analyses related to questions 1b, 2b, and 3b (appendix pp 3–6).^8^ All analyses were done separately by sex in R (version 4.4.2).

### Role of the funding source

NCD Alliance provided partial funding for this work and provided input on the implications of the research for global policy discussions, especially at the UN High-level Meeting; however, the corresponding author had the final responsibility for the interpretation of results. All other funders of the study had no role in study design, data collection, data analysis, data interpretation, or writing of the report.

## Results

### NCD mortality from 2010 to 2019

**Figure 1:**
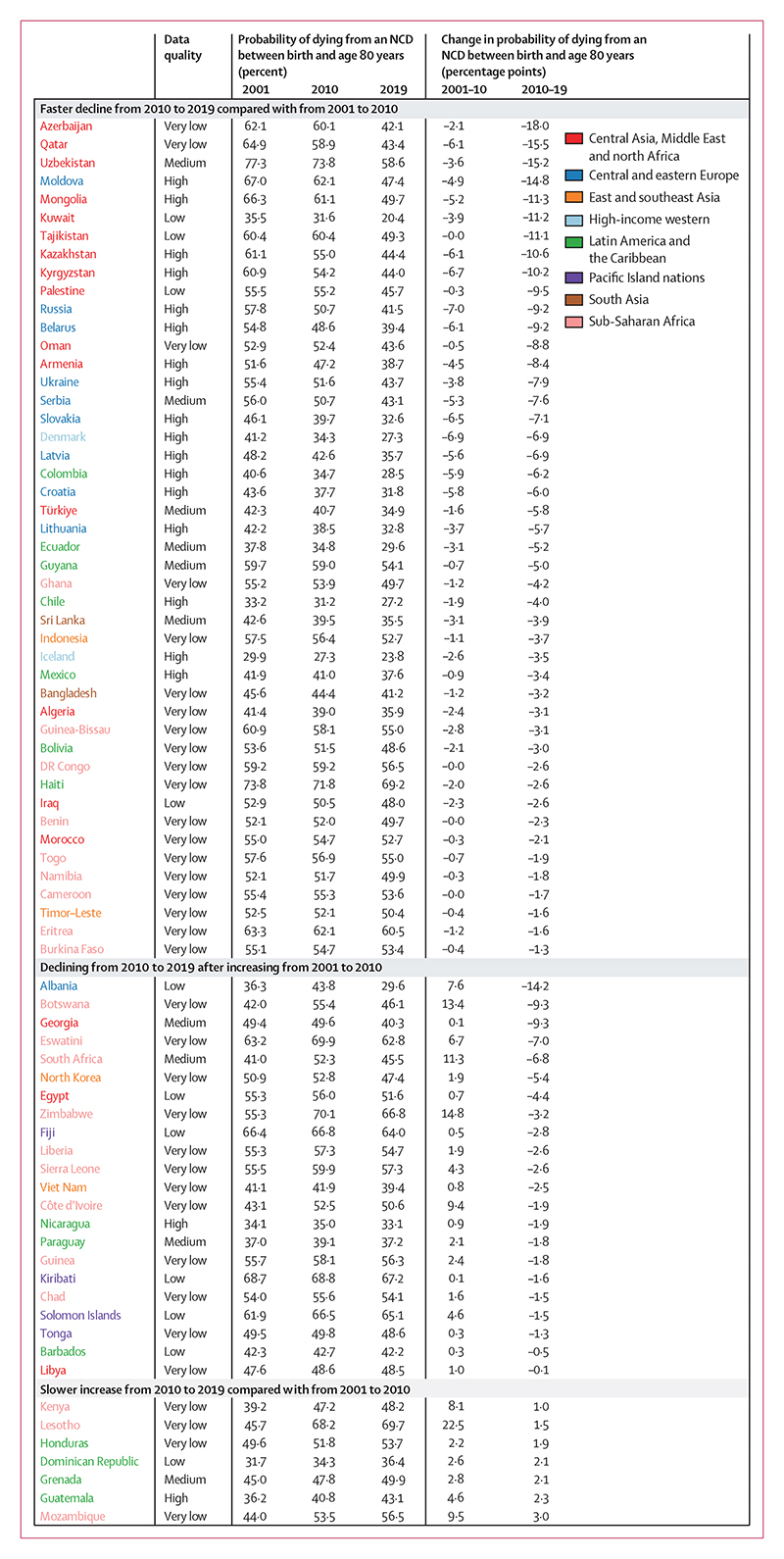
Countries showing improvements in the direction or size of change from 2010 to 2019 compared with from 2001 to 2010 for females Estimates are shown for all countries and territories in eight reporting regions. Countries are divided into three categories of changes observed across two timeframes (2001–10 and 2010–19). Within each of the three categories, countries are ordered by percentage point change from 2010 to 2019 (ie, the first country listed in each category had the largest decrease or smallest increase from 2010 to 2019 within its category). For the estimated probabilities and change in probabilities with uncertainty intervals see the appendix (pp 44–53). For results based on cancers, cardiovascular diseases, chronic respiratory diseases, and diabetes in ages 30–70 years see the appendix (pp 13–14). NCD=non-communicable disease.

**Figure 2:**
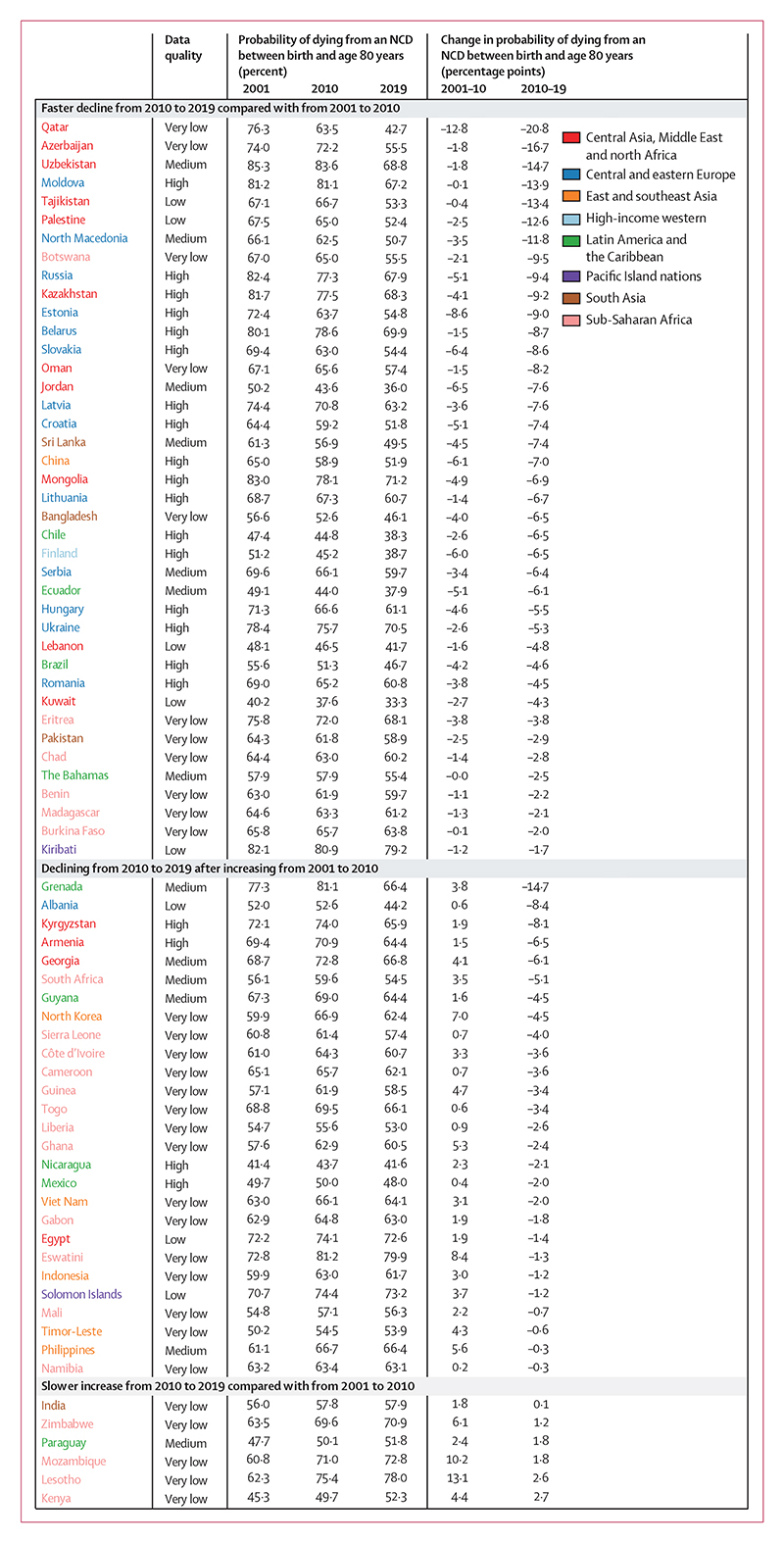
Countries showing improvements in the direction or size of change from 2010 to 2019 compared with from 2001 to 2010 for males Estimates are shown for all countries and territories in eight reporting regions. Countries are divided into three categories of changes observed across two timeframes (2001–10 and 2010–19). Within each of the three categories, countries are ordered by percentage point change from 2010 to 2019 (ie, the first country listed in each category had the largest decrease or smallest increase from 2010 to 2019 within its category). For the estimated probabilities and change in probabilities with uncertainty intervals see the appendix (pp 44–53). For results based on cancers, cardiovascular diseases, chronic respiratory diseases, and diabetes in ages 30–70 years see the appendix (pp 13–14). NCD=non-communicable disease.

**Figure 3:**
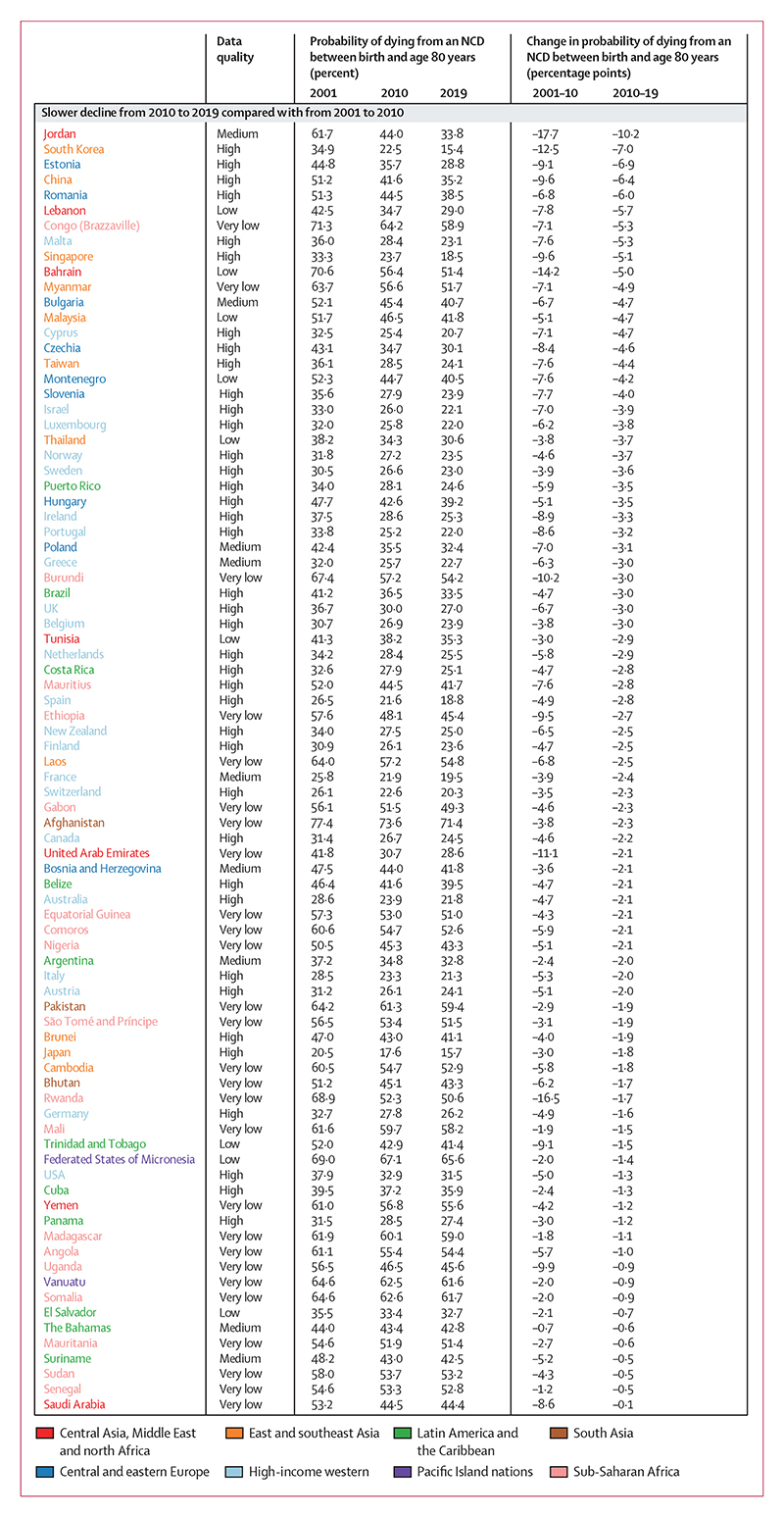

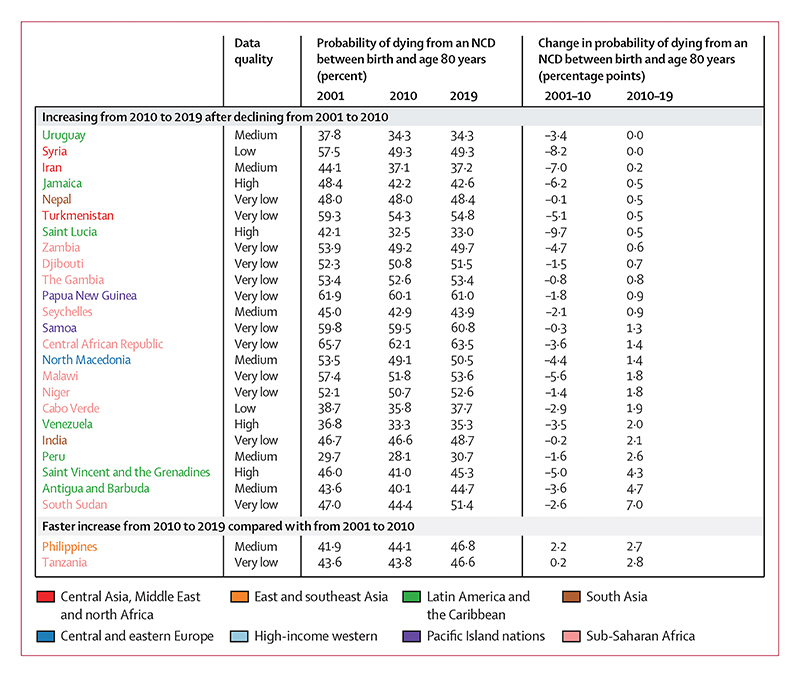
Countries showing deteriorations in the direction or size of change from 2010 to 2019 compared with from 2001 to 2010 for females Estimates are shown for all countries and territories in eight reporting regions. Countries are divided into three categories of changes observed across two timeframes (2001–10 and 2010–19). Within each of the three categories, countries are ordered by percentage point change from 2010 to 2019 (ie, the first country listed in each category had the largest decrease or smallest increase from 2010 to 2019 within its category). For the estimated probabilities and change in probabilities with uncertainty intervals see the appendix (pp 44–53). For results based on cancers, cardiovascular diseases, chronic respiratory diseases, and diabetes in ages 30–70 years see the appendix (pp 13–14). NCD=non-communicable disease.

**Figure 4:**
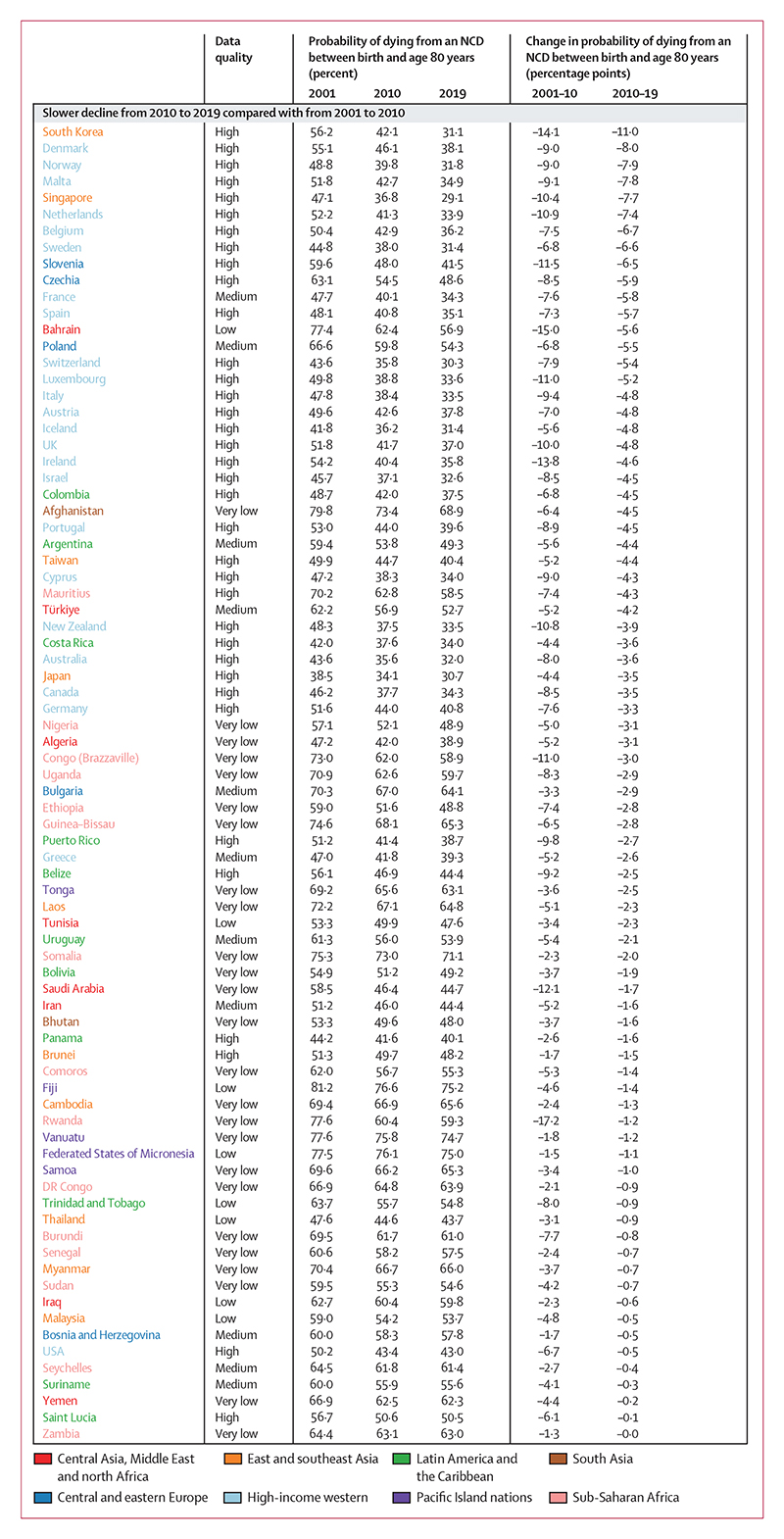

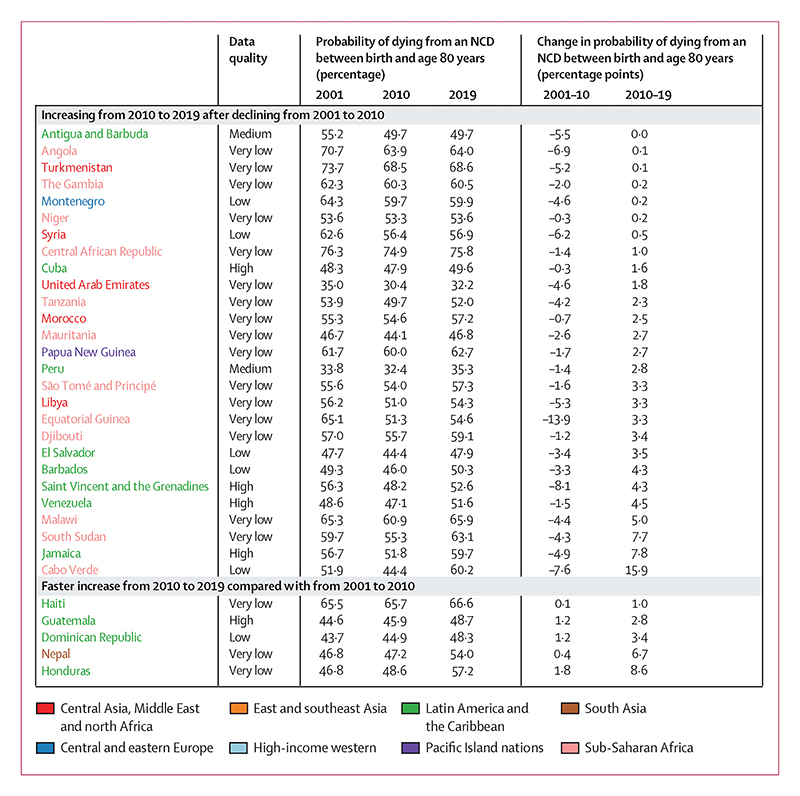
Countries showing deteriorations in the direction or size of change from 2010 to 2019 compared with from 2001 to 2010 for males Estimates are shown for all countries and territories in eight reporting regions. Countries are divided into three categories of changes observed across two timeframes (2001–10 and 2010–19). Within each of the three categories, countries are ordered by percentage point change from 2010 to 2019 (ie, the first country listed in each category had the largest decrease or smallest increase from 2010 to 2019 within its category). For the estimated probabilities and change in probabilities with uncertainty intervals see the appendix (pp 44–53). For results based on cancers, cardiovascular diseases, chronic respiratory diseases, and diabetes in ages 30–70 years see the appendix (pp 13–14). NCD=non-communicable disease.

**Figure 5:**
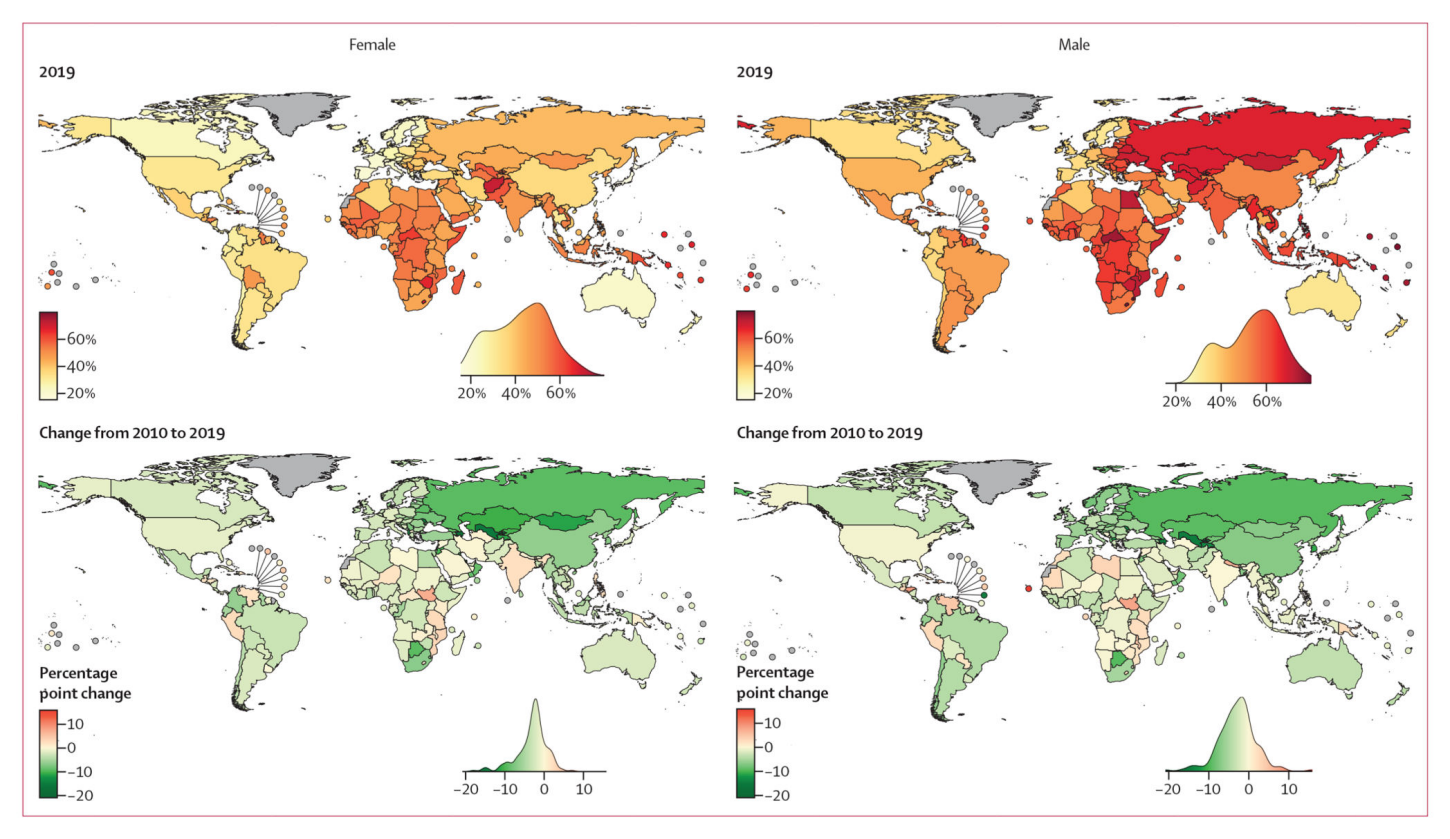
Probability of dying from an NCD between birth and age 80 years in 2019 and change in probability from 2010 to 2019 For change from 2010 to 2019, green indicates a decline in NCD mortality and red indicates an increase. The density plot alongside each map shows the smoothed distribution of estimates across countries. Countries and territories with no mortality estimates are shown in grey. For the estimated probabilities and change in probabilities with uncertainty intervals see the appendix (pp 44–53). For results based on cancers, cardiovascular diseases, chronic respiratory diseases, and diabetes in ages 30–70 years see the appendix (pp 54–55). NCD=non-communicable disease.

**Figure 6:**
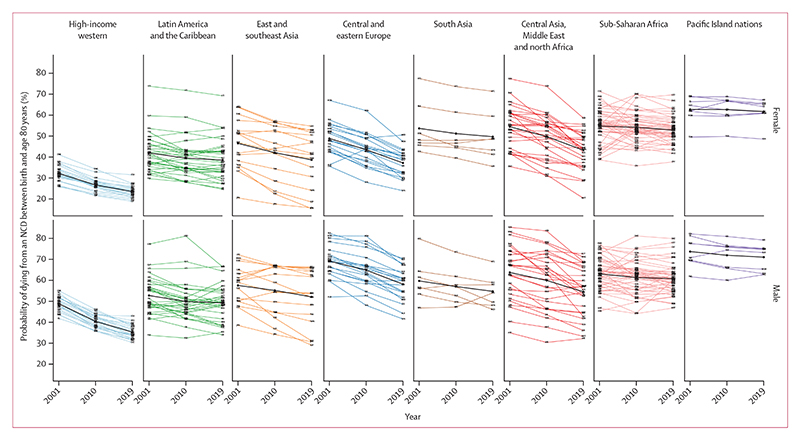
Change in NCD mortality from 2001 to 2019 Each line connects the probability of dying between birth and age 80 years from an NCD for 2001, 2010, and 2019 for one country. For each country, the difference in level between consecutive pairs of years respectively represents change over the intervals from 2001 to 2010 and from 2010 to 2019. Data are shown for 185 countries and territories, divided into eight reporting regions. Lines are coloured by region and labelled with ISO3 codes for each country. The bold black line in each panel connects the mean levels (across countries in that panel, unweighted for population) in 2001, 2010, and 2019. Regions are ordered by increasing mean probability of dying of the countries in each region for females in 2001. For results based on cancers, cardiovascular diseases, chronic respiratory diseases, and diabetes in ages 30–70 years see the appendix (pp 56–57). NCD=non-communicable disease. High-income western countries: AUS=Australia, AUT=Austria, BEL=Belgium, CAN=Canada, CHE=Switzerland, CYP=Cyprus, DEU=Germany, DNK=Denmark, ESP=Spain, FIN=Finland, FRA=France, GBR=United Kingdom, GRC=Greece, IRL=Ireland, ISL=Iceland, ISR=Israel, ITA=Italy, LUX=Luxembourg, MLT=Malta, NLD=Netherlands, NOR=Norway, NZL=New Zealand, PRT=Portugal, SWE=Sweden, USA=United States of America. Latin America and the Caribbean: ARG=Argentina, ATG=Antigua and Barbuda, BHS=The Bahamas, BLZ=Belize, BOL=Bolivia, BRA=Brazil, BRB=Barbados, CHL=Chile, COL=Colombia, CRI=Costa Rica, CUB=Cuba, DOM=Dominican Republic, ECU=Ecuador, GRD=Grenada, GTM=Guatemala, GUY=Guyana, HND=Honduras, HTI=Haiti, JAM=Jamaica, LCA=Saint Lucia, MEX=Mexico, NIC=Nicaragua, PAN=Panama, PER=Peru, PRI=Puerto Rico, PRY=Paraguay, SLV=El Salvador, SUR=Suriname, TTO=Trinidad and Tobago, URY=Uruguay, VCT=Saint Vincent and the Grenadines, VEN=Venezuela. East and southeast Asia: BRN=Brunei, CHN=China, IDN=Indonesia, JPN=Japan, KHM=Cambodia, KOR=South Korea, LAO=Laos, MMR=Myanmar, MYS=Malaysia, PHL=Philippines, PRK=North Korea, SGP=Singapore, THA=Thailand, TLS=Timor-Leste, TWN=Taiwan, VNM=Viet Nam. Central and eastern Europe: ALB=Albania, BGR=Bulgaria, BIH=Bosnia and Herzegovina, BLR=Belarus, CZE=Czechia, EST=Estonia, HRV=Croatia, HUN=Hungary, LTU=Lithuania, LVA=Latvia, MDA=Moldova, MKD=North Macedonia, MNE=Montenegro, POL=Poland, ROU=Romania, RUS=Russia, SRB=Serbia, SVK=Slovakia, SVN=Slovenia, UKR=Ukraine. South Asia: AFG=Afghanistan, BGD=Bangladesh, BTN=Bhutan, IND=India, LKA=Sri Lanka, NPL=Nepal, PAK=Pakistan. Central Asia, Middle East and north Africa: ARE=United Arab Emirates, ARM=Armenia, AZE=Azerbaijan, BHR=Bahrain, DZA=Algeria, EGY=Egypt, GEO=Georgia, IRN=Iran, IRQ=Iraq, JOR=Jordan, KAZ=Kazakhstan, KGZ=Kyrgyzstan, KWT=Kuwait, LBN=Lebanon, LBY=Libya, MAR=Morocco, MNG=Mongolia, OMN=Oman, PSE=Palestine, QAT=Qatar, SAU=Saudi Arabia, SYR=Syria, TJK=Tajikistan, TKM=Turkmenistan, TUN=Tunisia, TUR=Türkiye, UZB=Uzbekistan, YEM=Yemen. Sub-Saharan Africa: AGO=Angola, BDI=Burundi, BEN=Benin, BFA=Burkina Faso, BWA=Botswana, CAF=Central African Republic, CIV=Côte d’Ivoire, CMR=Cameroon, COD=DR Congo, COG=Congo (Brazzaville), COM=Comoros, CPV=Cabo Verde, DJI=Djibouti, ERI=Eritrea, ETH=Ethiopia, GAB=Gabon, GHA=Ghana, GIN=Guinea, GMB=The Gambia, GNB=Guinea-Bissau, GNQ=Equatorial Guinea, KEN=Kenya, LBR=Liberia, LSO=Lesotho, MDG=Madagascar, MLI=Mali, MOZ=Mozambique, MRT=Mauritania, MUS=Mauritius, MWI=Malawi, NAM=Namibia, NER=Niger, NGA=Nigeria, RWA=Rwanda, SDN=Sudan, SEN=Senegal, SLE=Sierra Leone, SOM=Somalia, SSD=South Sudan, STP=São Tomé and Príncipe, SWZ=Eswatini, SYC=Seychelles, TCD=Chad, TGO=Togo, TZA=Tanzania, UGA=Uganda, ZAF=South Africa, ZMB=Zambia, ZWE=Zimbabwe. Pacific Island nations: FJI=Fiji, FSM=Federated States of Micronesia, KIR=Kiribati, PNG=Papua New Guinea, SLB=Solomon Islands, TON=Tonga, VUT=Vanuatu, WSM=Samoa.

The unconditional probability of dying from an NCD between birth and age 80 years declined in 152 (82%) of 185 countries for females and 147 (79%) countries for males from 2010 to 2019 (figures 1–6). The countries where NCD mortality declined for females accounted for 72% of the world female population in 2019, and the countries where NCD mortality declined for males accounted for 73% of the world male population. In 44 (29%) of the 152 countries for females and in 58 (39%) of the 147 countries for males, the 95% uncertainty interval (UI) of change excluded zero, which means that the observed decline was distinct from no change at the p=0·95 level.

Females in countries in the central Asia, Middle East and north Africa region on average had the greatest reduction (mean absolute decline of 6·6 percentage points from 2010 to 2019, with 25 [89%] of 28 countries in the region experiencing a decline), followed by those in central and eastern Europe. For males, the largest reduction was among countries in central and eastern Europe (mean absolute decline of 6·7 percentage points with 19 [95%] of 20 countries having a decline), followed by central Asia, Middle East and north Africa. The smallest declines were those in the Pacific Island nations, where the mean absolute reduction was less than 1·0 percentage point for both sexes, despite having some of the highest levels of NCD mortality globally in 2010.

In terms of variation in whether NCD mortality decreased versus increased within each region, all 25 countries classified as high-income western countries had small to moderate declines for both sexes (mean absolute decline of 3·1 percentage points for females and 5·1 percentage points for males from 2010 to 2019), making this the only region with a universal national decline (figure 6). Latin America and the Caribbean showed the most heterogeneity in the direction of change, with females in 21 (66%) of 32 countries and males in 19 (59%) of 32 countries recording declines.

Nationally, the size of change was below 2·0 percentage points in 68 countries for females and 58 countries for males; it was from 2·0 to less than 5·0 percentage points in 77 countries for females and 71 countries for males, and 5·0 percentage points or higher in 40 countries for females and 56 countries for males (figure 5). The greatest reductions in NCD mortality from 2010 to 2019 were in some countries in central Asia, Middle East and north Africa (figures 1, 2, 5). The three largest declines for females and males were in Azerbaijan (–18·0 percentage points [95% UI –24·8 to –11·3] lower probability of dying from an NCD in 2019 than in 2010 for females, and –16·7 percentage points [–22·9 to –10·5] for males), Qatar (–15·5 percentage points [–26·0 to –4·9] for females and –20·8 percentage points [–31·9 to –9·8] for males), and Uzbekistan (–15·2 percentage points [–18·9 to –11·3] for females and –14·7 percentage points [–18·1 to –11·5] for males). Data quality was rated as low for Qatar and Azerbaijan, which makes these results uncertain. Countries in central and eastern Europe also saw large declines, with Moldova showing the fourth largest decline for females (–14·8 percentage points [–19·0 to –10·6]) and the fifth largest decline for males (–13·9 percentage points [–17·3 to –10·6]).

Increases in NCD mortality over the same period were observed in 33 countries for females, of which 13 were in sub-Saharan Africa (27% of the 49 countries in the region) and 11 were in Latin America and the Caribbean (34% of the 32 countries in the region), and in 38 countries for males, of which 16 were in sub-Saharan Africa (33% of countries in the region) and 13 were in Latin America and the Caribbean (41% of countries in the region; figures 1–6). For females, the largest increases were in South Sudan, and Antigua and Barbuda. For males, the largest increases were in Cabo Verde, Honduras, and Jamaica. Data quality was rated as low or very low for these countries, except for Jamaica and Antigua and Barbuda, which makes these results uncertain (appendix pp 52–53). Countries with high-quality or medium-quality data and large increases in NCD mortality were Antigua and Barbuda, the Philippines, Peru, and Guatemala for females, and Jamaica, Venezuela, Guatemala, and Peru for males.

Among the largest countries in different regions, NCD mortality declined for both sexes in China (–6·4 percentage points [95% UI –12·0 to –0·9] for females and –7·0 percentage points [–13·1 to –1·1] for males), Egypt (–4·4 percentage points [–12·1 to 3·5] for females and –1·4 percentage points [–9·6 to 6·9] for males), Nigeria (–2·1 percentage points [–11·1 to 6·8] for females and –3·1 percentage points [–13·0 to 6·6] for males), Russia (–9·2 percentage points [–11·0 to –7·4] for females and –9·4 percentage points [–10·5 to –8·4] for males), Brazil (–3·0 percentage points [–6·1 to 0·1] for females and –4·6 percentage points [–7·2 to –2·0] for males) and the USA (–1·3 percentage points [–4·3 to 1·6] for females and –0·5 percentage points [–2·6 to 1·7] for males). It increased for both sexes in Papua New Guinea (0·9 percentage points [–10·2 to 12·0] for females and 2·7 percentage points [–9·0 to 13·9] for males) and India (2·1 percentage points [–4·3 to 8·5] for females and 0·1 percentage points [–5·8 to 5·9] for males).

For males, countries with higher 2010 NCD mortality had a weak tendency to have greater absolute declines in the probability of death from NCDs, with a correlation coefficient of –0·19 (appendix pp 17–18). The correlation coefficient between change in mortality and its 2010 level was only –0·09 for females, indicating that how much mortality changed was unrelated to its starting level; it also indicates that the variation in NCD mortality across countries did not shrink and there was no global convergence in NCD mortality. Regionally, there was convergence in NCD mortality, as measured by a shrinking within-region standard deviation, in central Asia, Middle East and north Africa, and to a lesser extent in central and eastern Europe, and sub-Saharan Africa. There was divergence in mortality in east and southeast Asia, where some countries with lower baseline mortality in 2010 (eg, Singapore and South Korea) had larger declines, increasing regional disparities. There was little or inconsistent change in standard deviation of NCD mortality across countries in other regions.

In 2019, the populations of Pacific Island nations and countries in sub-Saharan Africa had some of the highest probabilities of dying from an NCD before age 80 years, and those of the high-income western countries and high-income countries and emerging economies in East Asia had some of the lowest. Nationally, the probability of dying from an NCD before age 80 years ranged from less than 16% (less than one in six) for females in South Korea and Japan to more than 65% for females in Afghanistan, Lesotho, Haiti, Kiribati, Zimbabwe, and Micronesia, leading to a more than 50 percentage-point global gap in NCD mortality (figure 5). Similarly, males in Singapore, Switzerland, and Japan, with approximately 30% probability of dying from an NCD before age 80 years, had almost a 50 percentage-point advantage over those in Eswatini, Kiribati, and Lesotho, with probabilities of almost 80%. For males, some countries in eastern Europe, such as Ukraine, Belarus, and Russia, also had high NCD mortality in 2019, with a more than 60% probability of dying from an NCD before age 80 years, despite having had large declines from 2010 to 2019.

Change in mortality showed greater variability in males than in females from 2010 to 2019. For instance, the standard deviation of the change in the probability of dying from an NCD was 4·7 percentage points for males and 3·9 percentage points for females. In 94 (51%) of 185 countries, NCD mortality declined more for males than for females from 2010 to 2019. Reductions were larger among males than females in most high-income western countries and countries in central and eastern Europe (appendix pp 19–20). In most countries in central Asia, Middle East and north Africa, NCD mortality declined more for females than for males. The sex patterns of reduction were mixed in other regions. Despite these variations in mortality changes in relation to sex, the probability of dying from an NCD before age 80 years was smaller for females than for males in 178 (96%) of 185 countries in 2019.

**Figure 7:**
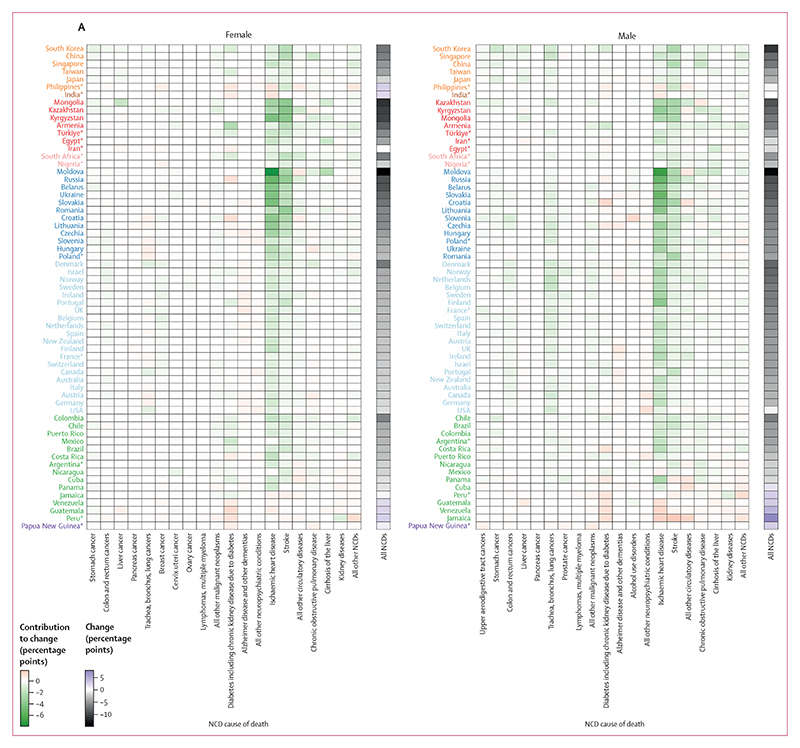

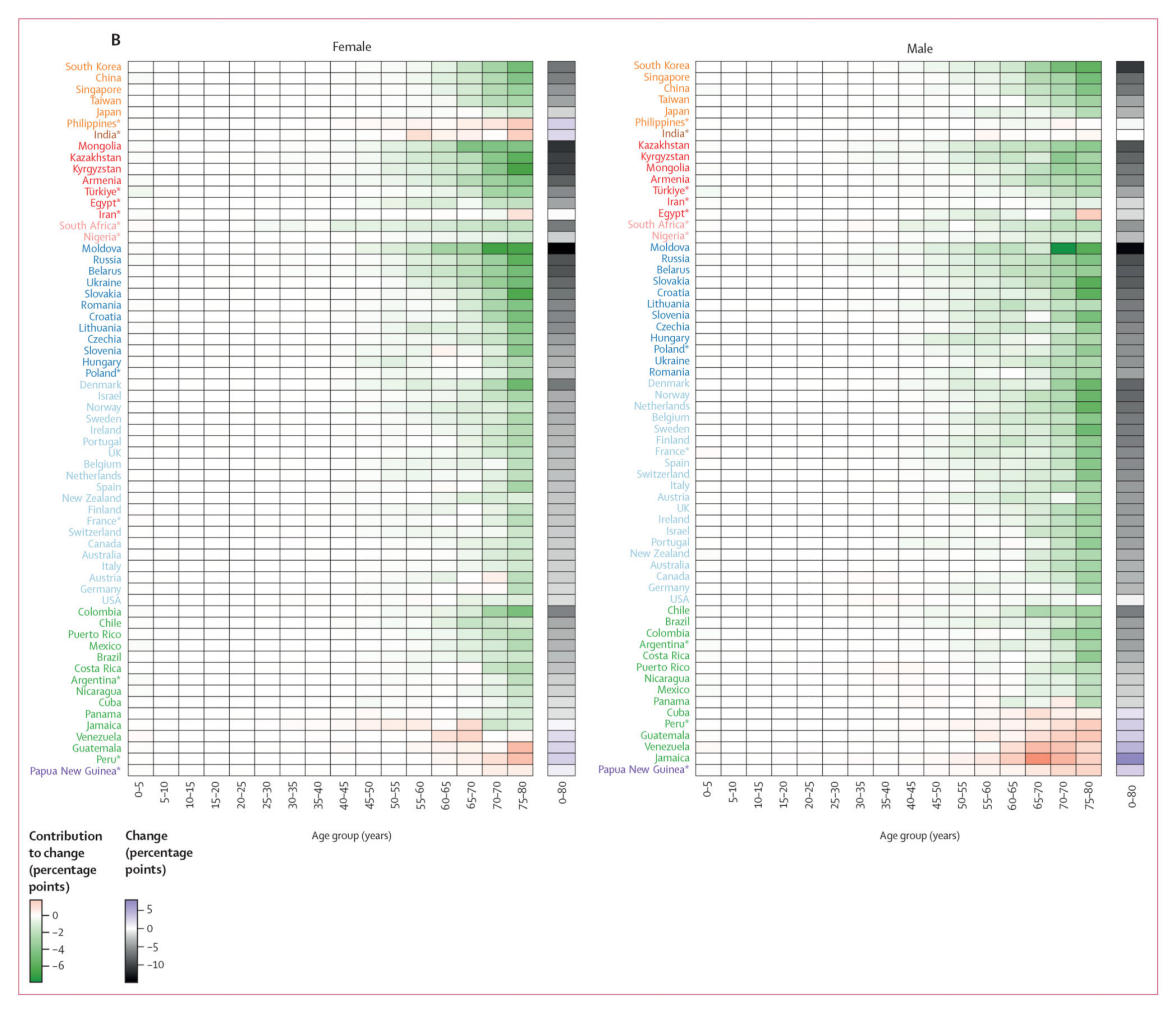
Contributions of mortality from different NCD causes of death and in different age groups to overall change in NCD mortality from 2010 to 2019 (A) The contribution of 20 mutually exclusive, collectively exhaustive NCD causes of death to the change in the probability of dying from an NCD between birth and age 80 years from 2010 to 2019. Each column represents a cause of death, as stated in the appendix (pp 11–12), with causes arranged by disease category. Results are presented for aggregated cause groups in the appendix (pp 35–36). (B) The contribution of 5-year age groups to the change in this probability over the same period, with each column representing a 5-year age group. In both panels, each row represents a country. Results are shown for 63 countries, of which 51 were identified as having high-quality data and 12 were selected based on population size. Countries are grouped and coloured by region and ordered from the largest decrease to the smallest decrease or largest increase in the probability of dying from an NCD between birth and age 80 years from 2010 to 2019. Each tile shows the absolute contribution of an NCD cause of death or age group to the total change in this probability for one country. Two colour palettes are used: one for the overall change in NCD mortality from 2010 to 2019, and one for contributions of individual NCD causes of death or age groups. For overall change, black indicates a decrease, purple an increase, and white no change. For contributions to change, green indicates a contribution to lowering NCD mortality, red a contribution to increasing it, and white a contribution of zero. For numerical results see the appendix (pp 26–28). For results based on cancers, cardiovascular diseases, chronic respiratory diseases, and diabetes in ages 30–70 years see the appendix (pp 58–60). NCD=non-communicable disease. *12 countries selected based on population size.

Of the 63 countries for which NCD causes of death were analysed, the probability of dying from an NCD before age 80 years decreased in 55 (87%) countries for females and 56 (89%) countries for males; probability of dying increased in the remaining countries. In 39 (62%) of 63 countries for females and in 38 (60%) of 63 for males, ischaemic heart disease was the largest contributor to declining NCD mortality (figure 7A), contributing up to 7·9 percentage points towards lowering the probability of dying from an NCD (out of a total of 14·8 percentage points) for females in Moldova. Despite this favourable change in most countries, ischaemic heart disease mortality increased in eight countries for females and in nine for males. These countries included Papua New Guinea and some countries in Asia and Latin America and the Caribbean. In most of these countries, rising mortality from ischaemic heart disease led to an overall increase in NCD mortality. Only three countries (Mexico and Nicaragua for both sexes, and the Philippines for males) had a decline in total NCD mortality despite an increase in ischaemic heart disease mortality. Of countries with declining NCD mortality, stroke was the second largest contributor in 34 (54%) of 63 countries for females and 21 (33%) of 63 countries for males. The contribution of stroke to the decline in NCD mortality was particularly large in central and eastern Europe, where it ranked the second largest contributor to decline in most countries. In high-income western countries, stroke was the second greatest contributor among females in 12 of 20 countries, and among males in two of 20 countries.

Taken together, cancers contributed towards lowering NCD mortality in 51 (81%) of 63 countries for females and 55 (87%) of 63 for males (appendix pp 35–36). Among cancers, trachea, bronchus, and lung cancer (referred to as lung cancer hereafter) mortality was an important contributor to how overall NCD mortality changed, with variable changes between males and females and across countries. Lung cancer mortality declined in 58 (92%) of 63 countries for males; the largest contribution of lung cancer to overall reduction in the probability of dying from an NCD for males was seen in the Netherlands (1·7 percentage points), followed by in Belgium and Singapore. Lung cancer increased in only five (8%) of 63 countries (India, Armenia, Iran, Egypt, and Papua New Guinea) for males. Lung cancer was the largest contributor to the decline in male NCD mortality in the USA and the second largest in 15 of 19 other high-income western countries. Among females, lung cancer mortality improved (ie, augmented a decline or diminished an increase) NCD mortality in 34 countries (54%) of 63 and worsened NCD mortality (ie, augmented an increase or diminished a decline) in 29 (46%) countries. The largest contributions of lung cancer to reductions in female NCD mortality were seen in Denmark (0·8 percentage points), the USA (0·8 percentage points), and Canada (0·6 percentage points). Increases in female lung cancer mortality occurred in central and eastern Europe (Hungary, Poland, Croatia, Slovenia, Lithuania, Romania, Czechia, and Slovakia), in some high-income western countries (Austria, Germany, Spain, France, Switzerland, Portugal, Italy, Belgium, and Finland), and in some countries in Latin America (Argentina, Chile, Brazil, Peru, and Cuba), where lung cancer either diminished the decline in national overall NCD mortality or amplified its increase. The contributions of chronic obstructive pulmonary disease (COPD) and lung cancer to the decline in total NCD mortality generally were in the same direction, but weakly correlated (correlation coefficients of 0·33 for females and 0·05 for males). Colon and rectum (colorectal) cancers declined in 43 (68%) of 63 countries for females and 40 (63%) of 63 countries for males, with mean contributions to NCD mortality decline of 0·2 percentage points for females and 0·3 for males. In contrast to mostly favourable changes in lung and colorectal cancers, pancreatic cancer mortality increased in 44 (70%) of 63 countries for both sexes, albeit with a relatively small mean contribution of 0·05 percentage points to worsening overall NCD mortality.

Neuropsychiatric conditions increased and contributed unfavourably to NCD mortality change from 2010 to 2019 (ie, attenuated the decline or magnified the increase) in most countries, particularly in high-income western countries and central and eastern European countries (appendix pp 35–36). Mortality from Alzheimer disease and other dementias increased slightly in 41 (65%) of 63 countries for females and 43 (68%) of 63 countries for males, with a mean contribution of 0·2 percentage points towards higher NCD mortality for both sexes. These countries included 18 (90%) of 20 high-income western countries for each sex. Alcohol use disorders contributed unfavourably to male NCD mortality in 31 (49%) of 63 countries, with the largest contribution in Slovenia (1·2 percentage points), followed by Slovakia and Poland.

Finally, diabetes (including chronic kidney disease due to diabetes) contributed to an increase in NCD mortality or diminished its decline in 23 countries (37%) of 63 for females and in 27 (43%) of 63 countries for males, with a mean contribution of 0·4 percentage points for both sexes. Diabetes contributed to a decline in mortality in most high-income western countries (except the USA and UK for both sexes and Finland for females), in high-income and emerging economies in east Asia (China, Japan, Singapore, South Korea, and Taiwan), and in lower-mortality nations in Latin America such as Mexico, Chile, Brazil, and Colombia, while contributing to an increase in most other countries including others in Latin America and the Caribbean.

For both females and males, mortality in ages 65 years and older made the largest contributions to reductions or increases in the probability of dying from an NCD from 2010 to 2019 because death rates are higher at older ages (figure 7B; appendix pp 21–23). Where large NCD mortality reduction was not achieved or where NCD mortality in those aged 65 years and older increased, the overall probability of dying from an NCD before age 80 years either increased (India, Peru, Guatemala, Venezuela, Jamaica, and Papua New Guinea for both sexes, and the Philippines and Iran for females) or improved little (the USA and Panama for both sexes, Austria and Germany for females, and Egypt for males). In some countries, this was augmented by an increase in mortality in working ages (eg, India, the Philippines, and Peru for both sexes, and Jamaica for males). At the same time, a distinguishing difference between countries with larger versus smaller declines was that those with larger declines (eg, South Korea, Mongolia, Kazakhstan, South Africa, Moldova, Denmark, Norway, and Chile) saw noticeable reductions in both older (≥65 years) and working ages, whereas those with smaller declines did not have these reductions in working-age mortality (eg, both sexes in Canada and Poland, and males in Mexico and Australia) or even had an increase (eg, females in Jamaica) despite the oldest ages showing a decline in mortality.

### Comparison of change from 2010 to 2019 with change from 2001 to 2010

Although NCD mortality decreased in most countries from 2010 to 2019, there was widespread slowdown or reversal of progress as compared with the previous decade. Specifically, females in 84 (45%) of 185 countries and males in 80 (43%) of 185 countries had a smaller decline in NCD mortality from 2010 to 2019 than they had from 2001 to 2010 (figures 3, 4; appendix pp 24–25).

In a further 24 (13%) countries for females and 27 (15%) countries for males, mortality declined from 2001 to 2010 but increased from 2010 to 2019, leading to a reversal of progress. In particular, the decline in NCD mortality was smaller from 2010 to 2019 than from 2001 to 2010 for high-income western countries, except for females in Denmark and Iceland and for males in Finland. Many countries in Latin America and the Caribbean also had a slowdown or reversal of NCD mortality decline, although in others, including Chile and Mexico for both sexes and Brazil for males, the decline in NCD mortality accelerated. Similarly, there was a deterioration in the direction or size of NCD mortality change for one or both sexes in most countries in east and southeast Asia, including for both sexes in Japan and South Korea, and for females in China. A similar deterioration was seen for females in most countries in south Asia, including India, where NCD mortality declined from 2001 to 2010 but increased from 2010 to 2019 (panel 2). When we used deaths from cancers, cardiovascular diseases, chronic respiratory diseases, and diabetes for ages 30–70 years, as used in SDG target 3.4, an even larger number of countries had a slowdown or reversal of progress in reducing NCD mortality from 2010 to 2019 compared with from 2001 to 2010 (appendix pp 13–14).

In contrast to these slowdowns and reversals, the decline in NCD mortality accelerated in 46 countries for females and 40 for males, most commonly in countries in central and eastern Europe (eg, Moldova and Russia), central Asia (eg, Azerbaijan and Uzbekistan), and parts of the Middle East and north Africa (eg, Qatar). For males, many of the countries in central Asia and eastern Europe with large declines from 2010 to 2019 had recorded minimal or no improvement in the preceding decade. In the remaining 22 countries for females and 27 for males, NCD mortality increased from 2001 to 2010 but declined in the subsequent decade. Examples of countries with high-quality mortality that had such a turn-around include Nicaragua for both sexes, and Kyrgyzstan, Armenia, and Mexico for males.

**Figure 8:**
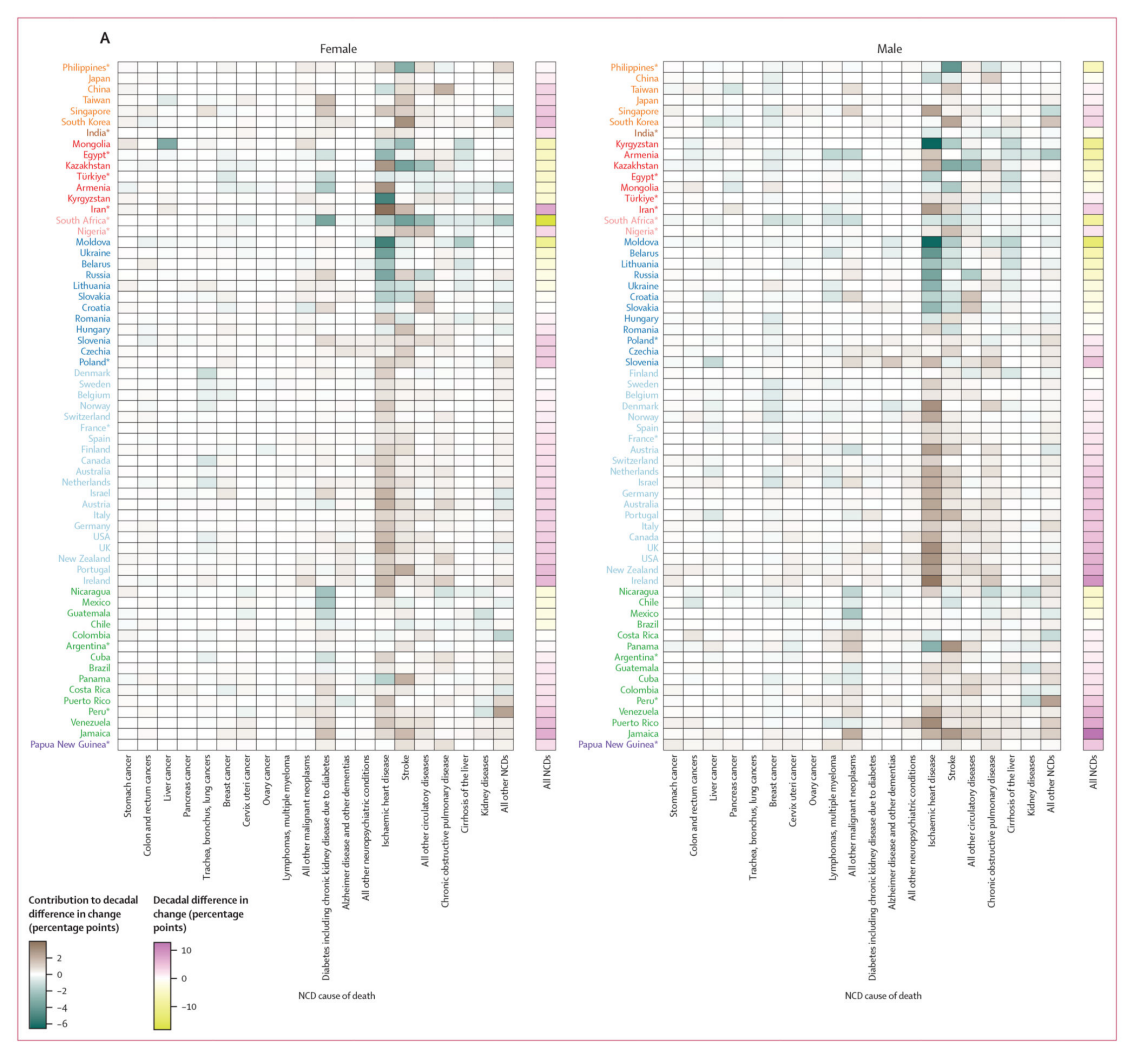

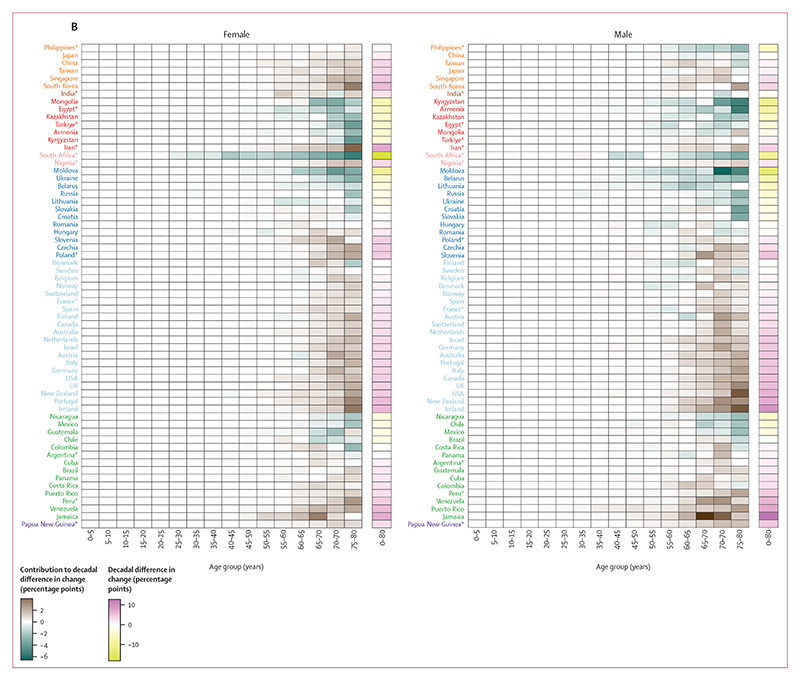
Contributions of mortality from different NCD causes of death and in different age groups to slowdown or acceleration of change in NCD mortality from 2010 to 2019 compared to change from 2001 to 2010 (A) The contribution of 20 mutually exclusive, collectively exhaustive NCD causes of death to the difference in change in the probability of dying from an NCD between birth and age 80 years between two decades (2010–19 and 2001–10). Each column represents a cause of death, as stated in the appendix (pp 11–12), with causes arranged by disease category. Results are presented for aggregated cause groups in the appendix (pp 37–38). (B) The contribution of 5-year age groups to this decadal difference in change, with each column representing a 5-year age group. In both panels, each row represents a country. Results are shown for 63 countries, of which 51 were identified as having high-quality data and 12 were selected based on population size. Countries are grouped and coloured by region and ordered from the largest improvement in NCD mortality from 2010 to 2019 compared with the preceding decade to the largest deterioration. Each tile shows the absolute contribution of a specific NCD cause of death or age group to the decadal difference in change in NCD mortality (ie, difference in change between the two decades) for one country. Two colour palettes are used: one for the overall decadal difference in change, and one for contributions of individual NCD causes of death or age groups. For overall decadal difference in change, yellow indicates improvement (a larger decline, smaller increase, or reversal of an increase), pink indicates deterioration (a smaller decline, reversal of a decline, or a larger increase), and white indicates no difference in the magnitude of change. For contributions to decadal difference in change, green indicates a contribution to improvement of change (a larger decline, smaller increase, or reversal of an increase), brown indicates a contribution to deterioration of change (a smaller decline, reversal of a decline, or a larger increase), and white indicates a contribution of zero. For numerical results see the appendix (pp 29–31). For results based on cancers, cardiovascular diseases, chronic respiratory diseases, and diabetes in ages 30–70 years see the appendix (pp 61–63). NCD=non-communicable disease. *12 countries selected based on population size.

The slowdowns or reversals of the decline in NCD mortality from 2010 to 2019 compared with the preceding decade (from 2001 to 2010) were a result of deteriorations in the direction or size of change in multiple NCD causes of death (figure 8A). For example, in high-income countries that had a slowdown of NCD mortality decline, the only causes of death that had more favourable changes from 2010 to 2019 compared with the preceding decade in more than half of the countries analysed were lung cancer for both sexes, ovarian cancer for females, and colorectal cancer and the residual group of all other malignant neoplasms for males. Similarly, in countries showing the greatest improvement in performance from 2010 to 2019 compared with the preceding decade, such as Moldova and South Africa, the acceleration of NCD mortality decline was seen across all or most causes of death.

Ischaemic heart disease and stroke, which contributed the most to NCD mortality decline from 2010 to 2019, were also the largest contributors to decadal shifts in how much overall NCD mortality changed, both in countries where progress accelerated compared with the preceding decade (many countries in central Asia and central and eastern Europe) and in those where progress slowed or reversed (most other countries). The changes in these two conditions deteriorated, typically in the form of a slowdown in their decline, in approximately two-thirds of the 63 countries for both sexes, with the notable exception of countries in central and eastern Europe and in central Asia. All high-income western countries performed worse for ischaemic heart disease and stroke from 2010 to 2019 than in the previous decade for both females and males, which was the opposite of the pattern in central and eastern Europe, where these conditions declined more from 2010 to 2019 than from 2001 to 2010.

Total cancer mortality showed improvement (larger declines or smaller increases) from 2010 to 2019 compared with the preceding decade in 26 (41%) of 63 countries for females and 38 (60%) countries for males, while progress deteriorated in the remaining 37 (59%) countries for females and 25 (40%) countries for males (appendix pp 37–38). Lung cancer was an important driver of improvement in how much cancer mortality changed for males; lung cancer mortality had a more favourable change from 2010 to 2019 than in the preceding decade in 45 (71%) of 63 countries. Changes in colorectal cancer mortality for males from 2010 to 2019 were also an improvement (ie, a faster decline or a slower increase) compared with changes from 2001 to 2010 in 38 (60%) of 63 countries. For many other cancer types (eg, stomach, breast, cervix, and upper aerodigestive tract cancers, and lymphomas and multiple myeloma), the declines slowed down or reversed or the pace of their increase accelerated from 2010 to 2010 compared with the preceding decade in more than half of the 63 countries analysed. Deteriorations in the direction or size of change were seen for COPD in 45 (71%) of 63 countries for females and 39 (62%) countries for males, which is a more widespread deterioration of change than that of lung cancer. Mortality from kidney diseases, liver cirrhosis, diabetes (including those from diabetes-related chronic kidney disease), pancreatic cancer, lung cancer for females, and prostate cancer showed a mix of acceleration and deceleration of decline or increase between the two decades.

As was the case for contributions to the change in mortality from 2010 to 2019, older age groups contributed the most to decadal improvement or deterioration, with those 65 years and older accounting for nearly three-quarters of how changes differed from 2010 to 2019 compared with from 2001 to 2010 (figure 8B). There were nonetheless countries whose decadal improvement or deterioration was affected noticeably by changes in working ages. For example, in some Nordic countries (Finland and Norway for both sexes, and Denmark for males), the decline in NCD mortality slowed down in older ages from the first to the second decade of the millennium, but was countered by acceleration in working ages, limiting the overall slowdown and helping them maintain good performance relative to their comparators. More generally, where declines in NCD mortality had large accelerations (some countries in central Asia and central and eastern Europe) or slowdowns (some English-speaking high-income western countries such as Ireland, New Zealand, UK, and USA; some countries in Latin America and the Caribbean; South Korea; and Singapore), the large magnitude was driven by decadal improvement or deterioration in both working and older ages.

### Comparison with regional benchmarks

**Figure 9:**
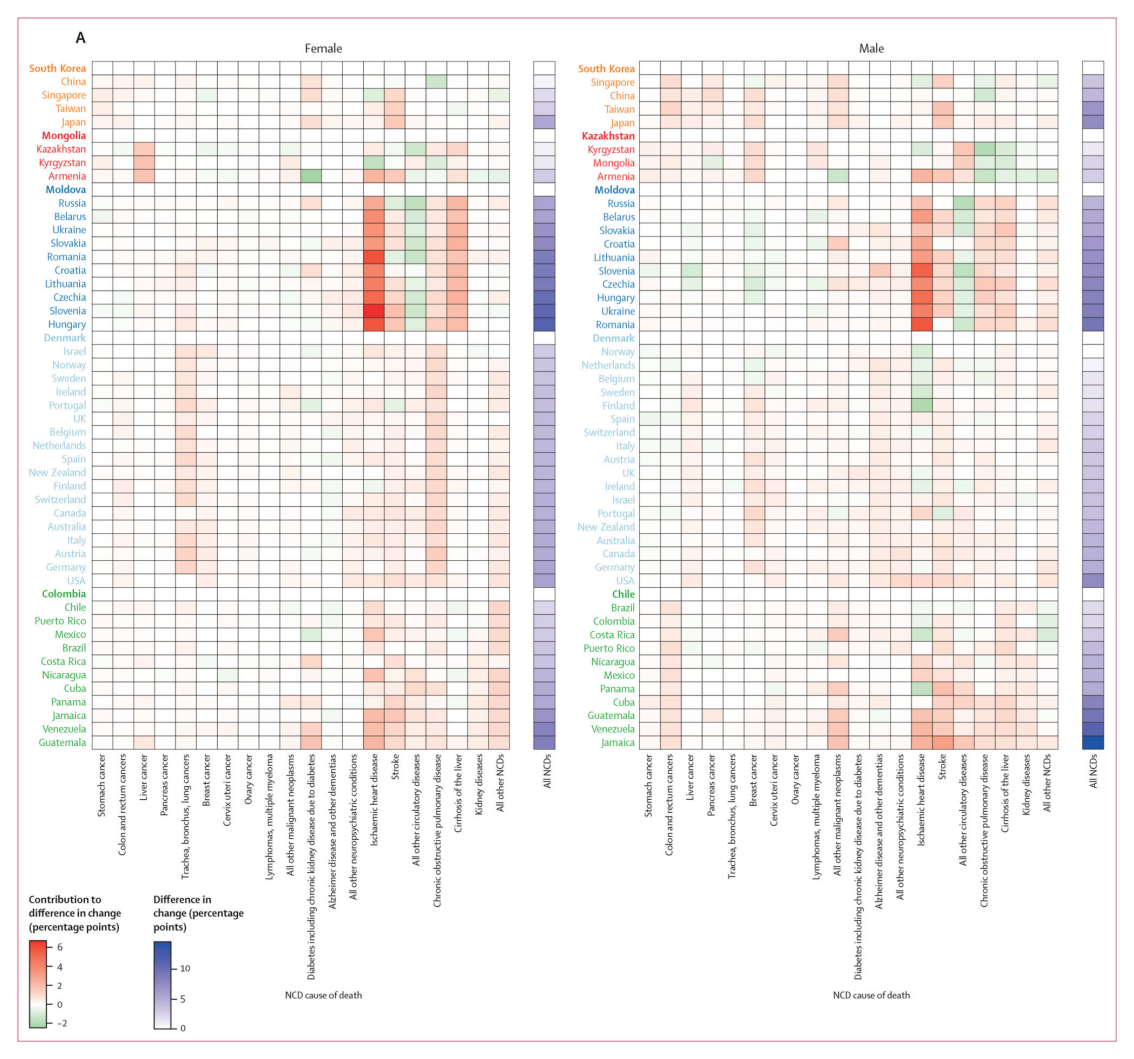

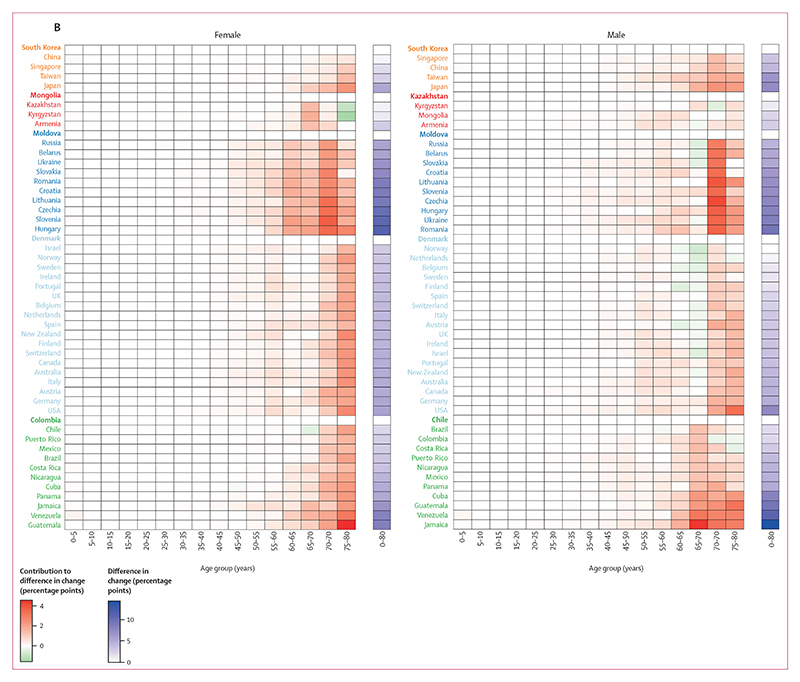
Contributions of mortality from different NCD causes of death and in different age groups to how much NCD mortality in each country lags its regional benchmark (A) The contribution of 20 mutually exclusive, collectively exhaustive NCD causes of death to the difference in the change in the probability of dying from an NCD between birth and age 80 years from 2010 to 2019, compared with a country benchmark within each region. Benchmarks are identified as the country in each region with the largest reduction in NCD mortality over this period. Each column represents a cause of death, as stated in the appendix (pp 11–12), with causes arranged by disease category. Results are presented for aggregated cause groups in the appendix (pp 39–40). (B) The contribution of 5-year age groups to this difference, with each column representing a 5-year age group. In both panels, each row represents a country. Results are shown for 51 countries identified as having high-quality data. Countries are grouped and coloured by region and ordered from the largest decrease to the smallest decrease or largest increase in the probability of dying from an NCD between birth and age 80 years from 2010 to 2019. The benchmark for each region is the country in the first row of its region grouping and is shown in bold font. Each tile shows the absolute contribution of a specific NCD cause of death or age group to the difference in change compared with the benchmark country for one country. Two colour palettes are used: one for the overall difference in change compared with the benchmark, and one for contributions of individual NCD causes of death or age groups compared with those of the benchmark. For overall difference in change, black indicates a decrease compared with the benchmark, purple indicates an increase compared with the benchmark, and white indicates no difference in change. For contributions to difference in change, green indicates a contribution towards a larger decline or smaller increase compared with the benchmark, red indicates a contribution towards a smaller decline or larger increase compared with the benchmark, and white indicates a contribution of zero. For numerical results see the appendix (pp 32–34). For results based on cancers, cardiovascular diseases, chronic respiratory diseases, and diabetes in ages 30–70 years see the appendix (pp 64–66). NCD=non-communicable disease.

We restricted the analysis in questions 3a and 3b to countries with high-quality data and to regions with more than two countries included in the cause-decomposition analysis, and selected the country with the largest reduction in NCD mortality from 2010 to 2019 within each region as a benchmark. The countries that emerged as benchmarks were South Korea (east and southeast Asia), Moldova (central and eastern Europe), and Denmark (high-income western countries) for both sexes, Mongolia (central Asia, Middle East and north Africa) and Colombia (Latin America and the Caribbean) for females, and Kazakhstan (central Asia, Middle East and north Africa) and Chile (Latin America and the Caribbean) for males. A feature of these countries was that within their regions they had exemplary performance (ie, large reductions or small increases) across all or most NCD causes of death and age groups (figure 9). There were a few exceptions to benchmarks’ across-the-board excellence. Many high-income western countries had greater reductions in stomach, pancreatic, and upper aerodigestive tract cancer mortality than the regional benchmark, Denmark. In central Asia for males, some countries performed better than the regional benchmark, Kazakhstan, in reducing liver cirrhosis, COPD, and kidney diseases. In central and eastern Europe, some countries performed better than Moldova in reducing deaths from lung cancer and from the aggregate category of all other circulatory diseases (for which the role of differences in cause of death assignment or coding cannot be ruled out).

## Discussion

Our analysis of NCD mortality in the current millennium shows that NCD mortality declined from 2010 to 2019 in four of every five countries in the world, covering much of the world’s population. Our results also revealed complex and important patterns within this general success story. First, there was substantial variability in the size of decline. Good and poor performance from 2010 to 2019 was unrelated to the level of NCD mortality in 2010, indicating that small reductions were not due to mortality having already reached low levels and, conversely, large reductions were not more likely in countries with high mortality, where it might have been expected to be easier to reduce some deaths. This finding also meant that, despite improvements, there was little or no evidence of convergence in NCD mortality, globally and in most regions, especially for females. As a result, substantial global disparities in NCD mortality persisted in 2019.

Second, in almost two-thirds of the countries, including in nearly all high-income countries in Europe, north America, Australasia, and east Asia, the decline from 2010 to 2019 was slower than in the preceding decade or there was even a reversal of the earlier decline. The notable exceptions to this pattern were most countries in central and eastern Europe and central Asia. Finally, within all regions, there were substantial performance gaps between the regional frontrunner and other countries. These two phenomena—slowdown of mortality decline and lagging regional best-performers—were affected by most NCD causes of deaths. Variations in how much NCD mortality declined, and its decadal improvements or deteriorations, were driven by the magnitude of declines at older ages, for which death rates were highest, and by whether countries saw declines, stagnation, or increases in mortality at working ages.

## Strengths and limitations

The primary strength of our paper is the systematic and comprehensive analysis of changes in NCD mortality in an era of significant political attention to NCDs. We systematically analysed the contributions of deaths from specific NCDs and in specific age groups to changes in total NCD mortality. We took an inclusive approach to NCDs and age, including age groups and conditions (such as kidney and liver disease and dementia) that were excluded from global targets, even though they are associated with high disease burdens, especially in low-income and middle-income countries, are aetiologically related to conditions included in the targets, and are in part preventable and treatable.

The main limitation of our work and of other global analyses of mortality is the limitations of data on total and, especially, cause-specific mortality. Fewer than one in three countries had complete death registration with high-quality cause-of-death assignment.^9^ Data limitations were particularly widespread in Pacific Island nations and in countries in sub-Saharan Africa and south Asia, where 90% of countries in these regions (54 of 60) had low-quality or very low-quality data. If too few deaths are registered, or if the quality of cause-of-death information is too poor, death registration data cannot directly be used to reliably monitor mortality, especially by cause. In such countries, demographic and epidemiological data and methods are used to estimate all-cause and cause-specific mortality,^9^ leading to substantial uncertainty. For this reason, we limited the analysis of cause-specific mortality to those countries with high-quality data, which were predominantly high-income nations and emerging economies, with additional presentation of only the largest countries in each region which are clearly marked in presenting these results. Even where death registration is complete and cause of death data are evaluated as high-quality, errors can remain because assignment of cause death depends on how much information is available to the certifier and their choices about the sequence of events that led to death. Therefore, a priority area for strengthening accountability towards NCDs should be expanding and strengthening death registration, including high-quality medical certification of cause of death. Additionally, there is year-to-year variability in mortality, especially in small populations, which means change between 2 years, as experienced by each population, might be slightly different from long-term trends estimated using a linear fit or other functions of time.

We used the probability of dying from an NCD between birth and age 80 years as our measure of mortality. This metric has multiple advantages: it is intuitive and interpretable; it is not affected by differences in mortality from conditions other than NCDs; and it does not require the use of an (arbitrary) external standard population for standardisation. However, it does not consider variations in mortality across countries beyond age 80 years nor does it consider morbidity, which causes substantial loss of healthy life-years for some NCDs. NCDs also affect deaths from conditions that are classified as injuries (eg, psychiatric conditions increase the risk of suicides) or infectious diseases (eg, diabetes and possibly COPD increase the risk of tuberculosis^10^ and COVID-19),^11,12^ which are not included in estimates of NCD mortality because the International Classification of Diseases system requires each death to be assigned to a single underlying cause. In particular, deaths from suicides are also an indicator for SDG target 3.4, but are included in injuries and not NCDs in global mortality statistics.^7^ Finally, as stated below, there are currently limited data on disease incidence, prevalence, and survival, and on the technical and policy interventions that affect them, to analyse the role of programmes and policies in the observed changes and their variations across countries and decades.

### The reasons for widespread decline in NCD mortality

Mortality from NCDs is an outcome of the incidence (for cancers and acute conditions such as myocardial infarction and stroke) or prevalence (for chronic conditions such as diabetes, chronic respiratory diseases, kidney disease, and dementia) of a condition and its survival. NCD incidence, prevalence, and survival are affected by health care and by protective and harmful environmental, nutritional, psychosocial, and behavioural factors. Health-care access and quality and protective and harmful determinants of NCDs are distributed unevenly both across and within countries,^13^ and change over time. Precise attribution of the observed declines, and their variations across countries, requires substantially more data than currently available on disease incidence, prevalence, and survival, and on the technical and policy interventions that affect them, with repeated measurements over time. In the absence of such data, the explanations for these changes must draw on the broader evidence on intervention and policy impacts and implementation.

The 20th century saw a substantial rise in knowledge of NCD aetiology and pathophysiology, followed by clinical and public health interventions that were verified in randomised trials or rigorous observational studies.^14–20^ These interventions were subsequently incorporated in clinical guidelines and influenced clinical practice in primary care and at the hospital level; they also led to policies that reduced some of the harmful factors that contribute to NCDs. For diabetes and cardiovascular and renal diseases, randomised trials of antihypertensive and cholesterol-lowering and glucose-lowering medicines^15,21–37^ for primary and secondary prevention of cardiovascular disease and delaying end-stage renal disease led to changes in clinical guidelines and practice before and around the turn of the millennium, and the thresholds for treatment initiation and targets became progressively more ambitious (ie, lower).^19^ The use of these medicines increased, initially in high-income countries and then in some emerging economies and middle-income countries with strong health systems.^6,38,39^ Public awareness campaigns, which led to earlier recognition and hospital arrival for patients with myocardial infarction, and the introduction of more sensitive diagnostic assays, reduced time to treatment, whereas revascularisation and the establishment of coronary care units improved the survival of hospitalised patients.^14,19,40–55^ Similar benefits were achieved for stroke via better acute management protocols and the establishment of specialist stroke units that increased timely thrombolysis, mechanical thrombectomy and post-stroke blood pressure management.^56–59^ These benefits were augmented by those from secondary prevention through the use of antihypertensive medications, statins, and low-dose aspirin.^19,40,60^

For cancers, the demonstrated benefits of screening^61^ for detecting pre-cancerous lesions and early-stage cancers (eg, for cervical, breast, prostate, and colon cancers) led to clinical guidelines and programmes expanding coverage, which in turn lowered incidence and improved survival through diagnosis in earlier stages.^62–68^ There were also improvements in treatment for several cancers which further improved survival.^69,70^ The establishment of the role of infections as a cause of multiple cancers^16,71,72^ was followed by successful development of vaccines, such as those against hepatitis B virus and human papillomavirus.^73–75^ Although most of the benefits of vaccines for reducing cancer deaths will be realised over time, some deaths have already been averted through their use.^76,77^

The major reductions in mortality from NCDs in high-income countries and in emerging economies and middle-income nations in Asia and Latin America have been at least partly a result of health system programmes that rigorously and systematically evaluated the evidence on the benefits of innovations in diagnosis and treatment of specific conditions, and leveraged and adapted the primary and specialist care systems so that they reached the people who could benefit from screening or treatment.^78–90^ The variations across countries may be due to how these programmes were implemented, and the resulting effect on the coverage and quality of evidence-based interventions.

In terms of policies, the evidence on the harms of tobacco for a range of NCDs led to the implementation of control measures, including taxes, regulation of sales, advertising and promotion, and indoor smoking bans, starting in some high-income countries. These policies were evaluated both prospectively (through economic and legal analyses) and after implementation in terms of their effects on prevalence, initiation, and cessation, and underpinned global tobacco control efforts.^91,92^ As a result of these measures, tobacco use declined substantially, initially in high-income western countries (especially English-speaking countries and those in northwestern Europe) and subsequently in middle-income countries with strong control measures. These trends, and the variations in their timing across countries and sexes, likely underlie the decline in lung cancer for males in these countries through the two decades, and the fact that females had a mix of declines and increases in lung cancer because the uptake and decline in smoking occurred later in females than in males.^93^ The variations in timing might also be partly responsible for the slower decline in NCD mortality for females than for males in high-income western countries (appendix pp 19–20). Evidence also accumulated for effectiveness of fiscal and regulatory policies to reduce (harmful) alcohol use, including increases in alcohol excise taxes and availability restrictions.^94–96^ In particular, alcohol use was a key cause of high mortality from NCDs in many countries of the former Soviet Union in eastern Europe and central Asia in the 1990s and 2000s.^97–101^ Some of these countries initiated comprehensive alcohol control policies at the end of the first decade and in the second decade of this millennium; these policies contributed to marked declines in alcohol consumption and to a larger decline in NCDs over this time period.^102–106^ Additionally, regulations related to air pollution^107–109^ and occupational pollutants^16,110^ helped reduce the NCDs caused by these harmful exposures.

Alongside these clinical and policy interventions, secular economic trends and technological advances might have contributed to the reductions seen in our results, especially in middle-income countries and emerging economies that had significant economic growth and infrastructure expansion in the late part of the 20th century. Among these, improvements in sanitation, water, hygiene, and food security with economic development led to fewer infections and better foetal and early-life nutrition with likely benefits for a range of NCDs such as cervical, stomach, and liver cancers and cardiometabolic diseases.^18,19,71,111^ Urbanisation, electrification, and expansion of transport networks enhanced more regular availability of fresh foods, especially fruits and vegetables,^112,113^ which were traditionally seasonal, reduced the risk of food contamination and the need to use salt to preserve food, and improved access to health care, all of which would in turn help to reduce multiple NCDs.^18,19,114–116^

Although data for quantitative attribution are currently scarce, some countries provide case studies of pathways to good performance. For example, South Korea had the fifth largest decline in NCD mortality in the world for females and the third largest for males from 2001 to 2010 (and the single largest among countries with high-quality data) and continued impressive declines from 2010 to 2019 despite a slowdown (panel 2). South Korea rolled out universal health insurance in the last part of the 20th century,^117,118^ and set up national programmes that not only translated knowledge and technologies related to disease aetiology, diagnosis, and treatment into clinical practice in primary and secondary health care but also ensured that these advances benefited the entire population. South Korea has the largest number of contacts with primary care providers among the Organisation for Economic Co-operation and Development countries, and a rigorously designed and evaluated nationwide programme for screening that increased the diagnosis of multiple conditions.^119,120^ This has made South Korea one of the leading countries in diagnosis and treatment of conditions such as hypertension and diabetes alongside good performance in cancer screening.^6,38,39,79,120,121^ South Korea also leveraged its rapid economic growth towards substantial broad-based improvements in education, housing, infrastructure, and early-life nutrition that addressed many of the environmental and nutritional determinants of NCDs.^117,122,123^ Finally, South Korea has lower health inequalities than some of its western counterparts, and has constrained or even lowered inequalities across its districts, in contrast to countries such as the UK and USA.^13,124–126^ These mechanisms exemplify those used by other countries with good performance in NCDs, such as hypertension or diabetes diagnosis and treatment in Costa Rica, Chile, Kazakhstan, and Finland,^83–87^ and cancer prevention, screening, and treatment in Denmark and Chile.^89,90^ Unlike South Korea, there has been no clear explanation for the impressive decline in NCD mortality in Moldova, which has also been recorded in other works.^127^ These works have shown that the national decline has been accompanied by increasing urban–rural disparity.^127^ In neighbouring Russia and some other countries in eastern Europe, reduction in alcohol use following control policies was a key factor leading to mortality decline.^97,102–106^

### The slowdown and reversal of decline

The precise reasons for the slowdown or reversal of the decline from the first to the second decade of the millennium in the majority of countries also requires the aforementioned data on disease incidence, prevalence, and survival, and on the interventions and policies that affect them with repeated measurements over time, which are not currently available. The broader evidence on interventions and policies collectively indicates that these deteriorations occurred either because the increase in the coverage of evidence-based interventions stagnated, and in some cases reversed, during the second decade of our analysis, or because these interventions did not reach the segments of population that account for most deaths and are in need of prevention and treatment. For example, in many high-income countries, the roll-out of antihypertensive medicines plateaued around 2010, at varying levels that were below the best-performing national and regional hypertension programmes,^39^ with the slowdown occurring at even lower coverage levels in most low-income and middle-income countries.^38^ A similar slowdown occurred for diabetes treatment, breast and cervical cancer screening (contrasting with colorectal cancer screening, which has increased), and myocardial infarction survival.^6,47,49,68,120,128–133^ These trends might be partially responsible for the observed slowdowns in the decline of cardiovascular diseases and some cancers. In terms of policies, although some countries have continued with stringent tobacco control, contributing to continued declines in lung cancer and other conditions, the momentum of the 1990s and 2000s seems to have been lost elsewhere, or these policies have not kept pace with the changing tactics of the tobacco industry.^134–136^ Similarly, weakening of effective alcohol control measures, such as those implemented by Nordic countries’ (Finland, Iceland, Norway, and Sweden) alcohol monopolies,^137^ might have contributed to the slowdown of the decline in mortality from some NCDs.

Several trends, which collectively amount to changes in resources and prioritisation, might have contributed to a stagnation or reversal in the expansion of clinical interventions and fiscal and regulatory policies for NCDs, or to whether they reached those who needed them the most. First, macroeconomic responses to the 2008 global recession curtailed the expansion of health budgets and development assistance for health in many countries which, together with population growth and ageing, have constrained the availability of services for prevention and treatment.^138–143^ Second, the responses to the 2008 global recession also led to a rise in poverty, destitution, and insecure employment.^144–151^ The poor and those with lower education and insecure employment, who account for a disproportionately large share of deaths relative to their share of the population, typically have less access to and utilisation of NCD clinical interventions and beneficial factors such as fresh, healthy foods.^13,152–157^ This inequality in access might have acted synergistically with lower-than-expected spending on health and contributed to the slowdown of declines in NCD mortality, which was nearly universal in high-income western countries.^158–161^ Third, the number of policy recommendations for control of NCDs has increased substantially. However, few large-scale policy experiences have been rigorously evaluated and, of those that have, only a handful of the commonly recommended policies show a more-than-negligible effect on epidemiologically relevant and clinically relevant outcomes; beyond the aforementioned tobacco and alcohol control policies, those with stronger impacts are: air pollution regulations, comprehensive indoor smoking ban, tax on sugar sweetened beverages, trans fat bans, and comprehensive salt reduction programmes, all of which involve fiscal and regulatory components.^78,107,109,162–180^ The growing number of recommendations with varying levels of evidence makes prioritisation and efficient resource allocation difficult. Fourth, although some fiscal and regulatory responses to the commercial determinants of NCDs have been strengthened, others might have been weakened or not kept pace with the changing tactics of industry, such as those mentioned earlier for tobacco and alcohol use.^136,137^ Finally, the shifting focus of health system discourse onto universal health coverage might have helped remove or lower the financial barriers to care, but has not been accompanied by sufficient emphasis on high-quality programmes that improve the coverage of evidence-based interventions for timely diagnosis and treatment of NCDs through guidelines, training, decision support, equipment, and procurement and distribution of medicines. Without such programmes, the effects of universal health coverage on uptake of NCD preventive interventions and treatment and their mortality benefits are limited, especially in low-income countries where the health systems and services have been largely focused on infectious diseases and maternal and child health.^181–184^ Strengthening these programmes, and organising and resourcing primary care to effectively integrate them and ensure their quality is fundamental to successful prevention, treatment, and management of NCDs as universal health coverage is rolled out.^185–188^

### Reviving accelerated declines

Our results show that reducing NCD mortality is feasible in national populations, even in countries with low baseline mortality. Such reductions were achieved in the decade leading to the COVID-19 pandemic in most countries, and were even larger in the first decade of the millennium. The broader evidence, from clinical medicine, epidemiology, health systems, and public health, indicates that these successes were likely due to roll-out of effective clinical interventions in well designed national programmes and fiscal and regulatory policies, which were especially effective where and when they were rigorously evaluated and adjusted to improve quality and reach those who needed them. These results, and their explanations, raise an imperative for the upcoming Fourth High-level Meeting of the UN General Assembly, in which heads of state and government will review progress and set a new vision to prevent and control NCDs and promote mental health beyond 2030: how to expand and amplify progress at rates not only in the past decade but also those achieved early in the millennium. Our results and the experiences of countries with strong performance indicate that what is needed is investment or reinvestment in programmes that increase the coverage of efficacious diagnosis and treatment, and effective policies, such as those related to tobacco and alcohol control that are well established, or emerging ones such as those related to pricing and availability of healthy (eg, fresh fruits and vegetables) or unhealthy (eg, trans fat and sugar-sweetened beverages) foods. Crucially, these programmes should be designed to reach the people that account for the largest number of disease cases and deaths, yet are persistently and increasingly excluded from the benefits of health policies and programmes, as done for the aforementioned screening programme in South Korea, for example. At the same time, for finite health budgets to revive and exceed the progress seen around the turn of the millennium, the programmes and policies must be rigorously evaluated based on the relative size of the conditions they intend to address and their effectiveness in real-world implementation, and be amended or even discontinued when ineffective. Given the range, heterogeneity, and dynamics of NCDs, there is a need to adopt a learning health system approach,^189^ which systematically collects data on NCD interventions and outcomes, benchmarks performance across and within countries to detect and identify and learn from the reasons for differential performance,^190^ and uses explicit policy trials, natural experiments, and other evaluation techniques to provide insights on what programmes and policies work to reduce mortality and improve clinically relevant outcomes in the real world. Numerous examples presented in this work show that this is an achievable ambition for the remainder of the SDG period and for the post-SDG agenda.

## Supplementary Material

Supplementary Appendix

## Data Availability

Data on the primary outcome, the probability of dying from an NCD between birth and age 80 years in 2001, 2010, and 2019, is available from the Zenodo repository (https://doi.org/10.5281/zenodo.16875485). Data on age-specific and cause-specific mortality used to calculate the primary outcome can be requested from WHO. The computer code used for the analyses is also available from the same Zenodo repository.

## Research in context

### Evidence before this study

We searched MEDLINE (via PubMed) for articles published from database inception up to May 1, 2025, with no language restrictions, using the following search terms: (“Noncommunicable Diseases”[MAJR] OR “NCD”[Title/Abstract] OR “Non-communicable disease*”[Title/Abstract]) AND (“Mortality”[MAJR] OR “Probability of Death”[Title/Abstract] OR “Death rate*”[Title/Abstract]) AND (“Trend*”[Title/Abstract] OR “Change*”[Title/Abstract]) AND (“Global Health”[mh] OR “Country”[Title/Abstract] OR “Countries”[Title/Abstract] OR “National”[Title/Abstract] OR “Global”[Title/Abstract] OR “Population-based”[Title/Abstract]) NOT (Comment[ptyp] OR Editorial[ptyp] OR Letter[ptyp] OR Case Reports[ptyp]). We identified studies that reported changes over time in non-communicable disease (NCD) mortality using national vital registration data or multicountry and global estimates of mortality. These studies used several metrics to measure NCD mortality. Most of these studies analysed one country or focused on one NCD or a group of related NCDs, and some were only at one point in time. Few evaluated contributions of different causes of death or age groups to changes in overall NCD mortality. Two previous NCD Countdown 2030 papers reported change in NCD mortality from 2010 to 2016. One of these attributed changes in NCD mortality to broad groups of causes of deaths (eg, all circulatory diseases and all cancers). The other used simulation analysis to calculate how much NCD mortality would decline if deaths from different conditions changed at the same rate as in well performing countries. The primary outcome of these analyses excluded deaths outside the age range of 30–70 years or from causes other than cardiovascular diseases, cancers, diabetes, and chronic respiratory diseases. Some papers from the Global Burden of Diseases, Injuries and Risk Factors Study reported mortality for NCD causes of death alongside other (non-NCD) conditions. We did not identify any global study that systematically compared recent trends in NCD mortality with those in preceding decades or benchmarked country-level changes against comparator countries, especially with attribution to specific NCD causes of death.

### Added value of this study

To our knowledge, this is the first analysis not only to report change in NCD mortality at the national level across time for all countries but also to benchmark national performance against each country’s own historical performance and against regional best performers. We systematically attributed change in overall NCD mortality, and its variations between time periods and countries, to specific causes of death and age groups.

### Implications of all the available evidence

NCD mortality has improved for most countries, with heterogeneous magnitudes of change across the world. Many countries showed a slowdown or reversal of progress earlier in the millennium. Performance in reducing NCD mortality was rarely dominated by one NCD cause of death, and often resulted from a combination of changes in multiple diseases. Many NCDs with well established interventions and with large declines in the first decade of the millennium saw a slowdown or reversal of their decline after 2010. These patterns point towards underinvestment in and underutilisation of effective interventions that were rolled out late in the 20th century, or towards these interventions not reaching people most in need.

## Panel 1 Key findings

- NCD mortality declined in approximately 80% of the world’s countries, where more than 70% of the world population resided, from 2010 to 2019
- In approximately 60% of countries, the decline from 2010 to 2019 was smaller than it had been in the preceding decade or there was a reversal of the earlier decline
- Within all regions, there were substantial performance gaps between the regional frontrunner and other countries in terms of how much NCD mortality declined from 2010 to 2019
- National performance in reducing NCD mortality from 2010 to 2019 was rarely dominated by one NCD and often resulted from a combination of changes in multiple NCDs
- In some countries, NCD mortality in working and older ages changed in the same direction, leading to large overall declines or increases; in others, it changed in opposite directions, diminishing the magnitude of the overall change

## Panel 2 Country case studies*

### Varying performance in high-income western countries

Females and males in the USA had the smallest declines in the probability of dying from a non-communicable disease (NCD) before age 80 years from 2010 to 2019 of any high-income western country; those in Germany had the second smallest for females and third smallest for males. These countries’ poor performance was a consequence of having had some of the largest slowdowns in NCD mortality decline compared with the first decade of the millennium, reaching near-stagnation in the case of males in the USA.

In the USA, NCD mortality declined in most age groups from 2010 to 2019 but increased in ages 20–45 years, a rare phenomenon in high-income western countries. For almost all age groups, regardless of whether mortality decreased or increased from 2010 to 2019, the change was a deterioration compared with the previous decade. The combination of small reductions in mortality in older ages and its stagnation or rise in young adults and working ages underlies the USA’s particularly poor performance among high-income western countries.

Epidemiologically, the poor performance of the USA from 2010 to 2019 can be summarised as a rise in the probability of dying from neuropsychiatric conditions, which was not offset by the continued declines in cancers and circulatory diseases. Specifically, although mortality from most cancers, ischaemic heart disease, and chronic obstructive pulmonary disease (COPD) declined from 2010 to 2019, most of these declines were smaller than the preceding decade. Lung cancer and some other cancers and kidney diseases were among the few exceptions, and had larger declines from 2010 to 2019 than in the preceding decade, but did not compensate for the deterioration in changes over time for other causes of death. Mortality from some neuropsychiatric conditions (comprising Alzheimer disease and other dementias, alcohol use disorders, and the aggregate group of all other neuropsychiatric conditions [which include drug use disorders that were not analysed separately in this work]), diabetes and its kidney complications, liver cirrhosis, and liver and pancreatic cancers increased more from 2010 to 2019 than in the previous decade or deteriorated from a decrease to an increase.

In Germany, NCD mortality declined from 2010 to 2019 from adolescence up to age 80 years, except for females aged 30–40 years and 65–75 years, who had small increases. As in the USA, these changes were a deterioration compared with the previous decade in most age groups. In terms of causes of death, circulatory diseases and neuropsychiatric conditions had a similar role as in the USA, where a rise in neuropsychiatric conditions was not offset by the decline in circulatory diseases, which was smaller from 2010 to 2019 compared with the previous decade. Furthermore, changes in cancer mortality showed greater deterioration over time in Germany than in the USA; in both countries, cancers contributed to a decrease in mortality from 2010 to 2019, but less than in the previous decade. Among cancers, mortality from lung cancer, which declined in the USA, increased in German females from 2010 to 2019 to a larger extent than in most other high-income western countries.

In contrast with the USA and Germany, Norway and Sweden had two of the largest declines in the probability of dying from an NCD before age 80 years from 2010 to 2019, and there was relatively little slowdown in their mortality decline compared with the preceding decade. The strong and sustained success in Norway and Sweden arose from a few factors. In these countries, NCD mortality generally declined throughout working and older ages from 2010 to 2019. In some adolescent and working age groups, there was an acceleration of NCD mortality decline, which is in contrast to the slowdown or even reversal of progress seen in the USA and Germany. Most NCD causes of death contributed to the sustained decrease in overall NCD mortality in Norway and Sweden. There were a few exceptions, for example Alzheimer disease and other dementias, and pancreatic and liver cancers for one or both sexes. Although the decline in mortality from some causes of death was smaller than in the previous decade, the extent of slowdown in Norway and Sweden was less pronounced than in the USA and Germany, and was compensated for by the acceleration of declines in mortality from lung, colorectal, breast, cervical, prostate, and some other cancers, and from diabetes and its kidney complications.

### Progress at low levels of NCD mortality in east Asia

In 2010, Japan had the lowest probability of dying from an NCD between birth and age 80 years for both sexes (appendix pp 15–16). South Korea also had very low NCD mortality compared with high-income western countries due to a very large reduction in NCD mortality from 2001 to 2010, greater than that of all high-income western countries for both sexes. NCD mortality in Japan and South Korea continued to decline from 2010 to 2019. While the declines in NCD mortality from 2010 to 2019 in Japan and South Korea were smaller than in the preceding decade for most age groups and causes, South Korea’s reduction remained greater than that of any high-income western country. The continued decline reinforces the fact that low mortality is not an obstacle to continued progress and substantial progress is possible even at low mortality. In 2019, Japan ranked second lowest in the world for females and third lowest for males in the probability of dying from an NCD between birth and age 80 years, and South Korea ranked lowest in the world for females and fourth lowest in the world for males.

Japan and South Korea achieved reductions in mortality in every age group from adolescence up to age 80 years for both sexes, and in most causes of death from 2010 to 2019. For example, mortality from liver cancer, which increased in most high-income western countries, decreased in both Japan and South Korea, and mortality from Alzheimer disease and other dementias, which increased in most high-income western countries, decreased in South Korea. More impressively, both countries achieved a small acceleration in their declines in ischaemic heart disease mortality, contrasting with the slowdowns in high-income western countries. China’s NCD mortality was not as low as that of Japan or South Korea in 2010 but paralleled their declines from 2010 to 2019 across most age groups and causes of death including the declines for liver cancer, Alzheimer disease and other dementias, and ischaemic heart disease.

China also had the largest reduction in COPD mortality for both sexes among the countries analysed for cause-specific mortality. The magnitude of China’s NCD mortality decline was not as great as that of South Korea, the regional best performer, but greater than that of Japan. Like South Korea and Japan, Chinese females’ decline in NCD mortality was smaller from 2010 to 2019 than in the previous decade. Males in China had a slightly larger decline in NCD mortality from 2010 to 2019 than in the previous decade, which contrasts with the slowdown in male mortality decline in South Korea and Japan. The slowdown in NCD mortality decline for Chinese females was seen in 12 of the 16 5-year age groups analysed, including in all age groups over 50 years. Trends in mortality for most causes of death deteriorated for Chinese females, either slowdowns of declines compared with the preceding decade or, in the case of pancreatic cancer and lymphomas and multiple myelomas, reversal of a previous decrease to become an increase in mortality from 2010 to 2019. The exception was ischaemic heart disease, for which the decline from 2010 to 2019 for Chinese females was a reversal of the increase seen in the previous decade. For males, the decline in NCD mortality slowed from 2010 to 2019 for many young and working age adults, but accelerated for most age groups above age 60 years leading to an overall improvement. In terms of causes of death, there was acceleration of declines in mortality or reversal of previous increases among Chinese males for 11 of 20 NCD causes of death, most notably ischaemic heart disease, stroke, and lung cancer (but not COPD). These differences across ages and causes of death underlie the sex differences in directional change between the two decades.

### Rise in NCD mortality in India

The probability of dying from an NCD between birth and age 80 years increased in India from 2010 to 2019, in contrast with the decrease seen in high-income western countries and high-income countries and emerging economies in east Asia. The increase was larger for females than for males, for whom the probability of dying from an NCD between birth and age 80 years increased by only 0·1 percentage points. The increase in the probability of dying from an NCD between birth and age 80 years was driven by an increase in mortality above age 40 years for females and above age 55 years for males. Mortality from most causes of death increased from 2010 to 2019, with particularly large contributions to an increase in overall NCD mortality from ischaemic heart disease and diabetes (including chronic kidney disease due to diabetes) for both sexes. There were decreases in mortality from cirrhosis of the liver and the residual category of all other NCDs for both sexes, with additional improvements for males in stomach cancer, COPD, stroke, and the residual category of all other circulatory diseases.

The 2010–19 change was a deterioration (ie, going from a decrease to an increase) compared with the preceding decade for females, but an improvement (ie, smaller increase) compared with the preceding decade for males. For females, this occurred as a result of deterioration in the direction or size of change for all causes of death except cirrhosis of the liver, COPD, and the residual category of all other NCDs. For males, it was a result of an improvement in the direction or size of change for eight of the 20 NCD causes of death analysed, including COPD, ischaemic heart disease, and cirrhosis of the liver, countered by less favourable (but smaller) changes in some other causes of death (eg, upper aerodigestive tract cancers and stroke). These results are subject to substantial uncertainty because mortality data are limited with quality evaluated as very low.

### Highly variable progress in Latin America and the Caribbean

Countries in Latin America and the Caribbean had an important role in mobilising political support for the first UN High-Level Meeting on NCDs. Despite this early awareness, countries in this region had more variable progress in NCD mortality than in other regions. Of the Latin American and Caribbean countries included in the analysis of causes of death, Chile had the largest reduction in NCD mortality from 2010 to 2019 for males and the second largest for females. In 2019, the population of Chile had a lower probability of dying from an NCD before age 80 years than the USA and some other high-income western countries. This success followed declines in NCD mortality in older as well as working ages, especially for males. Similarly, mortality declined for most NCD causes of death for both sexes, although there were exceptions, mainly cancers (eg, increases in mortality from colorectal and liver cancers for both sexes and from lung cancer for females). Chile’s impressive NCD decline from 2010 to 2019 was an improvement compared with 2001 to 2010, in a region where many countries had a deterioration in the direction or size of change, particularly for males. Its improvement was driven by acceleration of mortality declines in older age groups, which compensated for slowdowns in the declines in working ages, particularly among those aged 55–60 years, and in some child and adolescent age groups. In terms of causes of death, both sexes saw an acceleration in declines or a reversal of previous increases for stroke, COPD, some cancers, diabetes and related chronic kidney disease, and kidney diseases, although the direction or size of change deteriorated for some other causes of death such as ischaemic heart disease for both sexes, and cervical, colorectal, and lung cancers for females.

Unlike Chile, Jamaica and Peru had increases in NCD mortality from 2010 to 2019. These increases resulted from a rise in mortality from most NCD causes of death, including most cardiometabolic and renal diseases and many cancers, in both countries for males and in Peru for females. Both countries had experienced a decline in NCD mortality from 2001 to 2010, and saw a reversal from 2010 to 2019, with a particularly large swing from reduction to increase among males in Jamaica. This was the result of a deterioration of trends (ie, larger increase, smaller decline, or a reversal from a decline to an increase) for most NCD causes of death from the first to the second decade of the millennium.

* Countries in case studies are selected from those used for analysis of specific NCD causes of death in figures 7–9.
