# Supplementary material for: Benchmarking progress in non-communicable diseases: a global analysis of cause-specific mortality from 2001 to 2019
Source: Lancet. Author manuscript; Available in PMC 2025 Oct 11. (PMC7618237; doi:10.1016/S0140-6736(25)01388-1)
Supplement: Supplementary Appendix [file EMS208686-supplement-Supplementary_Appendix.pdf]

THE LANCET

Supplementary appendix

This appendix formed part of the original submission and has been peer reviewed. We post it as supplied by the authors.

Supplement to: NCD Countdown 2030 Collaborators. Benchmarking progress in non-communicable diseases: a global analysis of cause-specific mortality from 2001 to 2019. *Lancet* 2025; published online Sept 10. https://doi.org/10.1016/S0140-6736(25)01388-1.

Table of Contents

Appendix Text 1. Methods. 3

Appendix Text 2. NCD mortality during and following the COVID-19 pandemic. 7

Appendix Text 3. Comparison with other studies. 8

Appendix Table 1. List of analysis regions and countries and territories in each region by data quality. 9

Appendix Table 2. NCD causes of death, and residual categories, used in the analysis of cause-specific mortality. 11

Appendix Table 3. Probability of dying from NCD4 between 30 and 70 years of age in 2001, 2010 and 2019, and change in these probabilities from 2001 to 2010 and from 2010 to 2019. 13

Appendix Figure 1. Additional probability of dying from any NCD between birth and 70 or 80 years of age compared to the probability of dying from NCD4 between 30 and 70 years of age. 15

Appendix Figure 2. Change in NCD mortality from 2010 to 2019 in relation to the level of mortality in 2010. 17

Appendix Figure 3. Change in NCD mortality from 2010 to 2019 in females and males. 19

Appendix Figure 4. Age-specific death rates from NCDs in 2001, 2010 and 2019. 21

Appendix Figure 5. Comparison of change in NCD mortality between birth and 80 years of age from 2010 to 2019 with change from 2001 to 2010. 24

Appendix Figure 6. Percentage point contributions of mortality from different NCD causes of death and in different age groups to overall change in NCD mortality from 2010 to 2019. 26

Appendix Figure 7. Percentage point contributions of mortality from different NCD causes of death and in different age groups to slowdown or acceleration of change in NCD mortality from 2010 to 2019 compared to change from 2001 to 2010. 29

Appendix Figure 8. Percentage point contributions of mortality from different NCD causes of death and in different age groups to how much NCD mortality in each country lags its regional benchmark. 32

Appendix Figure 9. Contributions of mortality from aggregated NCD causes of death to overall change in NCD mortality from 2010 to 2019. 35

Appendix Figure 10. Contributions of mortality from aggregated NCD causes of death to slowdown or acceleration of change in NCD mortality from 2010 to 2019 compared to change from 2001 to 2010. 37

Appendix Figure 11. Contributions of mortality from aggregated NCD causes of death to how much NCD mortality in each country lags its regional benchmark. 39

Appendix Figure 12. Probability of dying around and after the COVID-19 pandemic. 41

Appendix Figure 13. Probability of dying from an NCD between birth and 80 years of age in 2001 with uncertainty intervals. 44

Appendix Figure 14. Probability of dying from an NCD between birth and 80 years of age in 2010 with uncertainty intervals. 46

Appendix Figure 15. Probability of dying from an NCD between birth and 80 years of age in 2019 with uncertainty intervals. 48

Appendix Figure 16. Change in the probability of dying from an NCD between birth and 80 years of age from 2001 to 2010 with uncertainty intervals. 50

Appendix Figure 17. Change in the probability of dying from an NCD between birth and 80 years of age from 2010 to 2019 with uncertainty intervals. 52

Appendix Figure 18. Probability of dying from NCD4 between 30 and 70 years of age in 2019 and change from 2010 to 2019. 54

Appendix Figure 19. Change in NCD4 mortality from 2001 to 2019. 56

Appendix Figure 20. Contributions of mortality from different NCD4 causes of death and in different age groups to overall change in NCD mortality from 2010 to 2019. 58

Appendix Figure 21. Contributions of mortality from different NCD4 causes of death and in different age groups to slowdown or acceleration of change in NCD4 mortality from 2010 to 2019 compared to change from 2001 to 2010. 61

Appendix Figure 22. Contributions of mortality from different NCD4 causes of death and in different age groups to how much NCD4 mortality in each country lags its regional benchmark. 64

Appendix Figure 23. Summary of how direction and size of change in NCD mortality differs when NCD4 between 30 and 70 years is used in place of all NCDs between birth and 80 years. 67

Appendix Figure 24. Change in NCD4 mortality from 2010 to 2019 in relation to the level of mortality in 2010. 69

Appendix Figure 25. Change in NCD4 mortality from 2010 to 2019 in females and males. 71

Appendix Figure 26. Age-specific death rates from NCD4 in 2001, 2010 and 2019. 73

Appendix Figure 27. Comparison of change in NCD4 mortality between 30 and 70 years of age from 2010 to 2019 with change from 2001 to 2010. 76

References 78

Appendix Text 1. Methods.

Analysis period

Our analysis covered the years 2001 to 2019. We restricted the analysis up to and including the year 2019 because the COVID-19 pandemic, and the responses to it, affected NCD mortality in 2020 and 2021. This may have happened because mortality from some NCDs increased due to interruptions in care, whereas those from others may have decreased because some people who may have died from an NCD died from COVID-19 or the assignment of cause of death changed.[1-3] Changes in risk factors like air pollution[4] and alcohol use[5] may have also led to decreases or increases in NCDs, depending on how they changed in each country. As a result, global analyses have used classifications such as "other pandemic-related mortality"[6,7] to report some deaths in 2020 and 2021 that did not readily map to either COVID-19 or other underlying causes of death. Few countries have released their vital registration data from 2022 and 2023 to allow analysis of post-pandemic NCD mortality. We present examples of NCD mortality up to 2023 for countries with high-quality medical certification of deaths (Appendix Text 2).

Primary outcome

As stated in the main paper, we used the probability of dying from an NCD between birth and 80 years of age in the absence of competing causes of death to measure NCD mortality; this is referred to as "unconditional probability". The age range of birth to 80 years of age is broader than the range used in SDG target 3.4 (between 30 and 70 years), and we consider all NCDs, whereas the SDG target 3.4 is restricted to cancers, cardiovascular diseases, chronic respiratory diseases and diabetes (referred to as NCD4 hereafter). We took an inclusive approach to age and disease than the SDG target 3.4 because NCD deaths in the age ranges (including both younger and older ages) and conditions not included in the SDG target are responsible for a larger share of total NCD mortality in low-income and middle-income countries (LMICS) than in high-income countries (HICs) (Appendix Figure 1).[8] By leaving out a larger share of NCD mortality in LMICs than HICs, the SDG target 3.4 indicator does not fully capture

global inequalities in NCDs.[8] Furthermore, some diseases excluded from SDG target 3.4 have shared aetiologies and interventions with those included – for example, hypertension and diabetes increase the risk of cardiovascular diseases (CVDs), which is included in SDG target 3.4 indicator, as well as chronic kidney disease (CKD) and dementia,[9-11] which are excluded from the indicator. Similarly, liver cirrhosis, excluded from the SDG target 3.4 indicator, has shared causes and interventions with liver cancer (which is included), e.g., hepatitis infection and hepatitis B virus (HBV) vaccination, and alcohol use and its control. For comparison, we also provide results for the SDG target 3.4 indicator, i.e., probability of dying from NCD4 between 30 and 70 years of age. Data on the inclusive indicator can also provide information that can guide the choice of an NCD target following the SDGs.

We restricted the age range to 80 years because, in the absence of competing causes, the probability of death becomes 100% when the entire life-course is considered, i.e., although death can be postponed, it cannot be avoided. As a result, an open-ended probability of death across the entire life-course would make it impossible to differentiate the extent of change in mortality between countries or time periods. Furthermore, compared to younger ages NCD mortality above 80 years of age is associated with a greater potential for two types of reporting errors: First, over- or under-reporting of age is more common in older than younger ages both in censuses and on the death certificated.[12-14] This leads to larger errors in age-specific population and death counts, and therefore death rates, in older ages. Second, the assignment of cause of death in older ages is more difficult, often because people have multiple conditions at the time of death.[15] As a result, a larger share of deaths are assigned to improbable or ill-defined causes of death above 80 years of age.[7,16]

Data sources

As stated in the main paper we used data on deaths from NCDs by sex and age group for 185 countries and territories (referred to as countries hereafter) from the 2021 WHO Global Health Estimates.[17] We divided these countries into eight reporting regions (Appendix Table 1) using the regional assignment

of the NCD risk factor collaboration, which is based on their geography and epidemiology as relevant for NCDs.[18,19] WHO uses vital registration, including with adjustment for incompleteness, and demographic methods to estimate all-cause mortality. It then uses vital registration, including with correction for ill-defined causes and misclassification, epidemiological surveillance and models, and results from the Global Burden of Disease (GBD) Study to estimate cause-specific mortality. These methods result in unbiased estimates, with varying levels of uncertainty in age- and cause-specific death rates depending on the extent of data availability and quality. Detailed data sources and their methods are provided elsewhere.[20]

Analytical methods

We used age-specific death rates and lifetables to calculate the primary outcome, i.e., unconditional probability of dying from an NCD between birth and 80 years of age, by country and sex.[21] Lifetables convert age-specific death rates into summary measures of population health such as life expectancy and probability of death.[21] Age-specific death rates were calculated as the number of deaths from all NCDs in five-year age groups divided by the population of each five-year age group. We calculated change in the unconditional probabilities of death from 2001 to 2010 and from 2010 to 2019 as the difference between the corresponding values in the final and first year of each period so that there is no assumption about linearity of change. The correlation coefficient between change calculated as the difference between values in final and first years and change calculated by fitting a linear relationship to all ten annual values ranged 0.96-0.98 in the two periods and two sexes. We calculated the uncertainty of probability of dying from an NCD between birth and 80 years of age and its change via the following steps: we took 10,000 independent draws from the distributions of each age- and cause-specific death rate in 2001, 2010 and 2019, using a normal distribution with a mean equal to the central estimate of the death rate and standard deviation equal to the standard error of the death rate; we repeated the lifetable calculations for each set of independent draws, and used the 2.5th and 97.5th percentiles of the

10,000 resultant calculations as the 95% uncertainty intervals of the probability of dying from an NCD between birth and 80 years of age and its change.

We applied the Horiuchi method of decomposition for the analyses related to questions 1b, 2b and 3b.[22] When applied to the probability of death from all NCDs between birth and 80 years of age, the Horiuchi method uses the age- and cause-specific death rates to compute the contributions of different causes of death and age groups to changes in this probability. The Horiuchi method provides an exact decomposition, which mathematically ensures that the sum of the contributions of different causes of death and different age groups equals the total observed change. The package DemoDecomp[23] was used for decomposition.

Appendix Text 2. NCD mortality during and following the COVID-19 pandemic.

As stated in Methods the COVID-19 pandemic affected the recorded number of deaths from NCDs, because there were changes in both the actual causes of death and possibly the assignment of underlying cause of death. We used data from 17 countries from three regions (high-income western, central and eastern Europe, and east and southeast Asia) with high-quality data (as defined in Methods) on cause specific mortality through 2022 or 2023 to visualise trends in the probability of dying from NCDs since the beginning of the pandemic.

The probability of dying from communicable, maternal, perinatal and nutritional conditions increased substantially in most of these countries during the pandemic and has not yet fully returned to its pre-pandemic baseline (Appendix Figure 12). Among NCDs, the decline in cancer mortality did not change due to the pandemic COVID-19. There were some increases in the probability of dying from cardiovascular diseases during the pandemic, especially in central and eastern European countries. The impacts of the pandemic on other NCDs were more varied across countries, and no generalisations could be made based on data from these countries.

Appendix Text 3. Comparison with other studies.

Prior papers by NCD Countdown 2030[8,24] also reported variable change in NCD mortality across countries, as have studies that considered specific NCD causes of death. However, most of these studies did not analyse decadal improvement or deterioration in changes over time, nor did they compare changes with regional benchmark countries. Some studies analysed the contributions of different diseases to change in life expectancy.[6,25] Life expectancy calculations takes into account NCDs as well as other conditions like injuries, infectious diseases and maternal conditions, whereas probability of dying from an NCD does not use death rates from non-NCD causes of death. There are also some differences between life expectancy and probability of dying in how they are affected by age-specific death rates. Despite these differences, the life expectancy studies also found a slowdown in life expectancy increase in many high-income western countries, with the same NCD cause of death as we found playing predominant roles.

In terms of specific NCDs, a study on CVD mortality[26] found a general slowdown in the decline in high-income countries, which is consistent with our results. Our results are consistent with studies which found that overall cancer mortality (i.e., aggregated across all cancers and ages) has declined in high-income countries,[27-29] as well as with those on site-specific cancers that found rises in mortality (e.g., liver and pancreatic cancer), continued or accelerated declines (e.g., lung cancer in men) or a slow-down of declines (e.g., stomach, prostate, breast and cervical cancer).[27-37] We found that dementia mortality contributed to an increase in NCD mortality, especially in high-income western countries. This finding, which is largely distinct from the role played by population aging (as the probability of dying from an NCD is only dependent on age-specific mortality), has also been observed in other studies.[38] The increase is, however, inconsistent with the evidence on the stable or declining trends in dementia incidence and prevalence,[39-42] and may be due to changes in cause-of-death assignment practices with increasing awareness and diagnosis of dementia.[38,43]

Appendix Table 1. List of analysis regions and countries and territories in each region by data quality.

Region	Data quality[a]	Countries and territories
**Central and eastern Europe**	Low	Albania, Montenegro
	Medium	Bulgaria, Bosnia and Herzegovina, North Macedonia, Poland, Russia[b], Serbia, Ukraine[b]
	High	Belarus, Czechia, Estonia, Croatia, Hungary, Lithuania, Latvia, Moldova, Romania, Slovakia, Slovenia
**Central Asia, Middle East and north Africa**	Very low	United Arab Emirates, Azerbaijan, Algeria, Libya, Morocco, Oman, Qatar, Saudi Arabia, Turkmenistan, Yemen
	Low	Bahrain, Egypt, Iraq, Kuwait, Lebanon, Palestine, Syrian Arab Republic, Tajikistan, Tunisia
	Medium	Georgia, Iran, Jordan, Türkiye, Uzbekistan
	High	Armenia, Kazakhstan, Kyrgyzstan, Mongolia
**East and southeast Asia**	Very low	China[b], Indonesia, Cambodia, Lao PDR, Myanmar, North Korea, Timor-Leste, Taiwan[b], Viet Nam
	Low	Malaysia, Thailand
	Medium	Philippines
	High	Brunei Darussalam, Japan, South Korea, Singapore
**High-income western**	Medium	France, Greece
	High	Australia, Austria, Belgium, Canada, Switzerland, Cyprus, Germany, Denmark, Spain, Finland, United Kingdom, Ireland, Iceland, Israel, Italy, Luxembourg, Malta, Netherlands, Norway, New Zealand, Portugal, Sweden, United States of America
**Latin America and the Caribbean**	Very low	Bolivia, Honduras, Haiti
	Low	Barbados, Dominican Republic, El Salvador, Trinidad and Tobago
	Medium	Argentina, Antigua and Barbuda, Bahamas, Ecuador, Grenada, Guyana, Peru, Paraguay, Suriname, Uruguay
	High	Belize, Brazil, Chile, Colombia, Costa Rica, Cuba, Guatemala, Jamaica, Saint Lucia, Mexico, Nicaragua, Panama, Puerto Rico, Saint Vincent and the Grenadines, Venezuela
**Pacific Island nations**	Very low	Papua New Guinea, Tonga, Vanuatu, Samoa
	Low	Fiji, Federated States of Micronesia, Kiribati, Solomon Islands
**South Asia**	Very low	Afghanistan, Bangladesh, Bhutan, India, Nepal, Pakistan
	Medium	Sri Lanka
**Sub-Saharan Africa**	Very low	Angola, Burundi, Benin, Burkina Faso, Botswana, Central African Republic, Cote d'Ivoire, Cameroon, DR Congo, Congo, Comoros, Djibouti, Eritrea, Ethiopia, Gabon, Ghana, Guinea, Gambia, Guinea Bissau, Equatorial Guinea, Kenya, Liberia, Lesotho, Madagascar, Mali, Mozambique, Mauritania, Malawi, Namibia, Niger, Nigeria, Rwanda, Sudan, Senegal, Sierra Leone, Somalia, South Sudan, Sao Tome and Principe, Eswatini, Chad, Togo, Tanzania, Uganda, Zambia, Zimbabwe
	Low	Cabo Verde
	Medium	Seychelles, South Africa
	High	Mauritius

[a] Data were considered high quality if a country reported at least five years of data to WHO since 2010, reported the latest year of data by ICD-10 code, and had an average usability index (defined as product of completeness of death registration and share of deaths not assigned to implausible and ill-defined codes) since 2010 of at least 80%. Data were considered medium quality if a country reported

at least five years of data to WHO since 2010, and either reported the latest year of data by ICD-10 code and had an average usability index since 2010 of between 60% and 80%, or if it reported with a summary cause list and had an average usability index of at least 80%. Countries with low-quality data were those reporting at least one year of data since 2010, either by ICD-10 code with an average usability index of 40% or greater, or with a summary cause list with an average usability index of at least 60%. Countries with no death registration or with death registration with lower completeness and cause-of-death assignment quality than the other categories were assigned to the very low-quality category.

[b] For China, Russia, Taiwan and Ukraine, the estimates incorporate information from the Global Burden of Disease Study, which are based on mortality data beyond what is reported to the WHO, resulting in estimates that reflect a higher data quality than their classification as listed above. Therefore, as stated in Methods, these four countries were included with countries with high-quality data.

Appendix Table 2. NCD causes of death, and residual categories, used in the analysis of cause-specific mortality.

Female			Male		
**Rank**	**Cause name**	**Numbers of deaths[a]**	**Rank**	**Cause name**	**Numbers of deaths[a]**
1	Ischaemic heart disease	8,394,820	1	Ischaemic heart disease	14,090,288
2	Stroke	8,145,356	2	Stroke	11,889,920
3	Trachea, bronchus, lung cancers	3,133,419	3	Trachea, bronchus, lung cancers	6,900,081
4	Chronic obstructive pulmonary disease	3,094,927	4	Chronic obstructive pulmonary disease	4,980,756
5	Breast cancer	2,329,870	5	Upper aerodigestive tract cancers[c] (mouth and oropharynx cancers, oesophagus cancer, larynx cancer)	3,466,920
6	Diabetes including chronic kidney disease due to diabetes[b]	2,247,334	6	Stomach cancer	3,381,907
7	Colon and rectum cancers	1,627,595	7	Cirrhosis of the liver	2,732,670
8	Stomach cancer	1,370,981	8	Colon and rectum cancers	2,525,505
9	Alzheimer disease and other dementias	1,300,633	9	Diabetes including chronic kidney disease due to diabetes[b]	2,449,346
10	Cirrhosis of the liver	1,112,890	10	Liver cancer	1,804,909
11	Pancreas cancer	925,382	11	Pancreas cancer	1,296,496
12	Cervix uteri cancer	905,765	12	Prostate cancer	1,077,222
13	Ovary cancer	725,992	13	Alzheimer disease and other dementias	968,275
14	Liver cancer	715,057	14	Lymphomas, multiple myeloma	882,393
15	Kidney diseases[b]	657,520	15	Kidney diseases[b]	850,337
16	Lymphomas, multiple myeloma	580,837	16	Alcohol use disorders	796,777
	All other malignant neoplasms (upper aerodigestive tract cancers, melanoma and other skin cancers[c], corpus uteri cancer, kidney cancer, bladder cancer, brain and nervous system cancers, gallbladder and biliary tract cancer, thyroid cancer, mesothelioma, leukaemia, other malignant neoplasms)	3,942,050		All other malignant neoplasms (melanoma and other skin cancers, breast cancer, testicular cancer, kidney cancer, bladder cancer, brain and nervous system cancers, gallbladder and biliary tract cancer, thyroid cancer, mesothelioma, leukaemia, other malignant neoplasms)	4,208,607
	All other circulatory diseases[e] (rheumatic heart disease, hypertensive heart disease, cardiomyopathy, myocarditis, endocarditis, other circulatory diseases)	2,924,145		All other circulatory diseases[e] (rheumatic heart disease, hypertensive heart disease, cardiomyopathy, myocarditis, endocarditis, other circulatory diseases)	4,132,107
	All other neuropsychiatric conditions[d]	1,251,963		All other neuropsychiatric conditions[d]	1,801,318
	All other NCDs	3,453,582		All other NCDs	4,508,243
	All NCDs	48,840,119		All NCDs	74,744,079

[a] Total number of deaths before 80 years of age from 2010 to 2019 in 47 countries with high-quality data and 2019 population >2 million, China, Russia, Taiwan and Ukraine.

[b] Chronic kidney disease (CKD) due to diabetes was included in diabetes and excluded from kidney diseases.

[c] Upper aerodigestive tract cancers were grouped together due to shared aetiology and frequent joint attribution in mortality coding.

[d] Neuropsychiatric conditions were treated as a single category due to overlapping symptoms and frequent comorbidity. This category included depressive disorders, bipolar disorder, anxiety disorders, schizophrenia, eating disorders, epilepsy, Parkinson disease and multiple sclerosis.[44]

[e] Hypertensive heart disease was included with all other circulatory diseases, despite having a sufficiently large number of deaths to appear separately as one of the top 16 causes of death. We did this because heart failure is the most common sequala of many of these conditions, and one that is often diagnosed in a clinical setting. Heart failure deaths are redistributed to underlying causes.[45] At the same time, hypertension is a risk factor for ischaemic heart disease (IHD) and can be misattributed as an underlying cause. These factors make the relative sizes of deaths from hypertensive heart disease and other diseases in the category of “all other circulatory diseases” unreliable relative to the combined group, even in a setting with vital registration and medical attribution of causes of death.

Appendix Table 3. Probability of dying from NCD4 between 30 and 70 years of age in 2001, 2010 and 2019, and change in these probabilities from 2001 to 2010 and from 2010 to 2019.

Estimates are shown for 185 countries and territories in eight regions, with country names coloured by region. Countries are divided into six categories of trends observed across two timeframes (from 2001 to 2010 and from 2010 to 2019). Within each of the six categories, countries are ordered by percentage point change from 2010 to 2019 (i.e. the first country listed in each category had the largest decrease or smallest increase from 2010 to 2019 within its category).

Female							Male						
	Data quality	Probability of dying from NCD4 between age 30 and 70 years (percent)			Change in probability of dying from NCD4 between age 30 and 70 years (percentage points)			Data quality	Probability of dying from NCD4 between age 30 and 70 years (percent)			Change in probability of dying from NCD4 between age 30 and 70 years (percentage points)	
		2001	2010	2019	2001-10	2010-19			2001	2010	2019	2001-10	2010-19
**Faster decline from 2010 to 2019 compared with from 2001 to 2010**													
Uzbekistan	Medium	36	31	21	-5	-10	Uzbekistan	Medium	49	42	30	-7	-12
Mongolia	High	31	26	18	-6	-8	Azerbaijan	Very low	37	32	23	-5	-10
Moldova	High	26	22	15	-4	-7	Kazakhstan	High	47	41	31	-7	-9
Azerbaijan	Very low	24	19	13	-5	-6	Russia	High	50	42	33	-7	-9
Oman	Very low	22	20	15	-2	-6	Moldova	High	41	40	32	-1	-8
Kazakhstan	High	25	20	15	-5	-6	Belarus	High	46	42	34	-4	-8
Georgia	Medium	17	17	12	-0.1	-5	Tajikistan	Low	29	27	19	-2	-8
Tajikistan	Low	25	22	17	-3	-5	Oman	Very low	31	28	21	-2	-7
Russia	High	23	18	13	-5	-5	Latvia	High	39	36	29	-4	-7
Belarus	High	21	17	12	-4	-5	Kyrgyzstan	High	38	35	29	-3	-6
Palestine	Low	19	18	13	-1	-4	Botswana	Very low	31	27	20	-4	-6
Armenia	High	19	15	11	-3	-4	North Macedonia	Medium	29	27	21	-3	-6
Kuwait	Low	11	9	5	-2	-4	Lithuania	High	33	33	27	-0.4	-6
Ukraine	High	21	18	14	-3	-4	Mongolia	High	46	44	38	-2	-5
Latvia	High	17	15	11	-3	-3	Jordan	Medium	21	17	12	-4	-5
Ghana	Very low	23	22	18	-1	-3	Palestine	Low	28	25	20	-3	-4
Guyana	Medium	28	27	23	-2	-3	Belgium	High	18	14	10	-3	-4
Iraq	Low	22	19	17	-2	-2	China	High	27	23	20	-4	-4
Indonesia	Very low	24	23	20	-1	-2	Sri Lanka	Medium	25	22	18	-3	-4
Lithuania	High	14	13	10	-0.9	-2	Hungary	High	35	32	28	-3	-4
Luxembourg	High	9	8	6	-2	-2	Armenia	High	33	31	28	-2	-4
DR Congo	Very low	24	24	22	-0.3	-2	Serbia	Medium	32	29	25	-3	-4
Norway	High	9	8	6	-2	-2	Finland	High	17	15	11	-2	-4
Turkiye	Medium	13	12	10	-2	-2	Bangladesh	Very low	27	24	20	-3	-4
Chile	High	10	9	7	-1	-2	Chile	High	15	14	11	-2	-3
Guinea Bissau	Very low	26	24	22	-1	-2	Kuwait	Low	15	13	11	-1	-2
Belgium	High	9	8	7	-0.7	-2	Iraq	Low	29	27	24	-2	-2
Bangladesh	Very low	16	16	14	-0.6	-2	Bulgaria	Medium	35	33	31	-2	-2
Kiribati	Low	35	35	34	-0.1	-1	Pakistan	Very low	30	28	26	-2	-2
Mexico	High	15	14	12	-0.9	-1	Kiribati	Low	51	50	48	-1	-2
Togo	Very low	24	23	21	-1	-1	Lebanon	Low	16	15	13	-1	-2
Eritrea	Very low	26	25	23	-1	-1	Chad	Very low	28	26	24	-2	-2
Bahamas	Medium	17	17	15	-0.3	-1	Burkina Faso	Very low	28	27	25	-0.5	-2
Sri Lanka	Medium	13	12	10	-1	-1	Madagascar	Very low	29	28	26	-1	-2
Tonga	Very low	24	23	22	-0.9	-1	Bahamas	Medium	25	24	22	-1	-2
Benin	Very low	19	19	18	-0.4	-1	Tonga	Very low	32	31	29	-1	-2
Finland	High	8	7	6	-1	-1	Costa Rica	High	13	12	10	-1	-1
Burkina Faso	Very low	21	20	19	-0.8	-1	Benin	Very low	25	24	23	-1	-1
Morocco	Very low	22	21	20	-0.6	-1	Guyana	Medium	32	31	30	-0.9	-1
Cameroon	Very low	23	22	21	-0.6	-1	Antigua and Barbuda	Medium	20	19	18	-0.7	-0.8
Nicaragua	High	12	12	12	-0.0	-0.8	Nicaragua	High	13	13	13	-0.2	-0.4
Timor-Leste	Very low	20	20	19	-0.6	-0.8							
Panama	High	9	9	8	-0.4	-0.6							
Nepal	Very low	18	18	18	-0.1	-0.1							
**Slower decline from 2010 to 2019 compared with from 2001 to 2010**													
Qatar	Very low	23	17	12	-6	-5	Qatar	Very low	29	19	12	-9	-7
Jordan	Medium	23	14	9	-8	-5	Estonia	High	37	27	21	-10	-6
Kyrgyzstan	High	25	19	14	-6	-5	Slovakia	High	33	27	21	-6	-6
Congo	Very low	34	27	23	-7	-4	Bahrain	Low	28	19	14	-9	-6
Myanmar	Very low	29	24	20	-5	-4	South Korea	High	21	14	9	-7	-5
Bahrain	Low	27	17	13	-10	-4	Afghanistan	Very low	45	38	33	-8	-5
Estonia	High	16	11	8	-5	-3	Czechia	High	26	22	17	-5	-5
Serbia	Medium	20	16	14	-4	-3	Poland	Medium	31	26	22	-5	-4
China	High	18	13	11	-5	-2	Eritrea	Very low	37	33	29	-4	-4
Denmark	High	13	10	8	-4	-2	Ukraine	High	43	38	35	-4	-4
Slovakia	High	14	12	9	-3	-2	Singapore	High	20	13	10	-6	-3
Haiti	Very low	38	35	33	-2	-2	Norway	High	15	12	8	-4	-3
Burundi	Very low	31	23	21	-8	-2	Netherlands	High	18	13	10	-5	-3
South Korea	High	9	6	4	-4	-2	Slovenia	High	23	16	13	-7	-3
Romania	High	18	14	12	-4	-2	Luxembourg	High	17	12	9	-5	-3
Afghanistan	Very low	39	34	33	-4	-2	Croatia	High	28	23	20	-5	-3
Czechia	High	13	10	8	-3	-2	Austria	High	18	14	12	-3	-3
Lao PDR	Very low	28	23	22	-5	-2	France	Medium	17	14	11	-3	-3
Brazil	High	15	13	11	-3	-2	Argentina	Medium	24	20	17	-4	-3
Ethiopia	Very low	22	17	15	-6	-2	Uruguay	Medium	25	21	19	-3	-3
Colombia	High	14	10	8	-4	-2	Brazil	High	23	19	17	-4	-3
Lebanon	Low	12	9	8	-3	-2	Lao PDR	Very low	36	32	29	-5	-2
Sweden	High	9	7	6	-2	-2	Uganda	Very low	32	25	23	-7	-2
Montenegro	Low	17	14	13	-3	-2	Switzerland	High	14	11	8	-3	-2
Bolivia	Very low	18	17	15	-2	-2	Malta	High	17	13	11	-4	-2
Saudi Arabia	Very low	18	14	12	-4	-2	Sweden	High	14	11	8	-3	-2
Ecuador	Medium	13	11	9	-2	-2	Romania	High	33	30	27	-3	-2
North Macedonia	Medium	18	16	14	-2	-1	Fiji	Low	48	45	42	-3	-2
Poland	Medium	14	12	10	-2	-1	Spain	High	17	14	11	-3	-2
Thailand	Low	13	11	9	-2	-1	Congo	Very low	34	24	22	-9	-2
Gabon	Very low	21	17	16	-4	-1	Mauritius	High	35	29	27	-5	-2
Netherlands	High	11	9	7	-2	-1	Denmark	High	19	14	11	-5	-2
Singapore	High	11	7	5	-4	-1	Turkiye	Medium	26	22	20	-4	-2
Ireland	High	11	8	7	-3	-1	Italy	High	16	12	10	-4	-2
Brunei Darussalam	High	16	14	12	-3	-1	Somalia	Very low	35	33	30	-3	-2
Namibia	Very low	20	18	16	-2	-1	Ireland	High	19	12	10	-7	-2
Croatia	High	13	10	9	-3	-1	Belize	High	22	18	16	-4	-2
Comoros	Very low	24	20	18	-4	-1	Guinea Bissau	Very low	37	30	28	-7	-2
Puerto Rico	High	11	8	7	-2	-1	UK	High	18	13	11	-4	-2
Trinidad and Tobago	Low	22	16	15	-5	-1	Japan	High	14	12	10	-2	-2
Pakistan	Very low	26	24	23	-2	-1	Turkmenistan	Very low	38	32	30	-5	-2
Algeria	Very low	14	13	11	-2	-1	Cyprus	High	15	12	10	-3	-2
New Zealand	High	12	8	7	-3	-1	Israel	High	15	11	9	-4	-2
Bosnia and Herzegovina	Medium	15	13	12	-2	-1	Germany	High	18	15	13	-4	-2
Taiwan	High	11	8	7	-3	-1	Canada	High	15	12	10	-4	-2
Bhutan	Very low	19	15	14	-4	-1	Nigeria	Very low	22	18	17	-3	-2
Canada	High	10	8	7	-2	-1	Ethiopia	Very low	21	17	15	-5	-2
Tunisia	Low	13	11	10	-2	-1	Bhutan	Very low	21	18	16	-3	-1
Bulgaria	Medium	18	16	14	-3	-1	Myanmar	Very low	34	30	29	-3	-1
Cambodia	Very low	23	19	18	-4	-1	Saudi Arabia	Very low	18	15	14	-3	-1
Madagascar	Very low	27	25	24	-1	-1	New Zealand	High	16	12	10	-5	-1
Rwanda	Very low	30	18	17	-12	-1	Ecuador	Medium	15	13	12	-2	-1
Malta	High	10	8	6	-3	-1	USA	High	19	16	15	-3	-1
UK	High	11	8	7	-3	-1	Portugal	High	19	15	14	-4	-1
Yemen	Very low	26	23	22	-3	-1	Cambodia	Very low	30	28	26	-2	-1
Mali	Very low	26	24	23	-2	-1	Namibia	Very low	28	25	24	-2	-1
USA	High	13	10	9	-3	-1	Tunisia	Low	20	18	17	-2	-1
Mauritius	High	21	18	17	-4	-1	Puerto Rico	High	19	14	13	-4	-1
Hungary	High	16	15	14	-2	-1	Iceland	High	13	10	9	-3	-1
Belize	High	19	16	15	-3	-0.9	Algeria	Very low	17	14	13	-3	-1
Federated States of Micronesia	Low	35	34	33	-1	-0.9	Taiwan	High	20	17	16	-3	-1
Portugal	High	9	7	6	-3	-0.9	Sudan	Very low	26	23	22	-3	-1
Slovenia	High	11	7	7	-3	-0.9	Federated States of Micronesia	Low	46	45	44	-1	-1
Nigeria	Very low	18	16	15	-2	-0.9	Colombia	High	16	11	10	-4	-0.9
Switzerland	High	7	6	5	-1	-0.9	Bolivia	Very low	18	15	14	-2	-0.9
Costa Rica	High	10	8	7	-2	-0.8	Senegal	Very low	25	23	22	-2	-0.9
Israel	High	10	7	6	-3	-0.8	Saint Lucia	High	23	20	19	-3	-0.8
Somalia	Very low	27	25	24	-2	-0.8	Panama	High	14	13	12	-1	-0.8
Cuba	High	15	13	12	-2	-0.8	Rwanda	Very low	37	22	21	-15	-0.8
Cyprus	High	8	6	5	-2	-0.7	Greece	Medium	18	16	15	-2	-0.8
Austria	High	9	7	7	-2	-0.7	Australia	High	14	10	10	-3	-0.8
Greece	Medium	8	7	6	-1	-0.7	Comoros	Very low	24	20	20	-4	-0.8
Uganda	Very low	23	17	16	-6	-0.7	Iran	Medium	20	16	16	-4	-0.7
Japan	High	6	5	5	-0.9	-0.6	Montenegro	Low	29	25	25	-3	-0.7
Italy	High	8	6	6	-1	-0.6	DR Congo	Very low	28	26	26	-2	-0.7
Australia	High	8	6	6	-2	-0.6	Vanuatu	Very low	45	43	43	-2	-0.6
Argentina	Medium	13	11	11	-1	-0.6	Brunei Darussalam	High	19	17	16	-3	-0.6
Senegal	Very low	22	21	20	-1	-0.6	Yemen	Very low	32	28	28	-4	-0.6
Sudan	Very low	24	21	20	-3	-0.5	Seychelles	Medium	30	26	25	-4	-0.4
Equatorial Guinea	Very low	23	20	19	-3	-0.5	Zambia	Very low	26	24	24	-2	-0.3
Angola	Very low	25	20	20	-4	-0.5	Bosnia and Herzegovina	Medium	24	23	23	-1	-0.2
Turkmenistan	Very low	24	20	20	-4	-0.5	Gambia	Very low	26	25	25	-2	-0.2
France	Medium	7	6	6	-0.7	-0.5	Burundi	Very low	30	24	24	-7	-0.1
Saint Lucia	High	16	13	12	-3	-0.5							
Mauritania	Very low	21	19	19	-2	-0.5							
Germany	High	9	8	8	-1	-0.4							
Iceland	High	9	6	6	-3	-0.3							
Spain	High	7	6	5	-1	-0.3							
Zambia	Very low	21	18	18	-2	-0.2							
Cabo Verde	Low	13	11	11	-2	-0.2							
Malaysia	Low	17	15	15	-2	-0.1							
Sao Tome and Principe	Very low	21	19	19	-2	-0.1							
Vanuatu	Very low	30	29	29	-1	-0.1							
United Arab Emirates	Very low	11	6	6	-5	-0.1							
Uruguay	Medium	13	11	11	-2	-0.0							
Iran	Medium	14	10	10	-4	-0.0							
**Declining from 2010 to 2019 after increasing from 2001 to 2010**													
Albania	Low	9	14	5	5	-9	Georgia	Medium	33	37	30	4	-7
Eswatini	Very low	27	34	27	7	-7	Albania	Low	18	19	15	2	-4
Botswana	Very low	13	21	16	9	-5	South Africa	Medium	25	26	22	1	-4
South Africa	Medium	16	23	18	7	-5	Cameroon	Very low	29	29	26	0.3	-3
Zimbabwe	Very low	19	33	30	13	-3	Sierra Leone	Very low	26	26	23	0.1	-3
Fiji	Low	35	36	33	0.6	-3	Togo	Very low	30	31	28	0.4	-3
Egypt	Low	22	23	21	0.5	-2	Guinea	Very low	23	26	24	3	-3
North Korea	Very low	19	21	18	2	-2	North Korea	Very low	28	31	29	4	-2
Sierra Leone	Very low	23	26	24	3	-2	Egypt	Low	33	34	31	0.8	-2
Liberia	Very low	23	24	22	1	-2	Ghana	Very low	24	27	25	3	-2
Paraguay	Medium	14	14	13	0.3	-2	Cote d'Ivoire	Very low	25	26	24	0.7	-2
Guinea	Very low	24	25	23	1	-1	Liberia	Very low	20	21	20	0.7	-2
Chad	Very low	21	22	21	0.9	-1	Gabon	Very low	25	26	25	0.9	-2
Solomon Islands	Low	28	34	33	6	-1	Paraguay	Medium	17	20	18	2	-1
Viet Nam	Very low	13	13	12	0.4	-0.8	Indonesia	Very low	25	27	26	2	-1
Cote d'Ivoire	Very low	15	20	19	4	-0.8	Philippines	Medium	28	32	31	4	-1
Barbados	Low	14	15	14	0.0	-0.5	Viet Nam	Very low	26	29	28	2	-1
Kenya	Very low	13	16	16	3	-0.5	Eswatini	Very low	35	43	41	8	-1
							Solomon Islands	Low	38	43	42	5	-1
							Mexico	High	17	17	17	0.4	-0.4
							Mali	Very low	20	21	20	1	-0.3
							Timor-Leste	Very low	19	22	21	2	-0.2
**Increasing from 2010 to 2019 after declining from 2001 to 2010**													
Syrian Arab Republic	Low	20	16	17	-4	0.0	Syrian Arab Republic	Low	28	24	24	-4	0.1
Libya	Very low	18	18	18	-0.3	0.2	Thailand	Low	19	17	17	-2	0.1
El Salvador	Low	10	9	10	-0.4	0.2	Angola	Very low	31	25	25	-6	0.1
Djibouti	Very low	19	18	18	-1	0.3	Niger	Very low	19	18	18	-0.9	0.2
Papua New Guinea	Very low	29	27	28	-2	0.4	Grenada	Medium	25	23	23	-1	0.2
Gambia	Very low	21	20	21	-0.9	0.5	Trinidad and Tobago	Low	30	23	23	-7	0.3
Malawi	Very low	22	19	19	-3	0.6	Morocco	Very low	23	22	22	-1	0.3
Seychelles	Medium	16	14	15	-2	0.6	Cuba	High	19	19	19	-0.6	0.3
Tanzania	Very low	15	15	16	-0.5	0.8	Samoa	Very low	34	32	32	-2	0.5
Suriname	Medium	20	16	16	-4	0.8	Tanzania	Very low	20	17	18	-3	0.6
Niger	Very low	20	19	20	-1	1	Barbados	Low	19	17	17	-2	0.6
Central African Republic	Very low	29	25	26	-4	1	Central African Republic	Very low	39	37	37	-2	0.7
Venezuela	High	13	12	13	-1	2	Malaysia	Low	23	21	22	-2	1
India	Very low	18	18	19	-0.9	2	United Arab Emirates	Very low	10	8	9	-2	1
Jamaica	High	18	13	15	-5	2	Mauritania	Very low	16	14	15	-2	1
Saint Vincent and the Grenadines	High	20	16	19	-3	3	Papua New Guinea	Very low	29	28	29	-1	1
Antigua and Barbuda	Medium	15	14	17	-1	3	Sao Tome and Principe	Very low	20	19	20	-0.9	2
South Sudan	Very low	14	13	17	-1	4	Suriname	Medium	26	23	25	-3	2
							Libya	Very low	21	19	21	-1	2
							Djibouti	Very low	21	19	22	-1	2
							El Salvador	Low	13	12	15	-0.8	2
							Venezuela	High	19	17	20	-1	2
							Equatorial Guinea	Very low	27	18	20	-9	3
							Malawi	Very low	24	23	26	-1	4
							Saint Vincent and the Grenadines	High	23	19	23	-3	4
							South Sudan	Very low	22	19	24	-3	5
							Cabo Verde	Low	19	16	22	-3	6
							Jamaica	High	18	14	21	-5	8
**Slower increase from 2010 to 2019 compared with from 2001 to 2010**													
Grenada	Medium	17	19	19	2	0.4	India	Very low	25	26	26	0.9	0.3
Guatemala	High	13	14	15	2	0.5	Kenya	Very low	15	17	18	2	0.9
Philippines	Medium	16	17	18	2	0.9	Zimbabwe	Very low	25	29	30	4	1
Mozambique	Very low	13	19	21	6	2	Mozambique	Very low	25	34	35	9	2
Lesotho	Very low	17	31	33	14	2	Lesotho	Very low	27	37	40	10	3
**Faster increase from 2010 to 2019 compared with from 2001 to 2010**													
Peru	Medium	8	9	9	0.4	0.5	Peru	Medium	8	9	10	0.5	0.9
Honduras	Very low	17	17	19	1	1	Guatemala	High	13	13	15	0.7	1
Samoa	Very low	26	27	28	0.2	1	Haiti	Very low	28	28	30	0.2	1
Dominican Republic	Low	12	13	14	1	1	Nepal	Very low	18	18	21	0.0	3
							Dominican Republic	Low	17	18	21	0.8	3
							Honduras	Very low	14	14	18	0.4	4

■ Central Asia, Middle East and north Africa ■ Central and eastern Europe ■ East and southeast Asia ■ High-income western

■ Latin America and the Caribbean ■ Pacific Island nations ■ South Asia ■ Sub-Saharan Africa

Appendix Figure 1. Additional probability of dying from any NCD between birth and 70 or 80 years of age compared to the probability of dying from NCD4 between 30 and 70 years of age.

Each country is represented by a square and a circle, coloured by region. Squares show the difference between probability of dying from any NCD between birth and 70 years of age and probability of dying from NCD4 between 30 and 70 years of age. Circles show the difference between probability of dying from any NCD between birth and 80 years of age and probability of dying from NCD4 between 30 and 70 years of age.

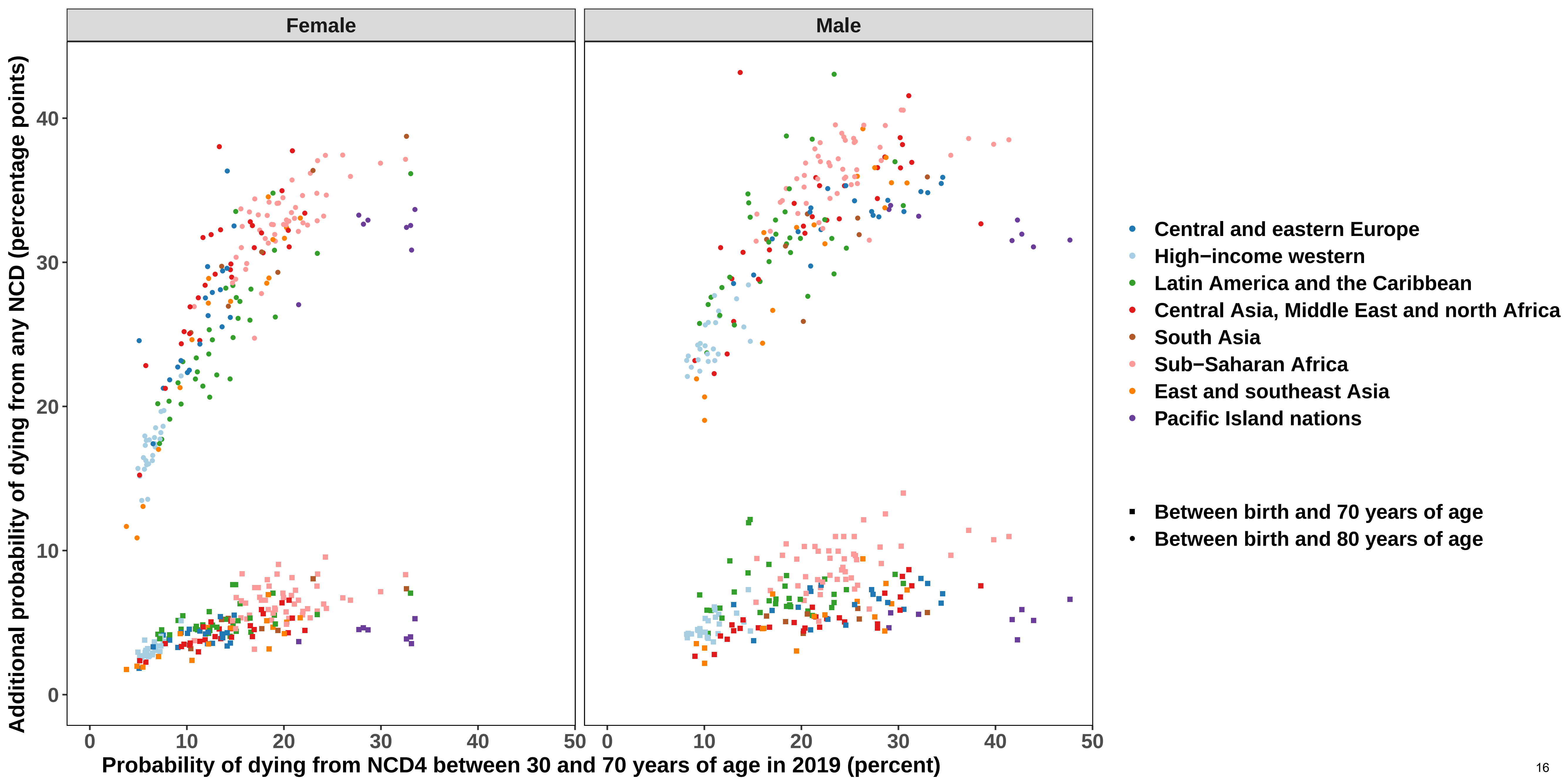
Female
Male
Additional probability of dying from any NCD (percentage points)
0
10
20
30
40
0
10
20
30
40
50
Probability of dying from NCD4 between 30 and 70 years of age in 2019 (percent)
Central and eastern Europe
High−income western
Latin America and the Caribbean
Central Asia, Middle East and north Africa
South Asia
Sub−Saharan Africa
East and southeast Asia
Pacific Island nations
Between birth and 70 years of age
Between birth and 80 years of age

Appendix Figure 2. Change in NCD mortality from 2010 to 2019 in relation to the level of mortality in 2010.

Each point represents one country, coloured by region. Data are shown for 185 countries in eight regions.

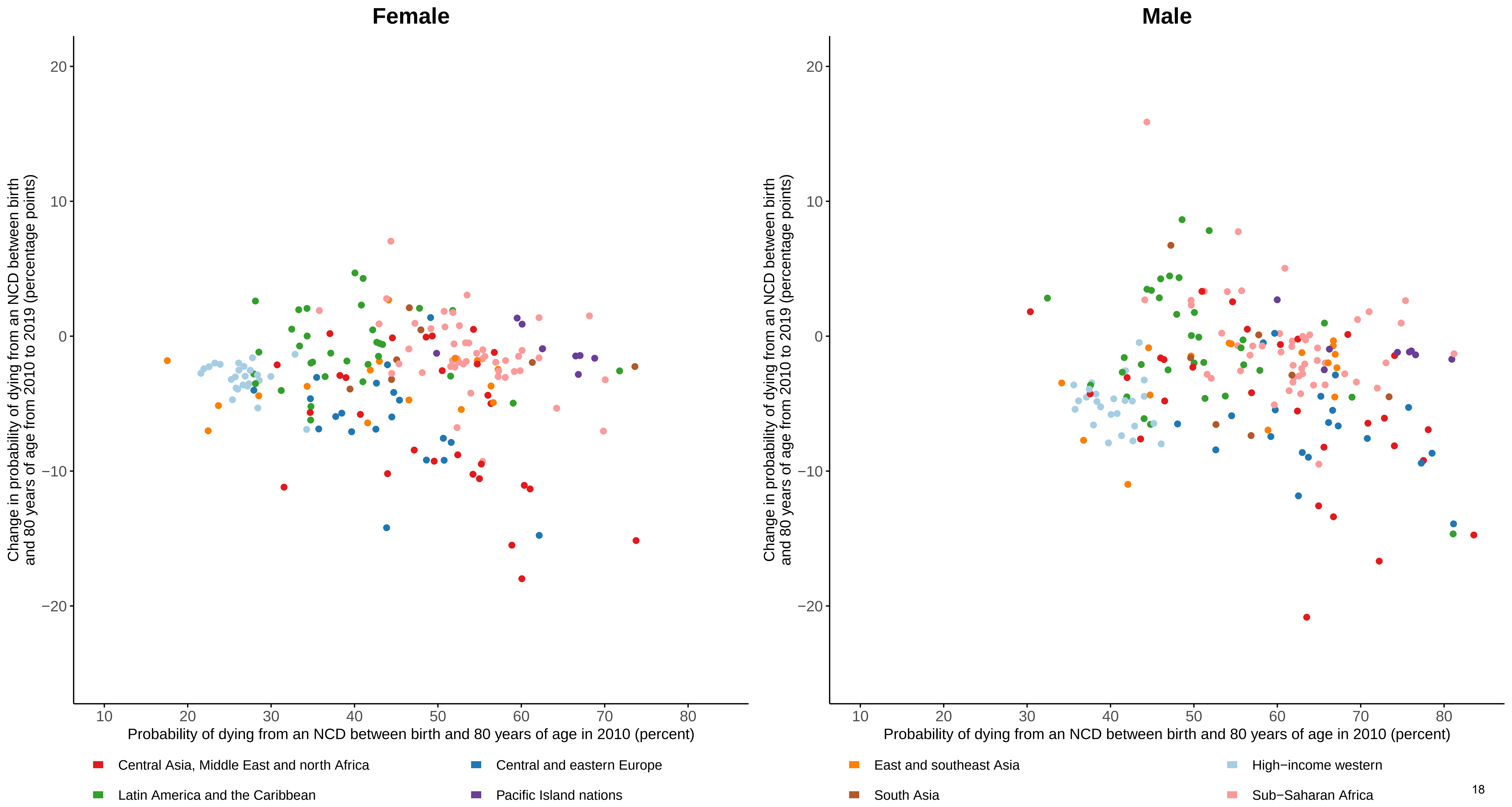

Female
Male
Change in probability of dying from an NCD between birth and 80 years of age from 2010 to 2019 (percentage points)
20
10
0
−10
−20
10
20
30
40
50
60
70
80
Probability of dying from an NCD between birth and 80 years of age in 2010 (percent)
Central Asia, Middle East and north Africa
Central and eastern Europe
East and southeast Asia
High−income western
Latin America and the Caribbean
Pacific Island nations
South Asia
Sub−Saharan Africa

Appendix Figure 3. Change in NCD mortality from 2010 to 2019 in females and males.

Each point represents one country, coloured by region. Data are shown for 185 countries in eight regions. The dotted diagonal line represents equal change from 2010 to 2019 in the probability of dying from an NCD between birth and 80 years of age for females and males. Segments of the plot are labelled to indicate the various relationships between the two sexes in the direction and size of change.

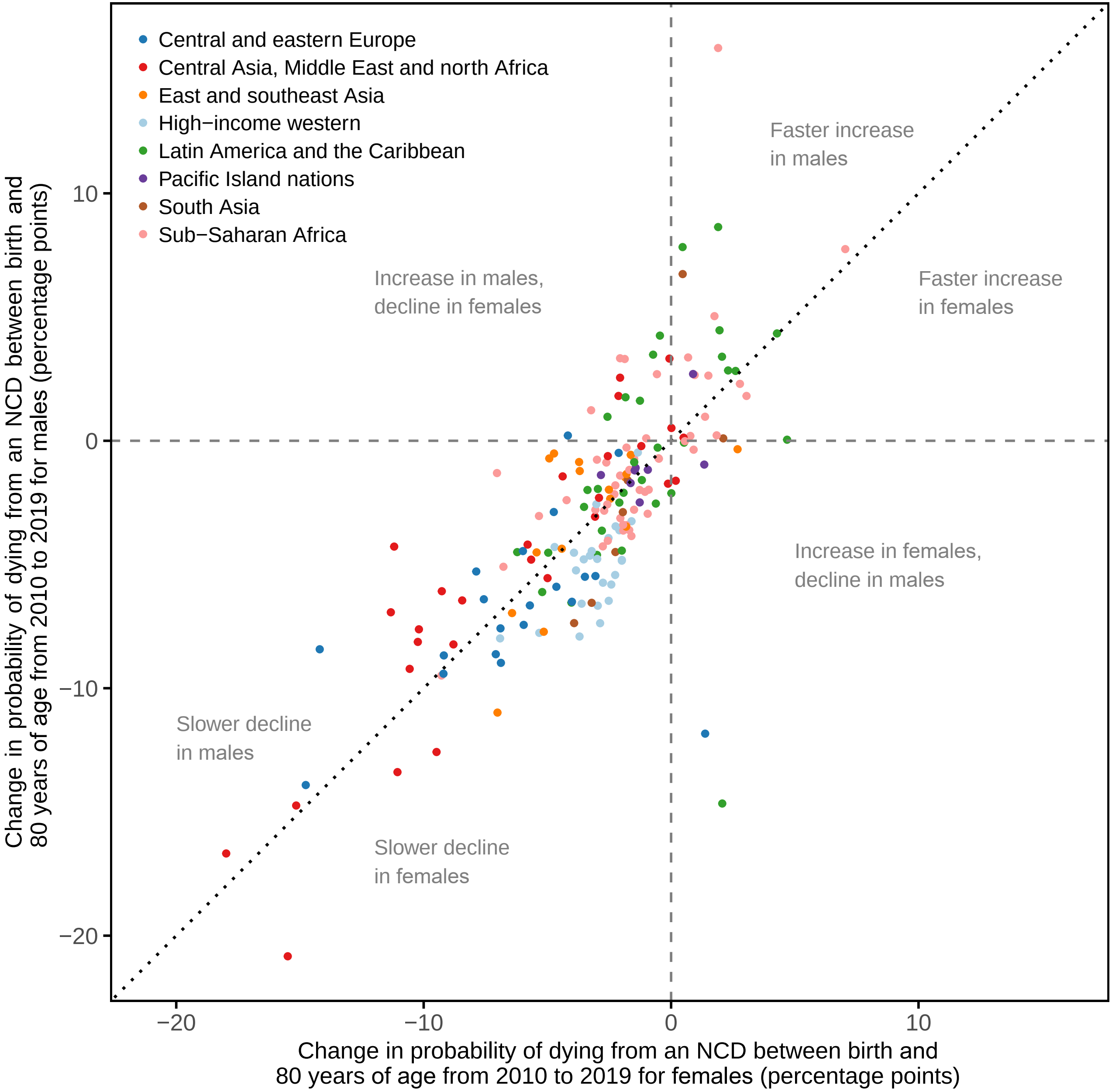
Central and eastern Europe
Central Asia, Middle East and north Africa
East and southeast Asia
High−income western
Latin America and the Caribbean
Pacific Island nations
South Asia
Sub−Saharan Africa
Faster increase in males
Increase in males, decline in females
Faster increase in females
Increase in females, decline in males
Slower decline in males
Slower decline in females
10
0
−10
−20
−20
−10
0
10
Change in probability of dying from an NCD between birth and 80 years of age from 2010 to 2019 for males (percentage points)
Change in probability of dying from an NCD between birth and 80 years of age from 2010 to 2019 for females (percentage points)

Appendix Figure 4. Age-specific death rates from NCDs in 2001, 2010 and 2019.

Each line represents the death rate from NCDs by five-year age group for one year. Death rates are shown for the years 2001, 2010, and 2019 for 64 countries, including 51 with high-quality data and 12 selected based on population size and medium data quality, as detailed in Methods. Death rates are displayed on the log scale.

Female

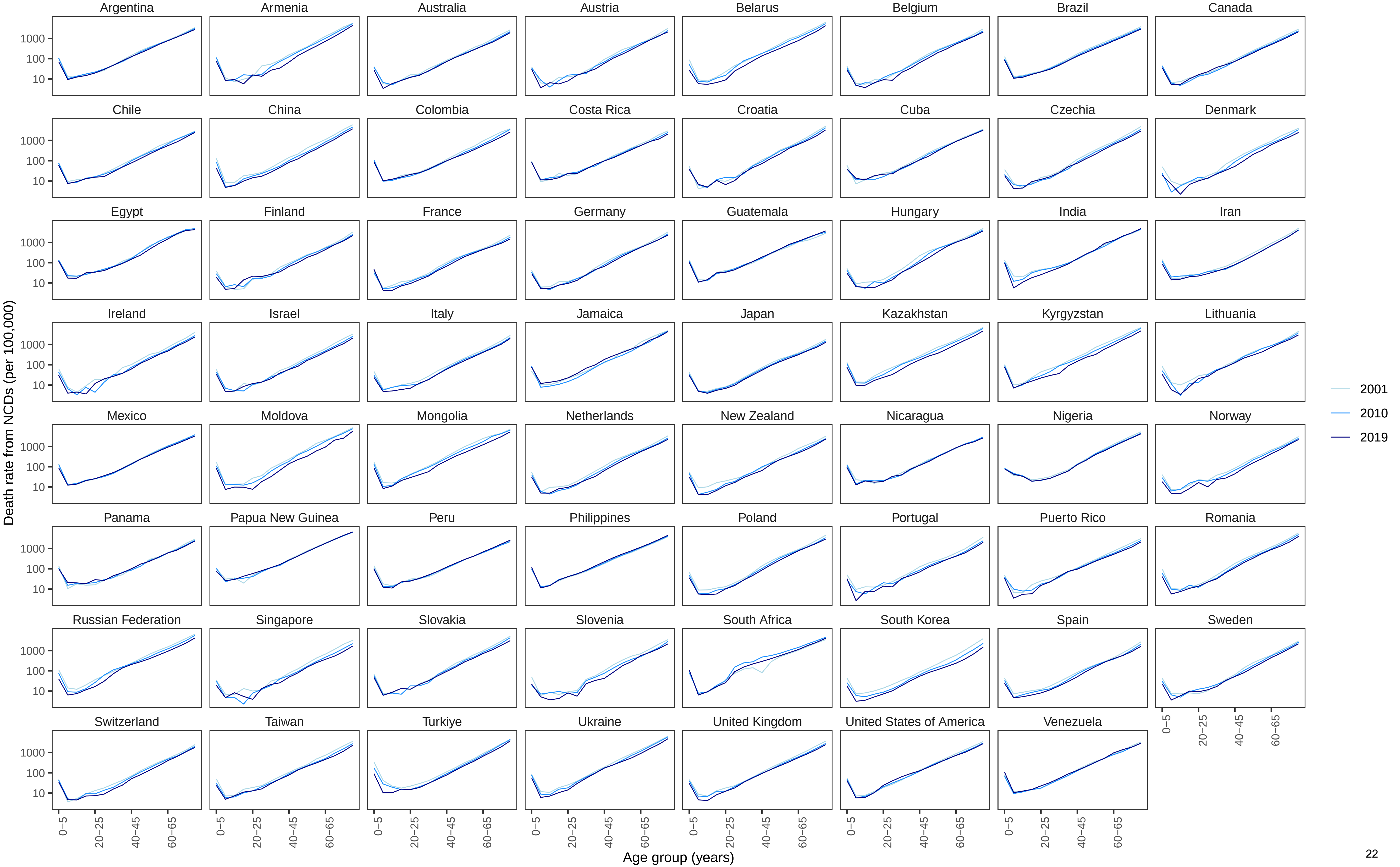

Argentina
Armenia
Australia
Austria
Belarus
Belgium
Brazil
Canada
Chile
China
Colombia
Costa Rica
Croatia
Cuba
Czechia
Denmark
Egypt
Finland
France
Germany
Guatemala
Hungary
India
Iran
Ireland
Israel
Italy
Jamaica
Japan
Kazakhstan
Kyrgyzstan
Lithuania
Mexico
Moldova
Mongolia
Netherlands
New Zealand
Nicaragua
Nigeria
Norway
Panama
Papua New Guinea
Peru
Philippines
Poland
Portugal
Puerto Rico
Romania
Russian Federation
Singapore
Slovakia
Slovenia
South Africa
South Korea
Spain
Sweden
Switzerland
Taiwan
Turkiye
Ukraine
United Kingdom
United States of America
Venezuela
Death rate from NCDs (per 100,000)
1000
100
10
Age group (years)
0–5
20–25
40–45
60–65
2001
2010
2019

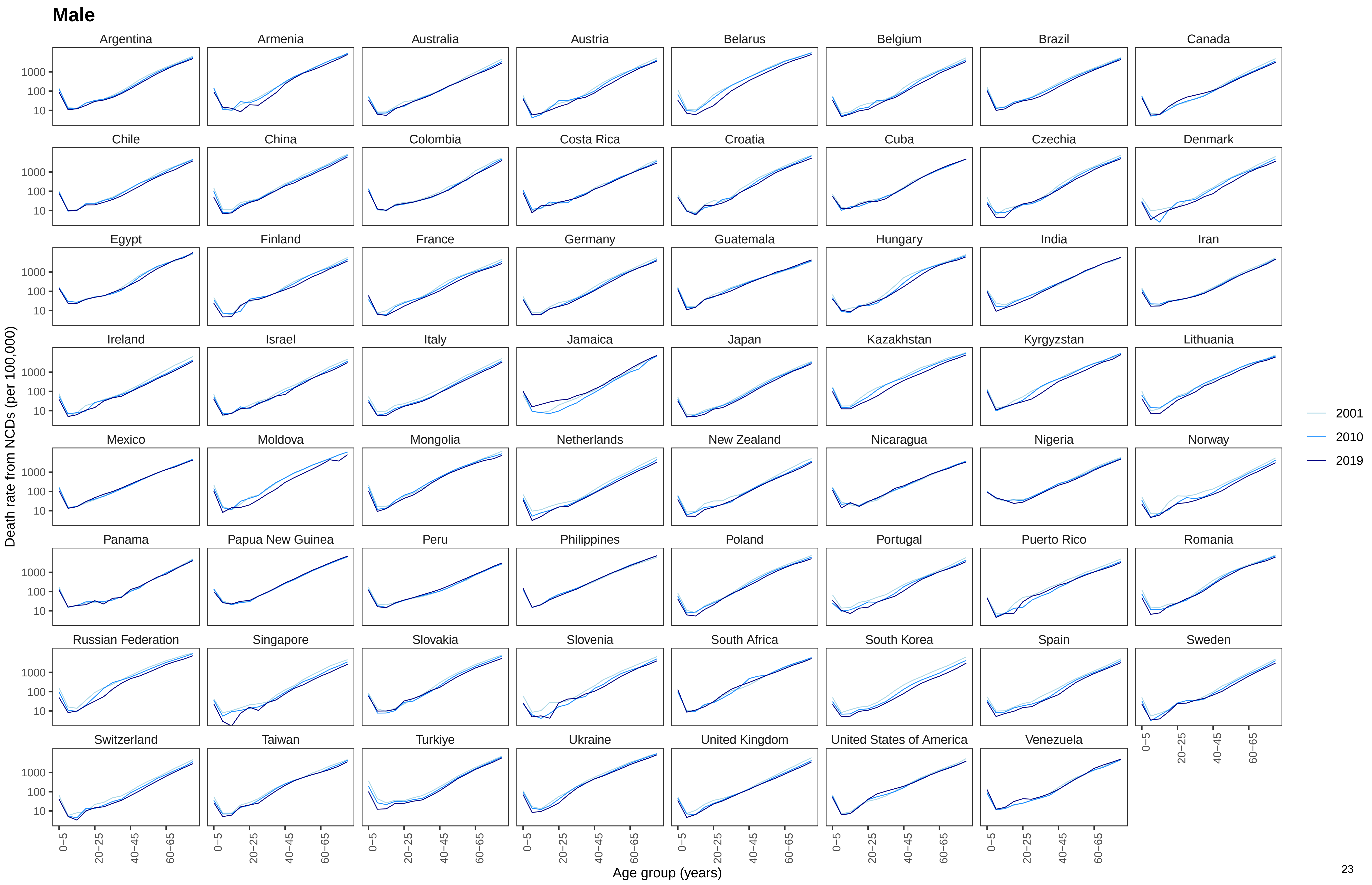
Male
Argentina
Armenia
Australia
Austria
Belarus
Belgium
Brazil
Canada
Chile
China
Colombia
Costa Rica
Croatia
Cuba
Czechia
Denmark
Egypt
Finland
France
Germany
Guatemala
Hungary
India
Iran
Ireland
Israel
Italy
Jamaica
Japan
Kazakhstan
Kyrgyzstan
Lithuania
Mexico
Moldova
Mongolia
Netherlands
New Zealand
Nicaragua
Nigeria
Norway
Panama
Papua New Guinea
Peru
Philippines
Poland
Portugal
Puerto Rico
Romania
Russian Federation
Singapore
Slovakia
Slovenia
South Africa
South Korea
Spain
Sweden
Switzerland
Taiwan
Turkiye
Ukraine
United Kingdom
United States of America
Venezuela
Death rate from NCDs (per 100,000)
Age group (years)
1000
100
10
0–5
20–25
40–45
60–65
2001
2010
2019

Appendix Figure 5. Comparison of change in NCD mortality between birth and 80 years of age from 2010 to 2019 with change from 2001 to 2010.

Each point represents one country, coloured by region. Data are shown for 185 countries in eight regions. The dotted diagonal line represents equal absolute change in probability of death from 2001 to 2010 and from 2010 to 2019. Segments of the plot are labelled to indicate whether countries experienced a change in the direction or magnitude of change in the probability of death from one decade to the next.

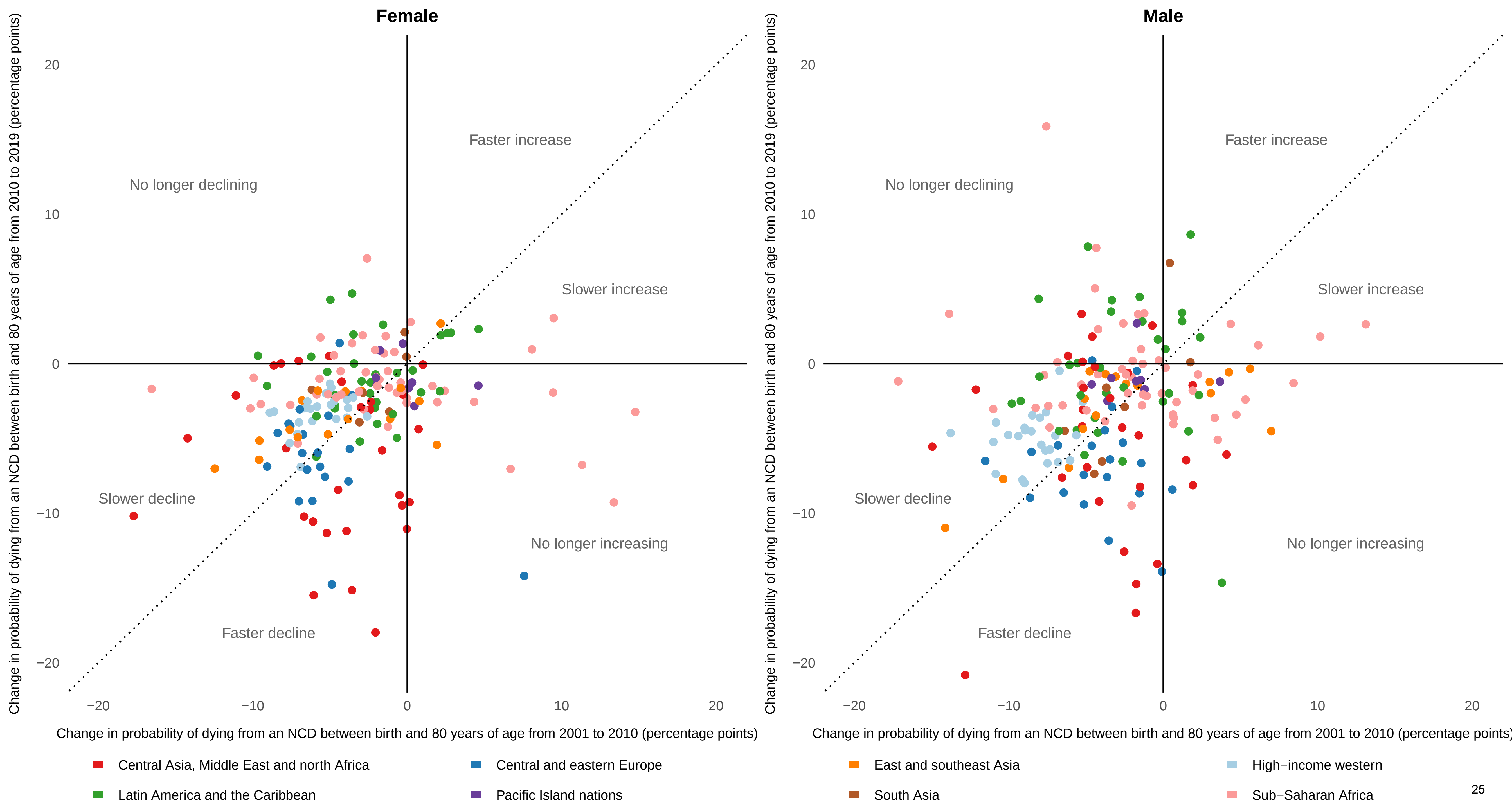

Female
Male
Faster increase
No longer declining
Slower increase
Slower decline
No longer increasing
Faster decline
Change in probability of dying from an NCD between birth and 80 years of age from 2010 to 2019 (percentage points)
Change in probability of dying from an NCD between birth and 80 years of age from 2001 to 2010 (percentage points)
20
10
0
−10
−20
Central Asia, Middle East and north Africa
Central and eastern Europe
East and southeast Asia
High−income western
Latin America and the Caribbean
Pacific Island nations
South Asia
Sub−Saharan Africa

Appendix Figure 6. Percentage point contributions of mortality from different NCD causes of death and in different age groups to overall change in NCD mortality from 2010 to 2019.

Panel A shows the contribution of 20 mutually exclusive, collectively exhaustive NCD causes of death to the change in the probability of dying from an NCD between birth and 80 years of age from 2010 to 2019. Each column represents a cause of death with causes arranged by disease category. **Panel B** shows the contribution of five-year age groups to the change in this probability over the same period, with each column representing a five-year age group.

In both panels, each row represents a country. Results are shown for 63 countries, of which 51 were identified as having high-quality data and 12 were selected based on population size, as detailed in Methods. These 12 countries are denoted with asterisks. Countries are grouped and coloured by region and ordered from the largest decrease to the smallest decrease or largest increase in the probability of dying from an NCD between birth and 80 years of age from 2010 to 2019. Each tile shows the absolute contribution of an NCD cause of death or age group to the total change in this probability for one country, also written on the tile in percentage points. See Figure 7 caption for use of colour palettes.

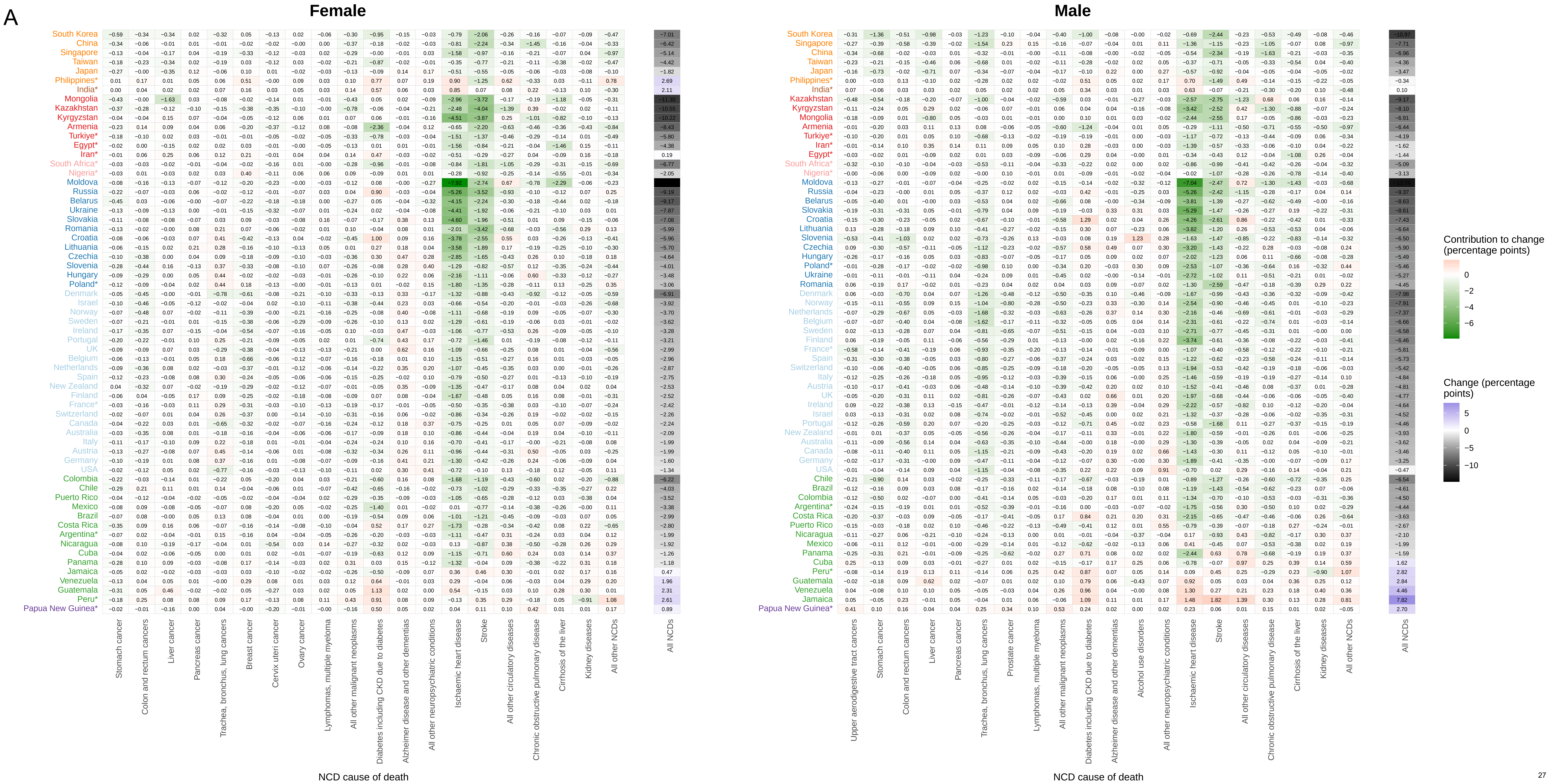

A
Female
Male
South Korea
China
Singapore
Taiwan
Japan
Philippines*
India*
Mongolia
Kazakhstan
Kyrgyzstan
Armenia
Turkiye*
Egypt*
Iran*
South Africa*
Nigeria*
Moldova
Russia
Belarus
Ukraine
Slovakia
Romania
Croatia
Lithuania
Czechia
Slovenia
Hungary
Poland*
Denmark
Israel
Norway
Sweden
Ireland
Portugal
UK
Belgium
Netherlands
Spain
New Zealand
Finland
France*
Switzerland
Canada
Australia
Italy
Austria
Germany
USA
Colombia
Chile
Puerto Rico
Mexico
Brazil
Costa Rica
Argentina*
Nicaragua
Cuba
Panama
Jamaica
Venezuela
Guatemala
Peru*
Papua New Guinea*
Stomach cancer
Colon and rectum cancers
Liver cancer
Pancreas cancer
Trachea, bronchus, lung cancers
Breast cancer
Cervix uteri cancer
Ovary cancer
Lymphomas, multiple myeloma
All other malignant neoplasms
Diabetes including CKD due to diabetes
Alzheimer disease and other dementias
All other neuropsychiatric conditions
Ischaemic heart disease
Stroke
All other circulatory diseases
Chronic obstructive pulmonary disease
Cirrhosis of the liver
Kidney diseases
All other NCDs
All NCDs
NCD cause of death
Upper aerodigestive tract cancers
Prostate cancer
Alcohol use disorders
Contribution to change (percentage points)
0
−2
−4
−6
Change (percentage points)
5
0
−5
−10

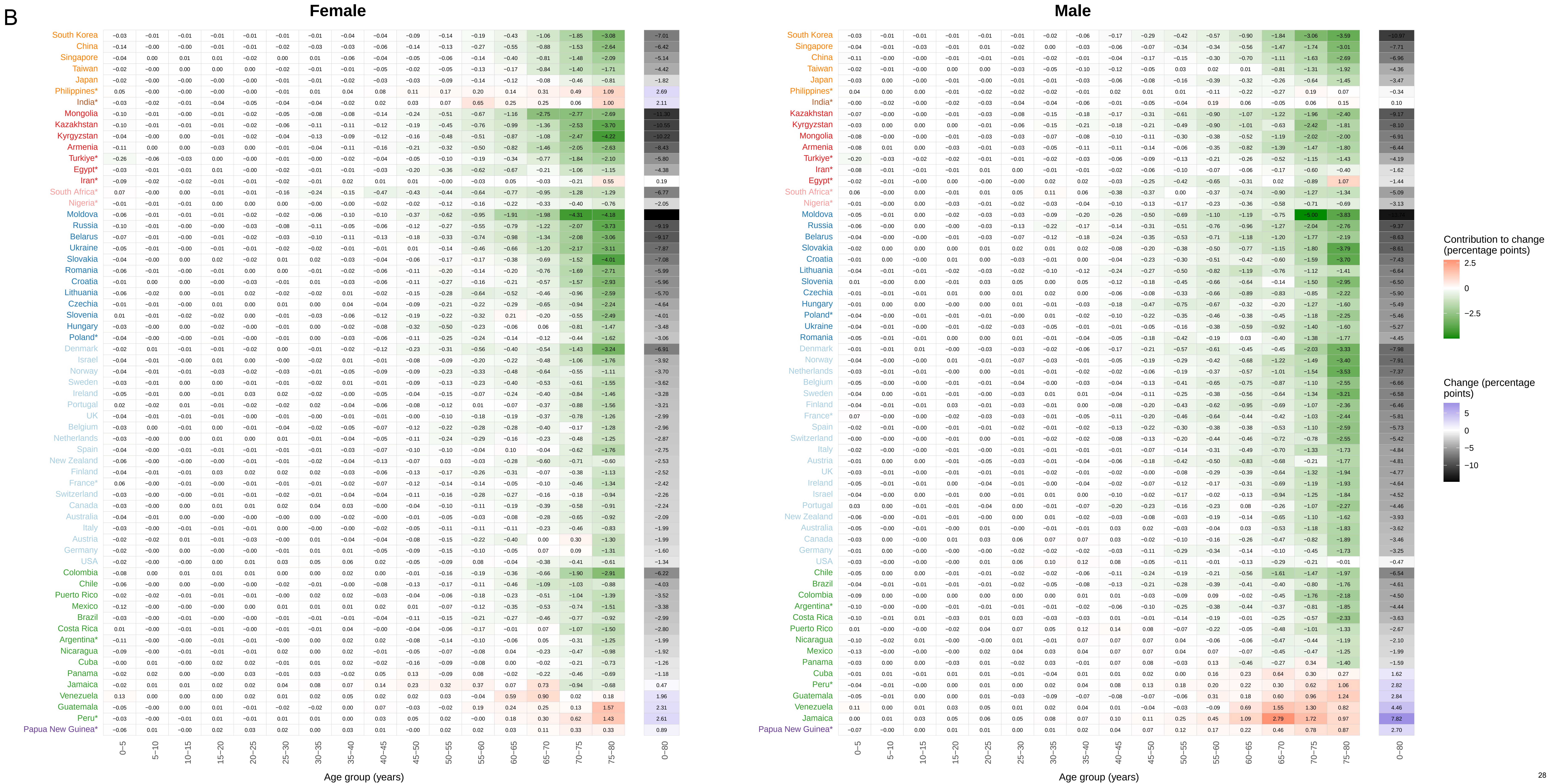
B
Female
Male
South Korea
China
Singapore
Taiwan
Japan
Philippines*
India*
Mongolia
Kazakhstan
Kyrgyzstan
Armenia
Turkiye*
Egypt*
Iran*
South Africa*
Nigeria*
Moldova
Russia
Belarus
Ukraine
Slovakia
Romania
Croatia
Lithuania
Czechia
Slovenia
Hungary
Poland*
Denmark
Israel
Norway
Sweden
Ireland
Portugal
UK
Belgium
Netherlands
Spain
New Zealand
Finland
France*
Switzerland
Canada
Australia
Italy
Austria
Germany
USA
Colombia
Chile
Puerto Rico
Mexico
Brazil
Costa Rica
Argentina*
Nicaragua
Cuba
Panama
Jamaica
Venezuela
Guatemala
Peru*
Papua New Guinea*
0–5
5–10
10–15
15–20
20–25
25–30
30–35
35–40
40–45
45–50
50–55
55–60
60–65
65–70
70–75
75–80
0–80
Age group (years)
Contribution to change (percentage points)
2.5
0
−2.5
Change (percentage points)
5
0
−5
−10

Appendix Figure 7. Percentage point contributions of mortality from different NCD causes of death and in different age groups to slowdown or acceleration of change in NCD mortality from 2010 to 2019 compared to change from 2001 to 2010.

Panel A shows the contribution of 20 mutually exclusive, collectively exhaustive NCD causes of death to the difference in change in the probability of dying from an NCD between birth and 80 years of age between two decades (from 2010 to 2019, and from 2001 to 2010). Each column represents a cause of death, as stated in Table 1, with causes arranged by disease category. **Panel B** shows the contribution of five-year age groups to this decadal difference in change, with each column representing a five-year age group.

In both panels, each row represents a country. Results are shown for 63 countries, of which 51 were identified as having high-quality data and 12 were selected based on population size, as detailed in Methods. These 12 countries are denoted with asterisks. Countries are grouped and coloured by region and ordered from the largest improvement in NCD mortality from 2010 to 2019 compared to the preceding decade to the largest deterioration. Each tile shows the absolute contribution of a specific NCD cause of death or age group to the decadal difference in change in NCD mortality between the two decades for one country, also written on the tile in percentage points. See Figure 8 caption for use of colour palettes.

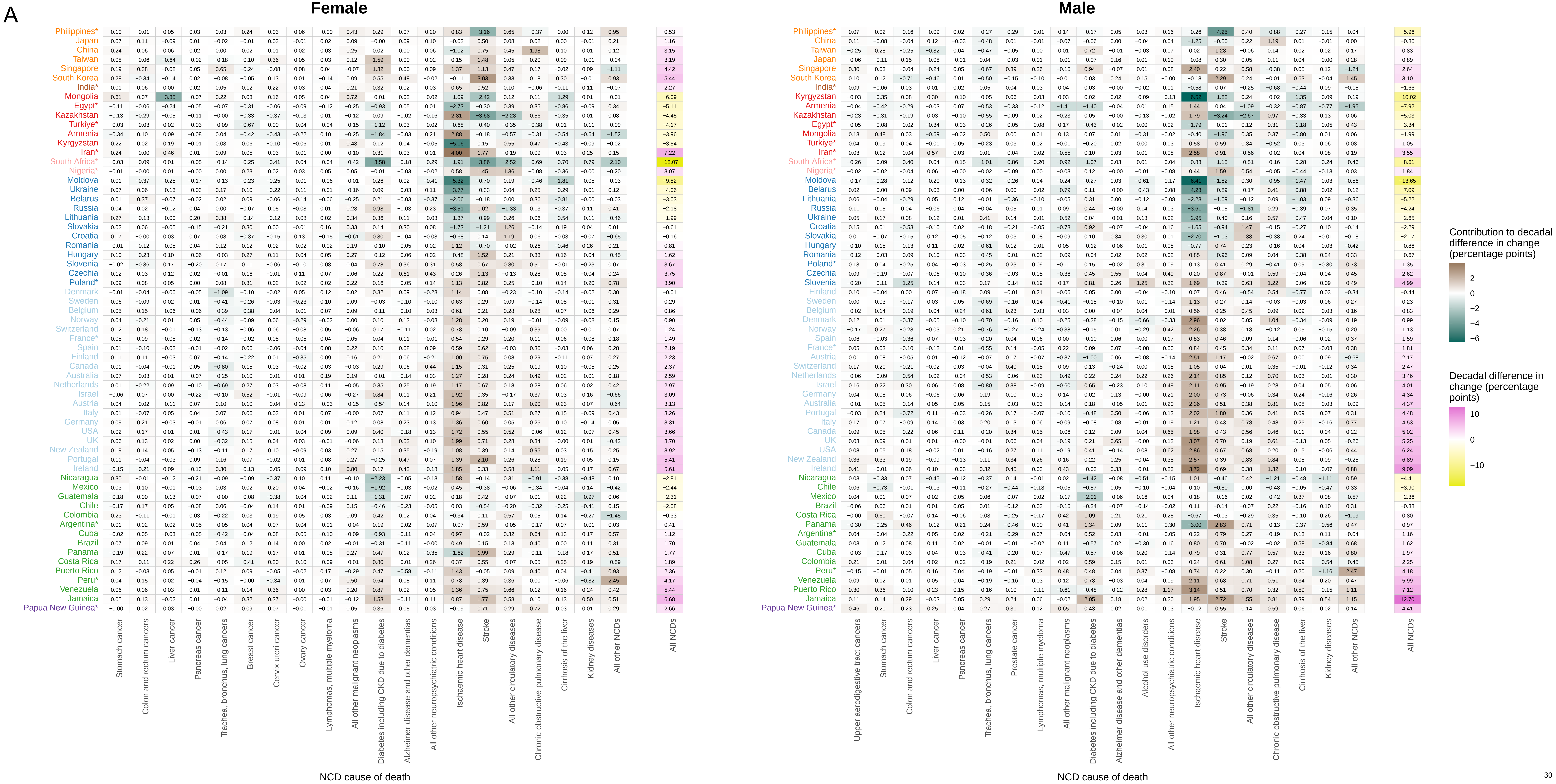
A
Female
Male
NCD cause of death
NCD cause of death
All NCDs
Contribution to decadal difference in change (percentage points)
Decadal difference in change (percentage points)

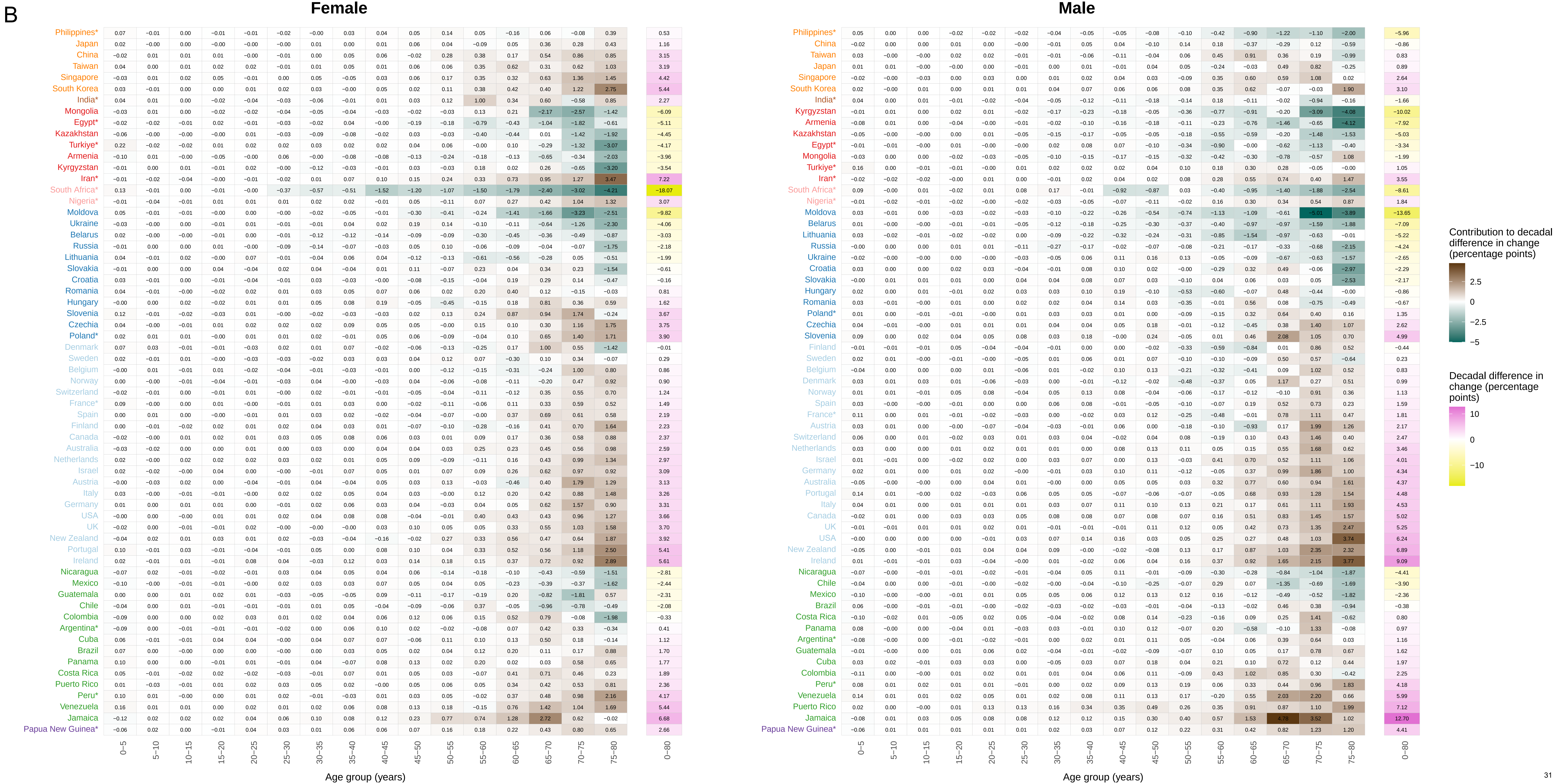

B
Female
Male
Philippines*
Japan
China
Taiwan
Singapore
South Korea
India*
Mongolia
Egypt*
Kazakhstan
Turkiye*
Armenia
Kyrgyzstan
Iran*
South Africa*
Nigeria*
Moldova
Ukraine
Belarus
Russia
Lithuania
Slovakia
Croatia
Romania
Hungary
Slovenia
Czechia
Poland*
Denmark
Sweden
Belgium
Norway
Switzerland
France*
Spain
Finland
Canada
Australia
Netherlands
Israel
Austria
Italy
Germany
USA
UK
New Zealand
Portugal
Ireland
Nicaragua
Mexico
Guatemala
Chile
Colombia
Argentina*
Cuba
Brazil
Panama
Costa Rica
Puerto Rico
Peru*
Venezuela
Jamaica
Papua New Guinea*
0–5
5–10
10–15
15–20
20–25
25–30
30–35
35–40
40–45
45–50
50–55
55–60
60–65
65–70
70–75
75–80
0–80
Age group (years)
Contribution to decadal difference in change (percentage points)
2.5
0
−2.5
−5
Decadal difference in change (percentage points)
10
0
−10

Appendix Figure 8. Percentage point contributions of mortality from different NCD causes of death and in different age groups to how much NCD mortality in each country lags its regional benchmark.

Panel A shows the contribution of 20 mutually exclusive, collectively exhaustive NCD causes of death to the difference in the change in the probability of dying from an NCD between birth and 80 years of age from 2010 to 2019, relative to a country benchmark within each region. Benchmarks are identified as the country in each region with the largest reduction in NCD mortality over this period. Each column represents a cause of death, as stated in Table 1, with causes arranged by disease category. **Panel B** shows the contribution of five-year age groups to this difference, with each column representing a five-year age group.

In both panels, each row represents a country. Results are shown for 51 countries identified as having high-quality data, as detailed in Methods. Countries are grouped and coloured by region and ordered from the largest decrease to the smallest decrease or largest increase in the probability of dying from an NCD between birth and 80 years of age from 2010 to 2019. The benchmark for each region is the country in the first row of its region grouping and is shown in bold font. Each tile shows the absolute contribution of a specific NCD cause of death or age group to the difference in change relative to the benchmark country for one country, also written on the tile in percentage points. See Figure 9 caption for use of colour palettes.

A

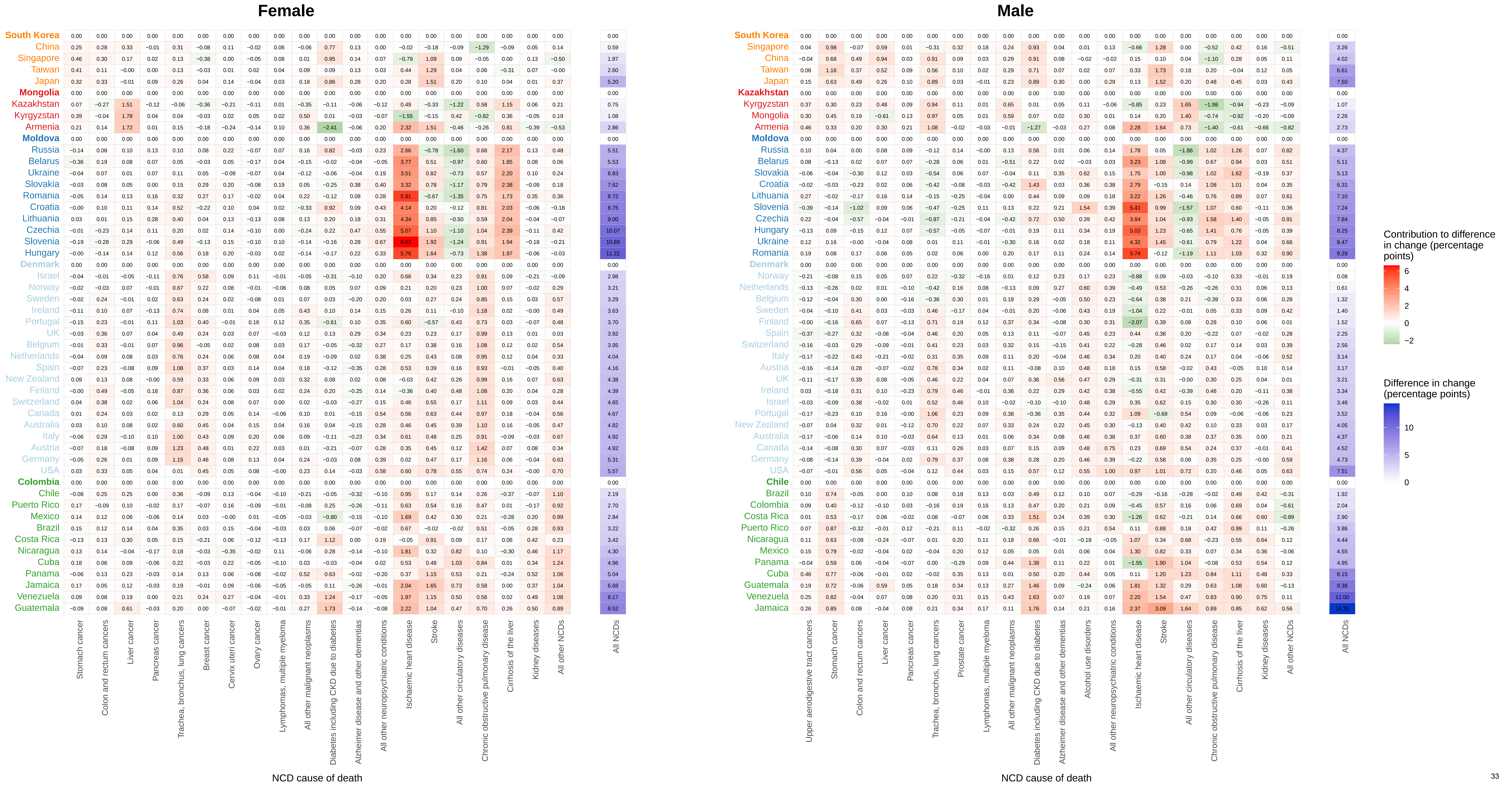
Female
Male
South Korea
China
Singapore
Taiwan
Japan
Mongolia
Kazakhstan
Kyrgyzstan
Armenia
Moldova
Russia
Belarus
Ukraine
Slovakia
Romania
Croatia
Lithuania
Czechia
Slovenia
Hungary
Denmark
Israel
Norway
Sweden
Ireland
Portugal
UK
Belgium
Netherlands
Spain
New Zealand
Finland
Switzerland
Canada
Australia
Italy
Austria
Germany
USA
Colombia
Chile
Puerto Rico
Mexico
Brazil
Costa Rica
Nicaragua
Cuba
Panama
Jamaica
Venezuela
Guatemala
Stomach cancer
Colon and rectum cancers
Liver cancer
Pancreas cancer
Trachea, bronchus, lung cancers
Breast cancer
Cervix uteri cancer
Ovary cancer
Lymphomas, multiple myeloma
All other malignant neoplasms
Diabetes including CKD due to diabetes
Alzheimer disease and other dementias
All other neuropsychiatric conditions
Ischaemic heart disease
Stroke
All other circulatory diseases
Chronic obstructive pulmonary disease
Cirrhosis of the liver
Kidney diseases
All other NCDs
All NCDs
Upper aerodigestive tract cancers
Prostate cancer
Alcohol use disorders
NCD cause of death
Contribution to difference in change (percentage points)
Difference in change (percentage points)

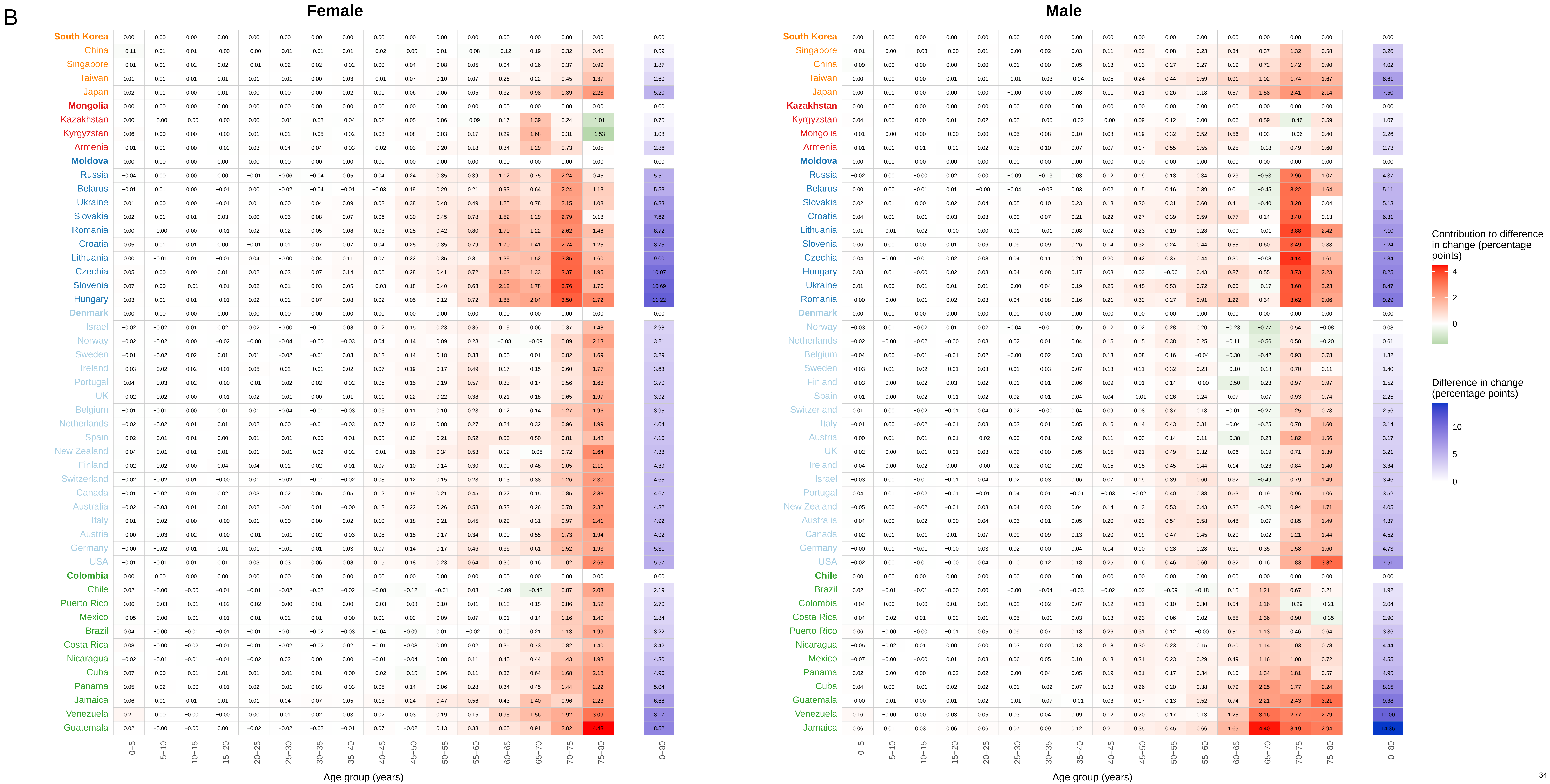
B
Female
South Korea
China
Singapore
Taiwan
Japan
Mongolia
Kazakhstan
Kyrgyzstan
Armenia
Moldova
Russia
Belarus
Ukraine
Slovakia
Romania
Croatia
Lithuania
Czechia
Slovenia
Hungary
Denmark
Israel
Norway
Sweden
Ireland
Portugal
UK
Belgium
Netherlands
Spain
New Zealand
Finland
Switzerland
Canada
Australia
Italy
Austria
Germany
USA
Colombia
Chile
Puerto Rico
Mexico
Brazil
Costa Rica
Nicaragua
Cuba
Panama
Jamaica
Venezuela
Guatemala
Male
South Korea
Singapore
China
Taiwan
Japan
Kazakhstan
Kyrgyzstan
Mongolia
Armenia
Moldova
Russia
Belarus
Slovakia
Croatia
Lithuania
Slovenia
Czechia
Hungary
Ukraine
Romania
Denmark
Norway
Netherlands
Belgium
Sweden
Finland
Spain
Switzerland
Italy
Austria
UK
Ireland
Israel
Portugal
New Zealand
Australia
Canada
Germany
USA
Chile
Brazil
Colombia
Costa Rica
Puerto Rico
Nicaragua
Mexico
Panama
Cuba
Guatemala
Venezuela
Jamaica
0–5
5–10
10–15
15–20
20–25
25–30
30–35
35–40
40–45
45–50
50–55
55–60
60–65
65–70
70–75
75–80
0–80
Age group (years)
Contribution to difference in change (percentage points)
4
2
0
Difference in change (percentage points)
10
5
0

Appendix Figure 9. Contributions of mortality from aggregated NCD causes of death to overall change in NCD mortality from 2010 to 2019.

The figure shows the contribution of four mutually exclusive, collectively exhaustive aggregated NCD causes of death to the change in the probability of dying from an NCD between birth and 80 years of age from 2010 to 2019. Each column represents a group of causes of death. Each row represents a country.

Results are shown for 63 countries, of which 51 were identified as having high-quality data and 12 were selected based on population size, as detailed in Methods. These 12 countries are denoted with asterisks. Countries are grouped and coloured by region and ordered from the largest decrease to the smallest decrease or largest increase in the probability of dying from an NCD between birth and 80 years of age from 2010 to 2019. Each tile shows the absolute contribution of a group of NCD causes of death to the total change in this probability for one country. Two colour palettes are used: one for the overall change in NCD mortality from 2010 to 2019, and one for contributions of aggregated NCD causes of death. For overall change, black indicates a decrease, blue an increase, and white no change. For contributions to change, green indicates a contribution to lowering NCD mortality, red a contribution to increasing it, and white a contribution of zero.

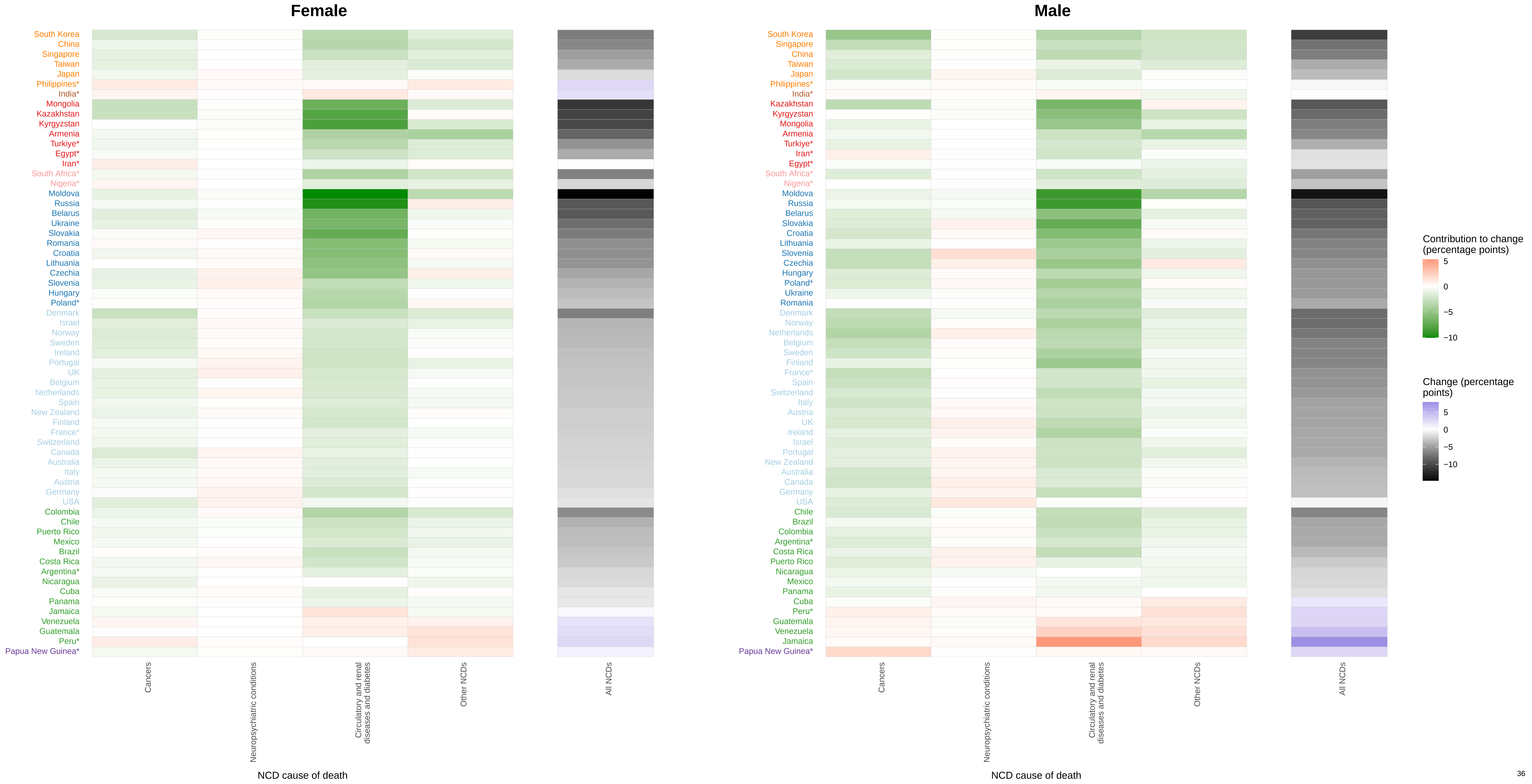

Female
Male
South Korea
China
Singapore
Taiwan
Japan
Philippines*
India*
Mongolia
Kazakhstan
Kyrgyzstan
Armenia
Turkiye*
Egypt*
Iran*
South Africa*
Nigeria*
Moldova
Russia
Belarus
Ukraine
Slovakia
Romania
Croatia
Lithuania
Czechia
Slovenia
Hungary
Poland*
Denmark
Israel
Norway
Sweden
Ireland
Portugal
UK
Belgium
Netherlands
Spain
New Zealand
Finland
France*
Switzerland
Canada
Australia
Italy
Austria
Germany
USA
Colombia
Chile
Puerto Rico
Mexico
Brazil
Costa Rica
Argentina*
Nicaragua
Cuba
Panama
Jamaica
Venezuela
Guatemala
Peru*
Papua New Guinea*
Cancers
Neuropsychiatric conditions
Circulatory and renal diseases and diabetes
Other NCDs
All NCDs
NCD cause of death
Contribution to change (percentage points)
5
0
−5
−10
Change (percentage points)
5
0
−5
−10

Appendix Figure 10. Contributions of mortality from aggregated NCD causes of death to slowdown or acceleration of change in NCD mortality from 2010 to 2019 compared to change from 2001 to 2010.

The figure shows the contribution of four mutually exclusive, collectively exhaustive aggregated NCD causes of death to the decadal difference in change in the probability of dying from an NCD between birth and 80 years of age between two decades (from 2010 to 2019, and from 2001 to 2010). Each column represents a group of causes of death. Each row represents a country.

Results are shown for 63 countries, of which 51 were identified as having high-quality data and 12 were selected based on population size, as detailed in Methods. These 12 countries are denoted with asterisks. Countries are grouped and coloured by region and ordered from the largest improvement in NCD mortality from 2010 to 2019 compared to the preceding decade to the largest deterioration. Each tile shows the absolute contribution of a group of NCD causes of death to the decadal difference in change in NCD mortality between the two decades for one country. Two colour palettes are used: one for the overall decadal difference in change, and one for contributions of aggregated NCD causes of death. For overall decadal difference in change, yellow indicates improvement (a larger decline, smaller increase or reversal of an increase), magenta deterioration (a smaller decline, reversal of a decline or a larger increase), and white no difference in the magnitude of change. For contributions to decadal difference in change, teal indicates a contribution to improvement of trend (a larger decline, smaller increase or reversal of an increase), brown a contribution to deterioration of trend (a smaller decline, reversal of a decline or a larger increase), and white a contribution of zero.

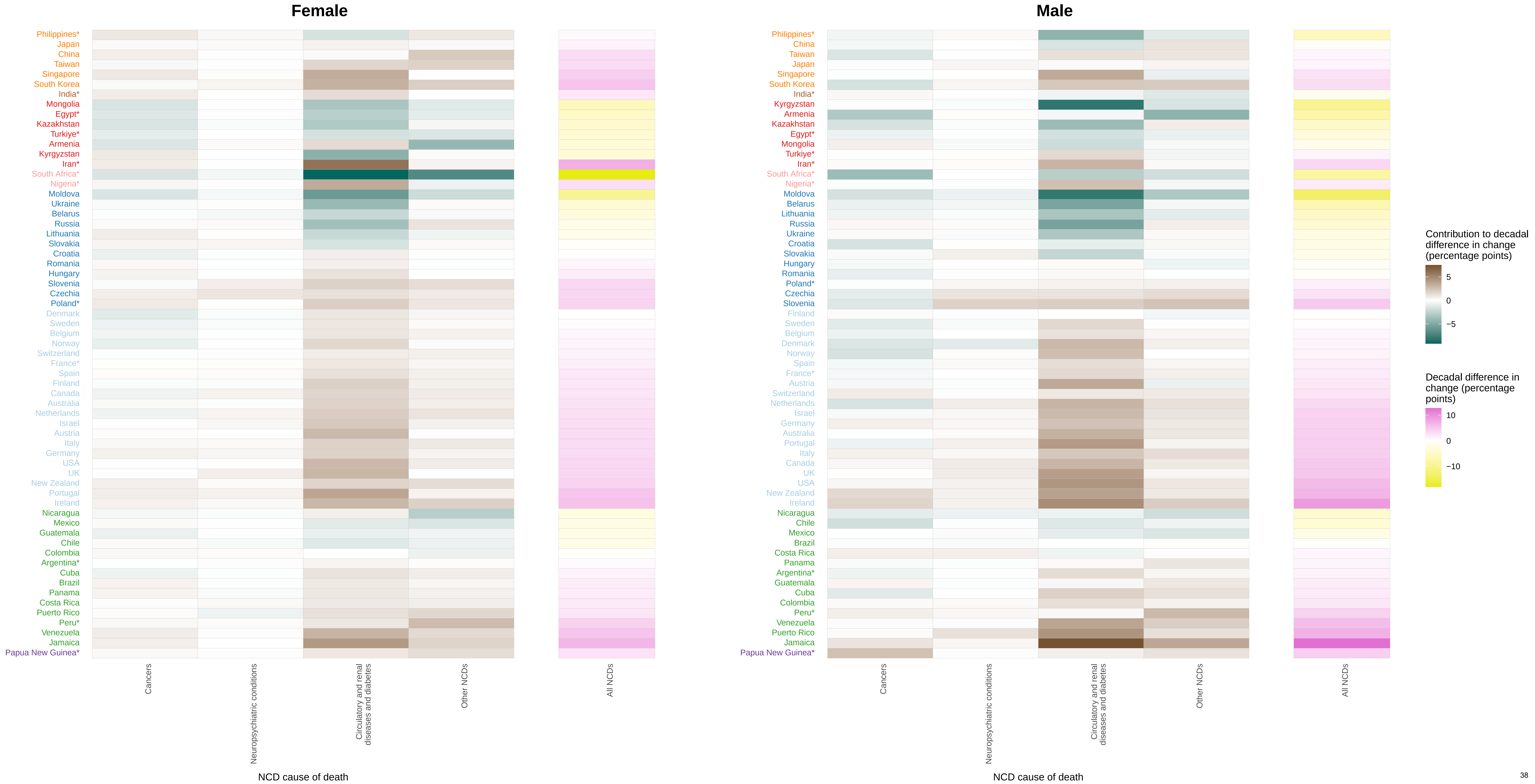
Female
Male
Philippines*
Japan
China
Taiwan
Singapore
South Korea
India*
Mongolia
Egypt*
Kazakhstan
Turkiye*
Armenia
Kyrgyzstan
Iran*
South Africa*
Nigeria*
Moldova
Ukraine
Belarus
Russia
Lithuania
Slovakia
Croatia
Romania
Hungary
Slovenia
Czechia
Poland*
Denmark
Sweden
Belgium
Norway
Switzerland
France*
Spain
Finland
Canada
Australia
Netherlands
Israel
Austria
Italy
Germany
USA
UK
New Zealand
Portugal
Ireland
Nicaragua
Mexico
Guatemala
Chile
Colombia
Argentina*
Cuba
Brazil
Panama
Costa Rica
Puerto Rico
Peru*
Venezuela
Jamaica
Papua New Guinea*
Cancers
Neuropsychiatric conditions
Circulatory and renal diseases and diabetes
Other NCDs
All NCDs
NCD cause of death
Contribution to decadal difference in change (percentage points)
5
0
−5
Decadal difference in change (percentage points)
10
0
−10

Appendix Figure 11. Contributions of mortality from aggregated NCD causes of death to how much NCD mortality in each country lags its regional benchmark.

The figure shows the contribution of four mutually exclusive, collectively exhaustive aggregated NCD causes of death to the difference in the change in the probability of dying from an NCD between birth and 80 years of age from 2010 to 2019, relative to a country benchmark within each region. Benchmarks are identified as the country in each region with the largest reduction in NCD mortality over this period. Each column represents a group of causes of death. Each row represents a country.

Results are shown for 51 countries identified as having high-quality data, as detailed in Methods. Countries are grouped and coloured by region and ordered from the largest decrease to the smallest decrease or largest increase in the probability of dying from an NCD between birth and 80 years of age from 2010 to 2019. The benchmark for each region is the country in the first row of its region grouping and is shown in bold font. Each tile shows the absolute contribution of a group of NCD causes of death to the difference in change relative to the benchmark country for one country. Two colour palettes are used: one for the overall difference in change relative to the benchmark, and one for contributions of aggregated NCD causes of death relative to those of the benchmark. For overall difference in change, black indicates a decrease relative to the benchmark, blue an increase relative to the benchmark, and white no difference in change. For each contribution to difference in change, green indicates a contribution towards a larger decline or smaller increase relative to the benchmark, red a contribution towards a smaller decline or larger increase relative to the benchmark, and white a contribution of zero.

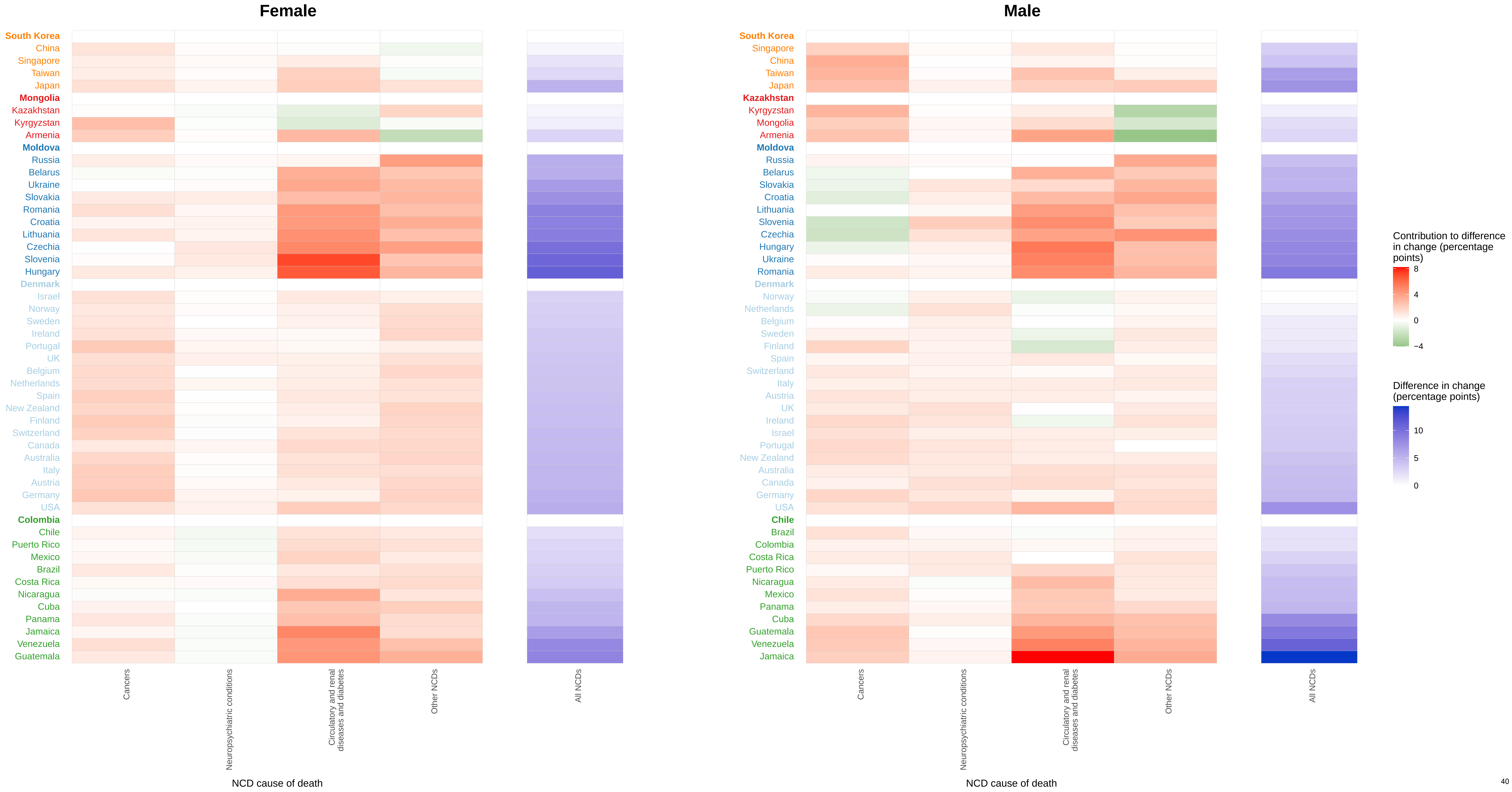
Female
Male
South Korea
China
Singapore
Taiwan
Japan
Mongolia
Kazakhstan
Kyrgyzstan
Armenia
Moldova
Russia
Belarus
Ukraine
Slovakia
Romania
Croatia
Lithuania
Czechia
Slovenia
Hungary
Denmark
Israel
Norway
Sweden
Ireland
Portugal
UK
Belgium
Netherlands
Spain
New Zealand
Finland
Switzerland
Canada
Australia
Italy
Austria
Germany
USA
Colombia
Chile
Puerto Rico
Mexico
Brazil
Costa Rica
Nicaragua
Cuba
Panama
Jamaica
Venezuela
Guatemala
Cancers
Neuropsychiatric conditions
Circulatory and renal diseases and diabetes
Other NCDs
All NCDs
NCD cause of death
Contribution to difference in change (percentage points)
8
4
0
−4
Difference in change (percentage points)
10
5
0

Appendix Figure 12. Probability of dying around and after the COVID-19 pandemic.

Each line represents the probability of dying for both sexes combined for a specified group of causes from 2001 to 2022 or 2023. Trends for 17 countries from three regions (high-income western, central and eastern Europe, and east and southeast Asia) with high-quality data through 2022 or 2023 are displayed. Dashed lines highlight 2020 and the impact of the COVID-19 pandemic. Probabilities of dying are displayed on a logarithmic scale.

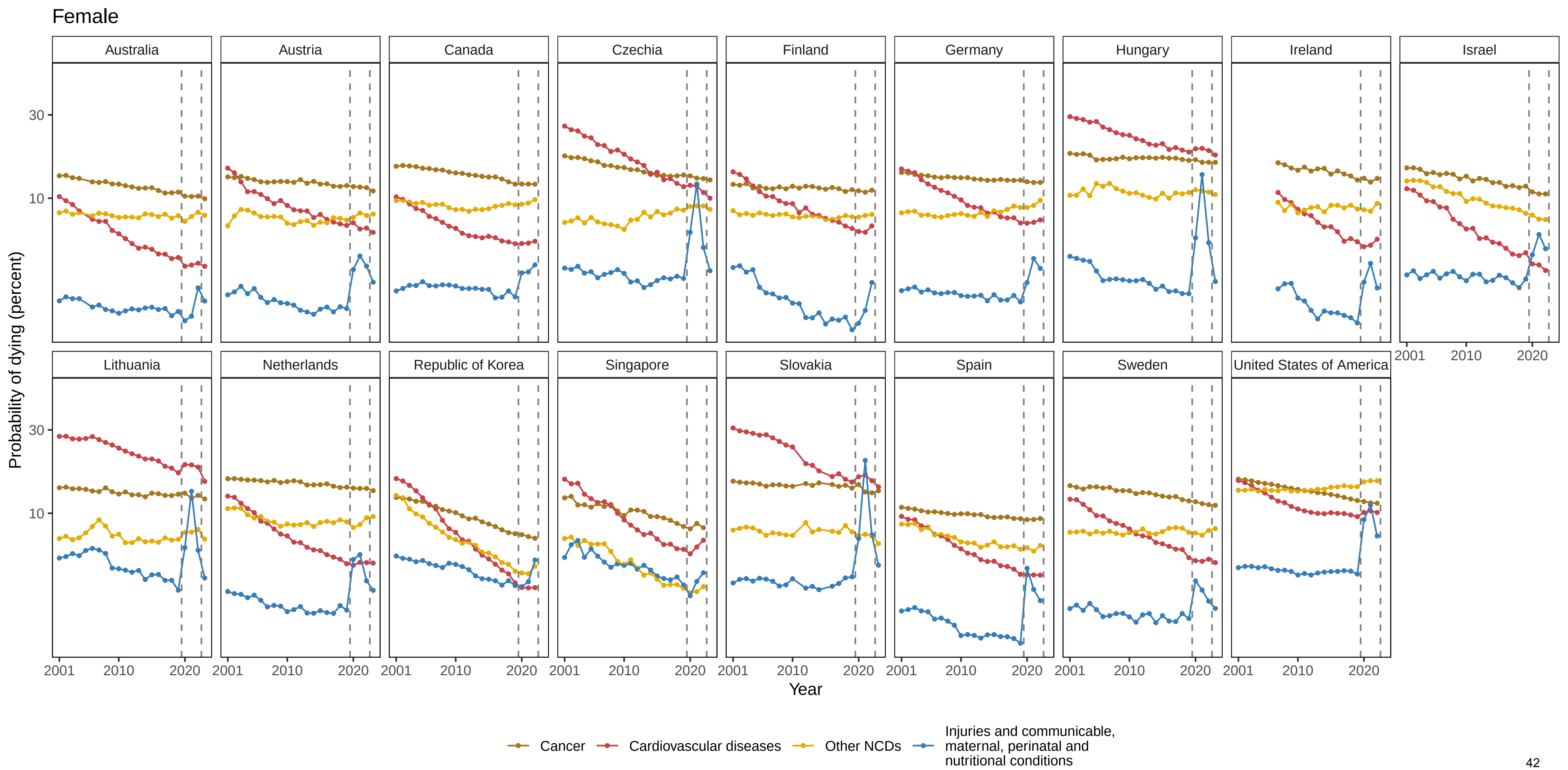
Female
Australia
Austria
Canada
Czechia
Finland
Germany
Hungary
Ireland
Israel
Lithuania
Netherlands
Republic of Korea
Singapore
Slovakia
Spain
Sweden
United States of America
30
10
2001
2010
2020
Probability of dying (percent)
Year
Cancer
Cardiovascular diseases
Other NCDs
Injuries and communicable, maternal, perinatal and nutritional conditions

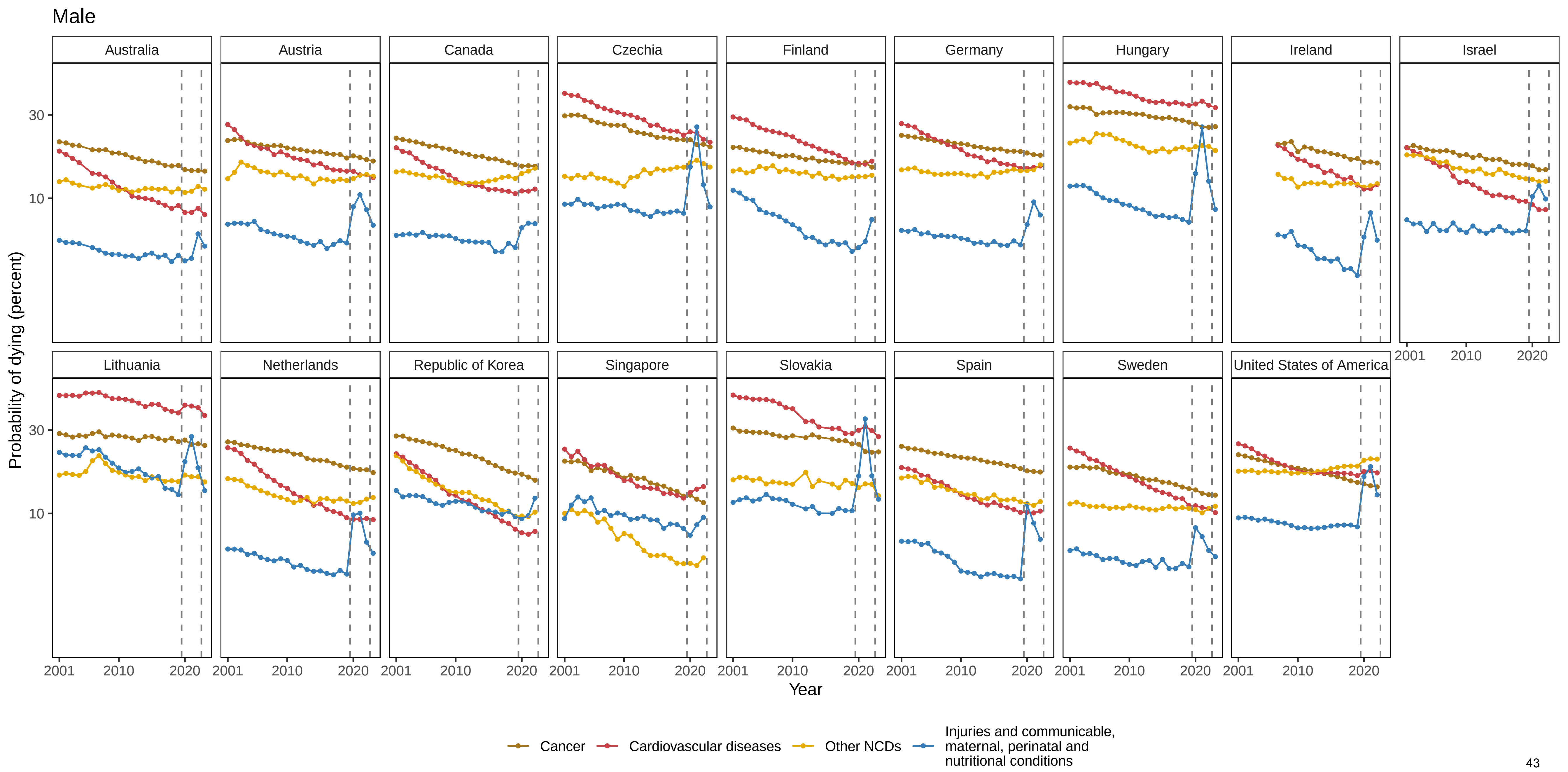
Male
Australia
Austria
Canada
Czechia
Finland
Germany
Hungary
Ireland
Israel
Lithuania
Netherlands
Republic of Korea
Singapore
Slovakia
Spain
Sweden
United States of America
Probability of dying (percent)
30
10
2001
2010
2020
Year
Cancer
Cardiovascular diseases
Other NCDs
Injuries and communicable, maternal, perinatal and nutritional conditions

Appendix Figure 13. Probability of dying from an NCD between birth and 80 years of age in 2001 with uncertainty intervals.

Each point shows the probability of dying from an NCD between birth and 80 years of age for one country and the lines show the 95% uncertainty interval. Estimates are shown for 185 countries and territories, divided into eight reporting regions. Points and lines are coloured by region and labelled with ISO3 codes for each country. Regions are ordered in the same order as Figure 2 of the main paper, and countries are ordered in the ascending order of the probability of death.

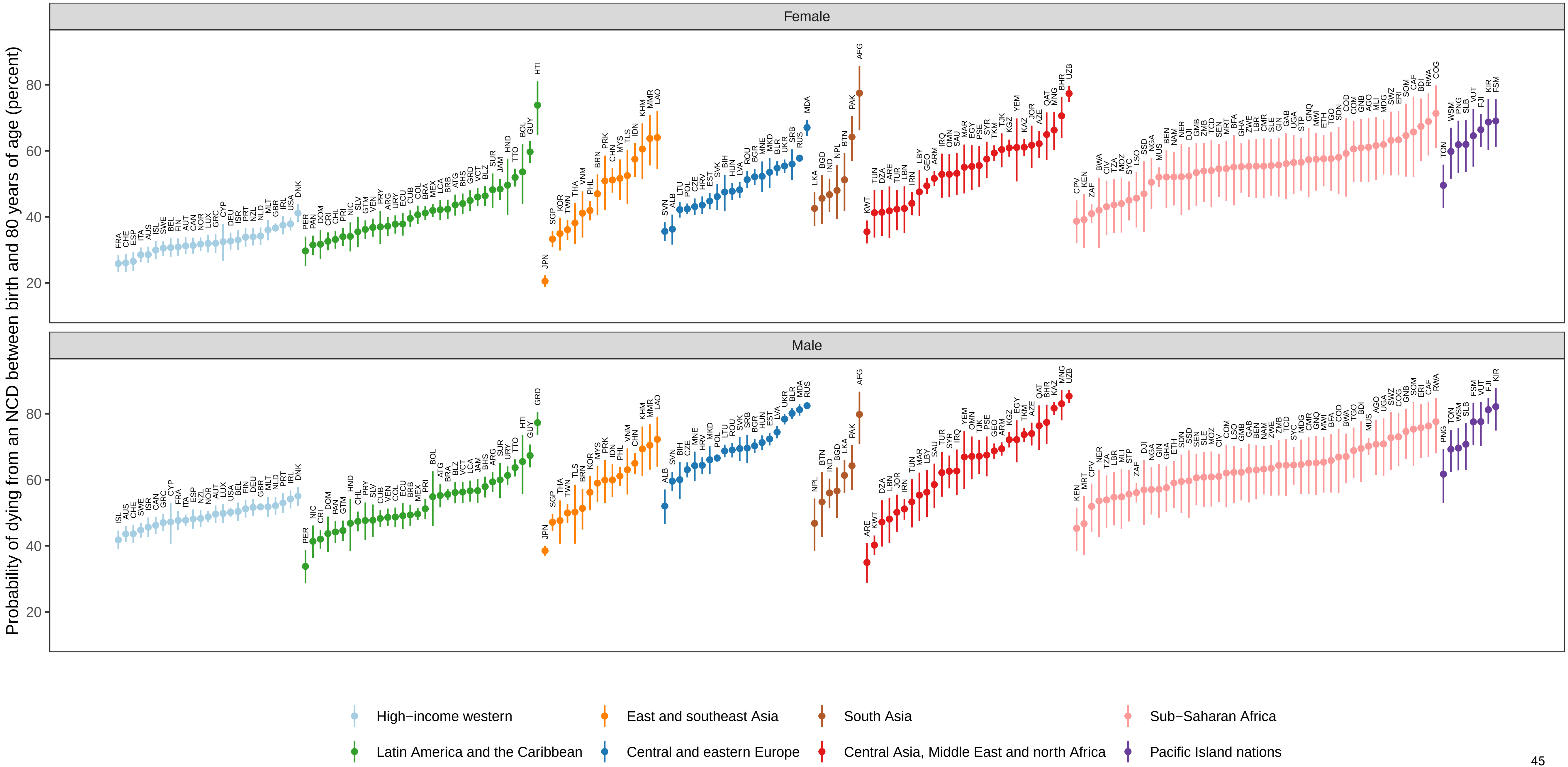

Probability of dying from an NCD between birth and 80 years of age (percent)
Female
Male
20
40
60
80
High−income western
Latin America and the Caribbean
East and southeast Asia
Central and eastern Europe
South Asia
Central Asia, Middle East and north Africa
Sub−Saharan Africa
Pacific Island nations

Appendix Figure 14. Probability of dying from an NCD between birth and 80 years of age in 2010 with uncertainty intervals.

Each point shows the probability of dying from an NCD between birth and 80 years of age for one country and the lines show the 95% uncertainty interval. Estimates are shown for 185 countries and territories, divided into eight reporting regions. Points and lines are coloured by region and labelled with ISO3 codes for each country. Regions are ordered in the same order as Figure 2 of the main paper, and countries are ordered in the ascending order of the probability of death.

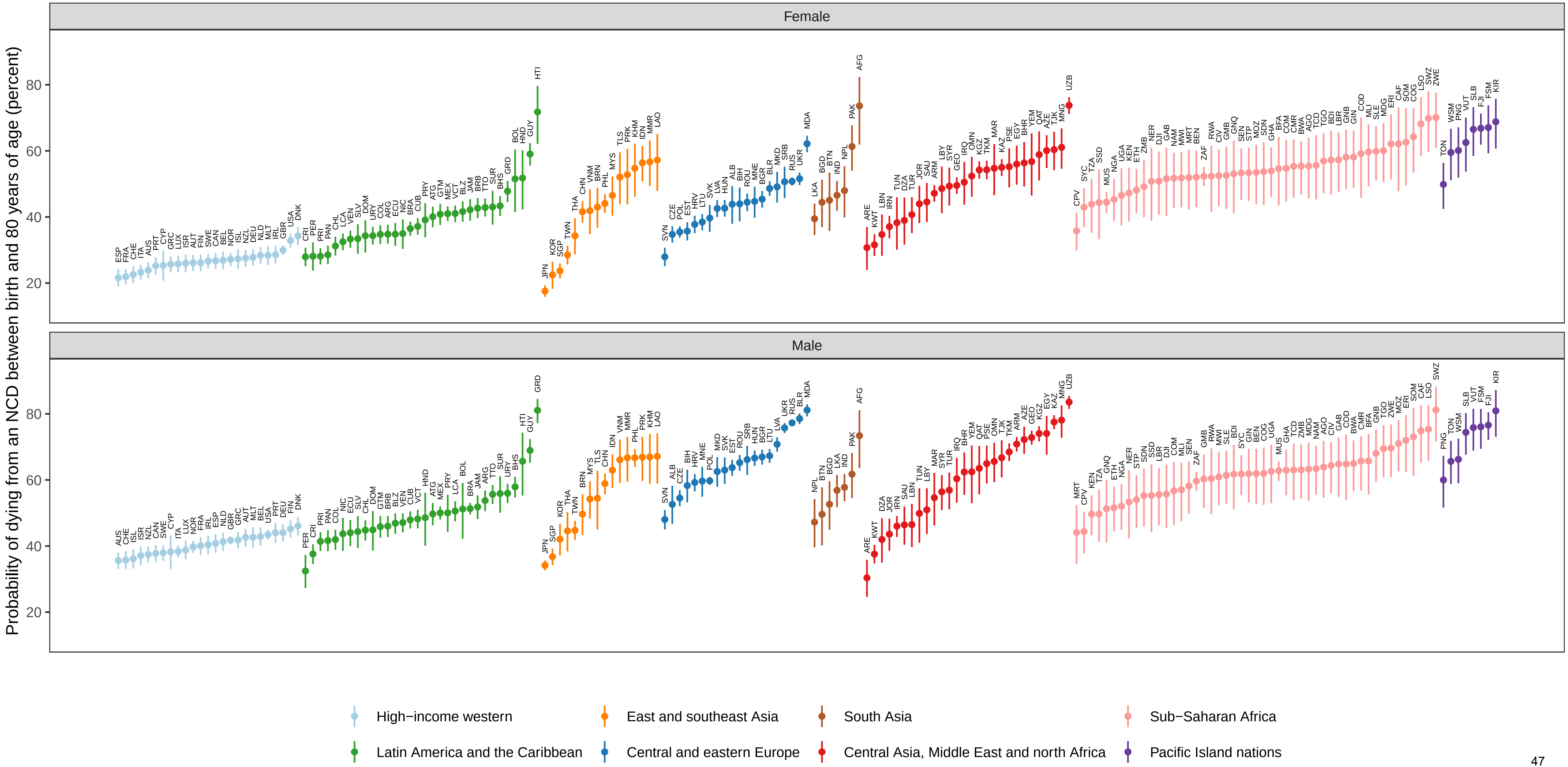

Female
Male
Probability of dying from an NCD between birth and 80 years of age (percent)
20
40
60
80
High−income western
Latin America and the Caribbean
East and southeast Asia
Central and eastern Europe
South Asia
Central Asia, Middle East and north Africa
Sub−Saharan Africa
Pacific Island nations

Appendix Figure 15. Probability of dying from an NCD between birth and 80 years of age in 2019 with uncertainty intervals.

Each point shows the probability of dying from an NCD between birth and 80 years of age for one country and the lines show the 95% uncertainty interval. Estimates are shown for 185 countries and territories, divided into eight reporting regions. Points and lines are coloured by region and labelled with ISO3 codes for each country. Regions are ordered in the same order as Figure 2 of the main paper, and countries are ordered in the ascending order of the probability of death.

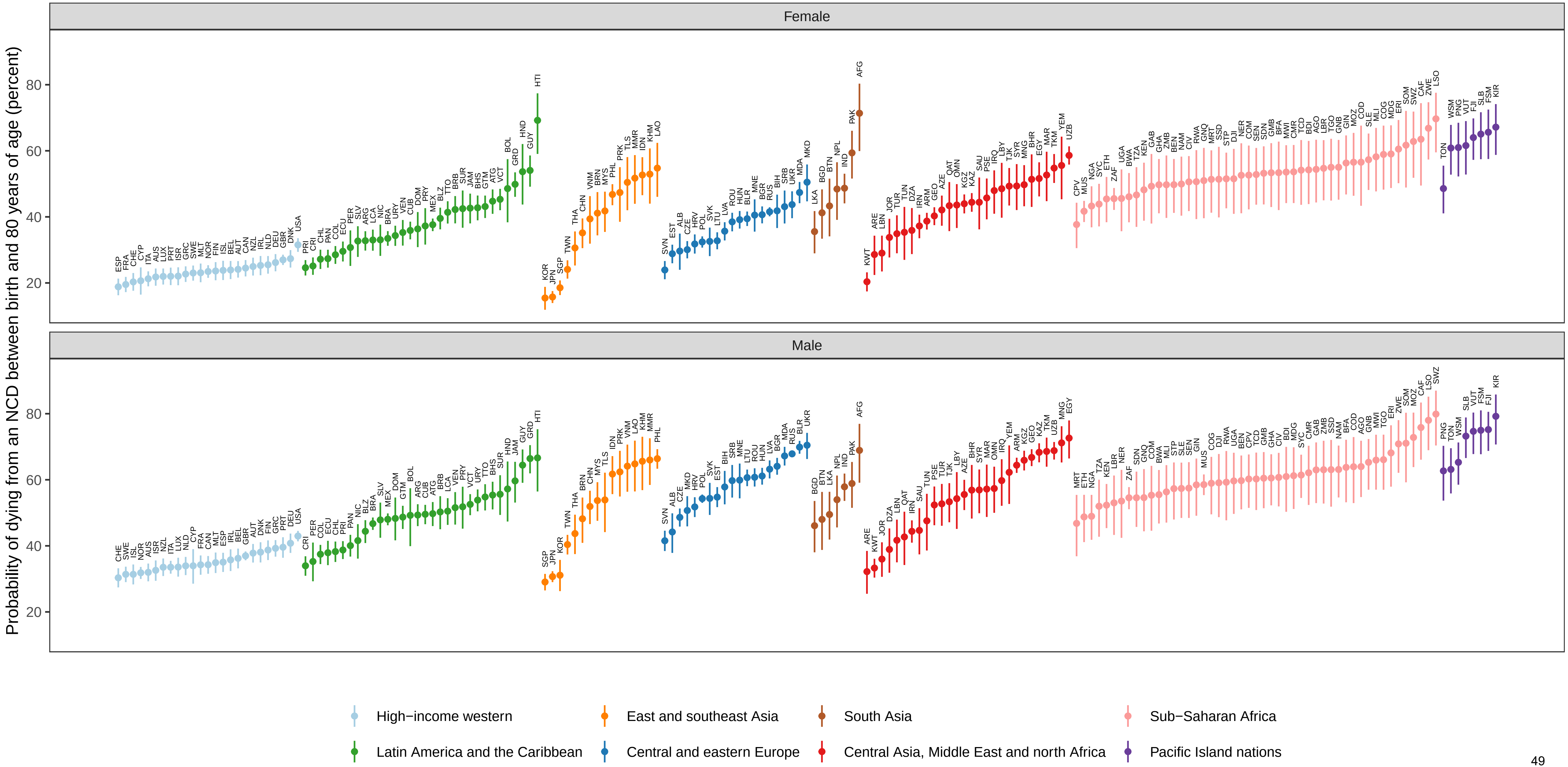

Female
Male
Probability of dying from an NCD between birth and 80 years of age (percent)
20
40
60
80
High−income western
Latin America and the Caribbean
East and southeast Asia
Central and eastern Europe
South Asia
Central Asia, Middle East and north Africa
Sub−Saharan Africa
Pacific Island nations

Appendix Figure 16. Change in the probability of dying from an NCD between birth and 80 years of age from 2001 to 2010 with uncertainty intervals.

Each point shows the change in the probability of dying from an NCD between birth and 80 years of age for one country and the lines show the 95% uncertainty interval. Estimates are shown for 185 countries and territories, divided into eight reporting regions. Points and lines are coloured by region and labelled with ISO3 codes for each country. Regions are ordered in the same order as Figure 2 of the main paper, and countries are ordered in the ascending order of the probability of death.

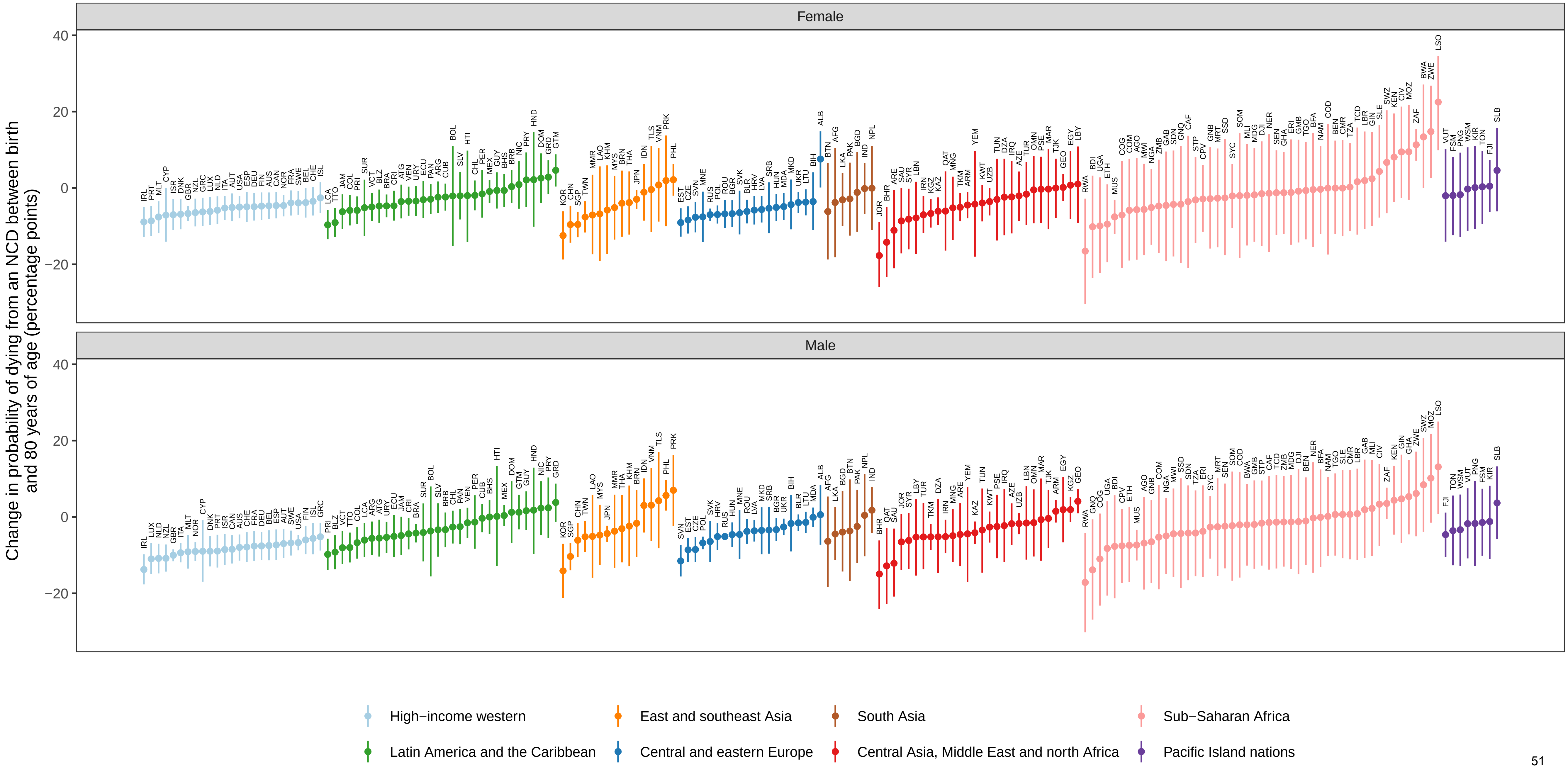
Female
Male
Change in probability of dying from an NCD between birth and 80 years of age (percentage points)
40
20
0
−20
High−income western
Latin America and the Caribbean
East and southeast Asia
Central and eastern Europe
South Asia
Central Asia, Middle East and north Africa
Sub−Saharan Africa
Pacific Island nations

Appendix Figure 17. Change in the probability of dying from an NCD between birth and 80 years of age from 2010 to 2019 with uncertainty intervals.

Each point shows the change in the probability of dying from an NCD between birth and 80 years of age for one country and the lines show the 95% uncertainty interval. Estimates are shown for 185 countries and territories, divided into eight reporting regions. Points and lines are coloured by region and labelled with ISO3 codes for each country. Regions are ordered in the same order as Figure 2 of the main paper, and countries are ordered in the ascending order of the probability of death.

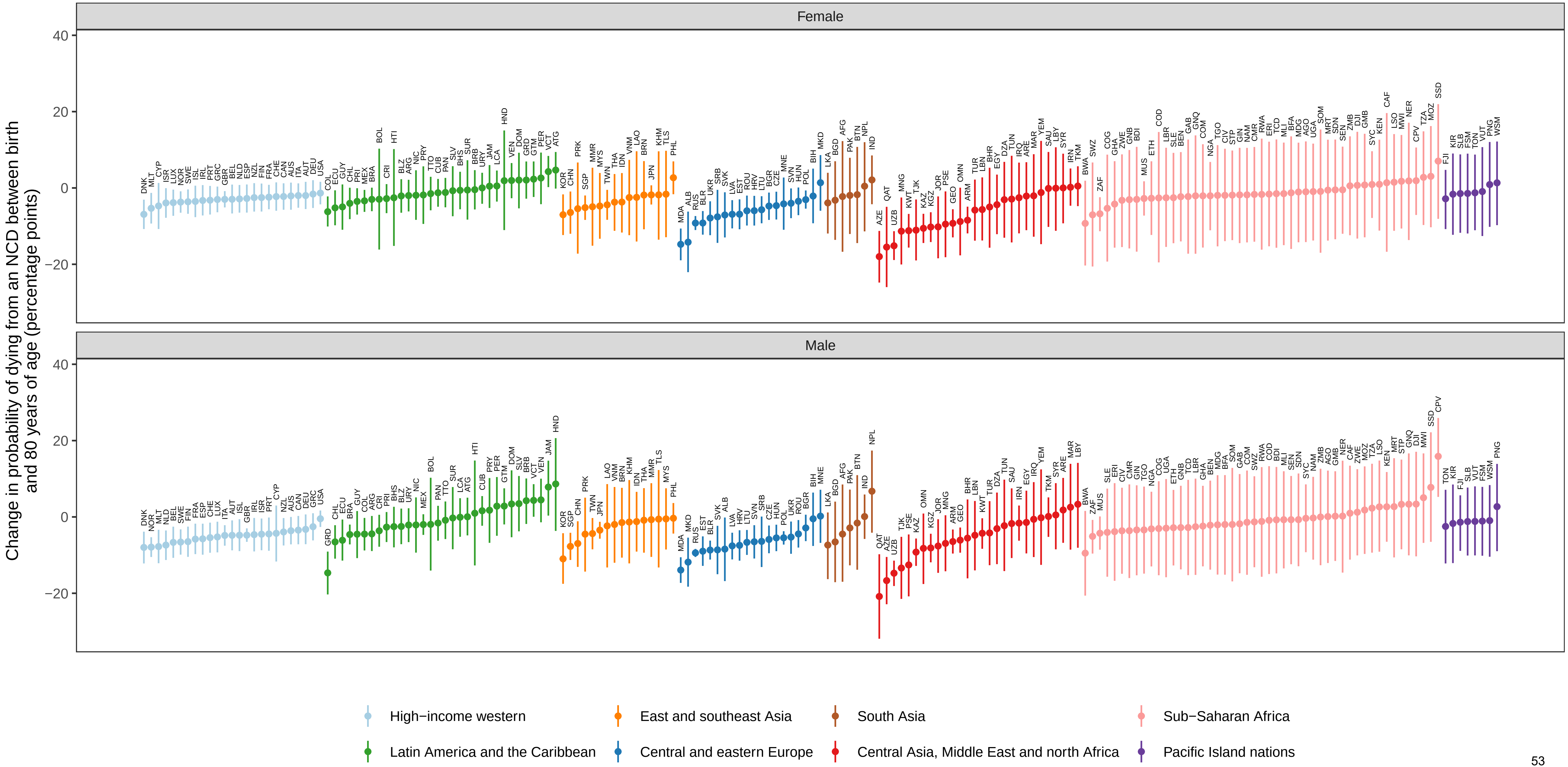
Female
Male
Change in probability of dying from an NCD between birth and 80 years of age (percentage points)
40
20
0
−20
High−income western
Latin America and the Caribbean
East and southeast Asia
Central and eastern Europe
South Asia
Central Asia, Middle East and north Africa
Sub−Saharan Africa
Pacific Island nations

Appendix Figure 18. Probability of dying from NCD4 between 30 and 70 years of age in 2019 and change from 2010 to 2019.

For change in probability of dying from NCD4 between 30 and 70 years of age from 2010 to 2019, green indicates a decline in NCD4 mortality and red indicates an increase. The density plot alongside each map shows the smoothed distribution of estimates across countries.

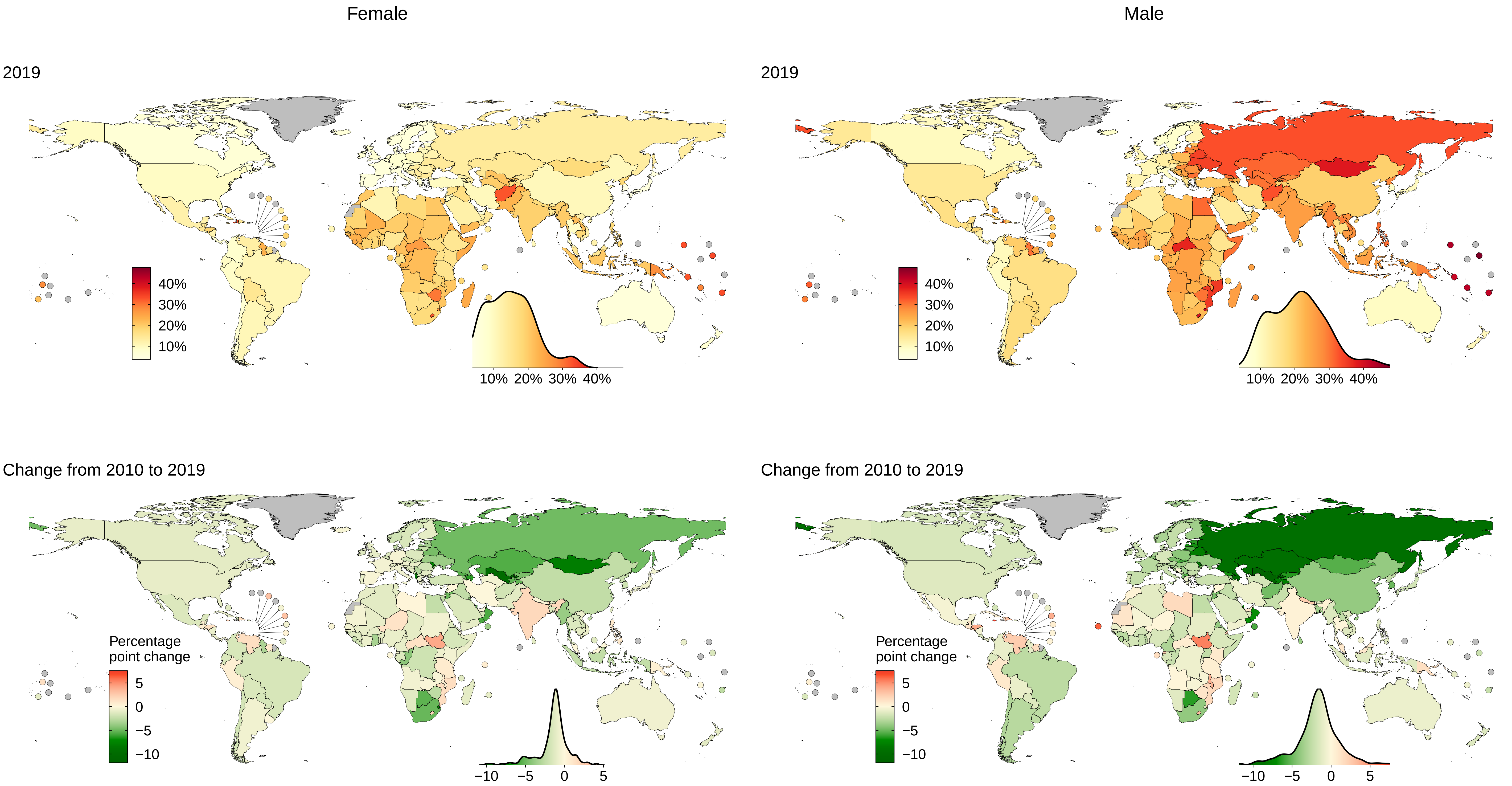
Female
Male
2019
2019
40%
30%
20%
10%
10% 20% 30% 40%
Change from 2010 to 2019
Change from 2010 to 2019
Percentage
point change
5
0
−5
−10
−10 −5 0 5

Appendix Figure 19. Change in NCD4 mortality from 2001 to 2019.

Each line connects the probability of dying between 30 and 70 years of age from NCD4 for 2001, 2010 and 2019 for one country. For each country, the difference in level between consecutive pairs of years represents change over the intervals from 2001 to 2010 and from 2010 to 2019. Data are shown for 185 countries and territories, divided into eight reporting regions. Lines are coloured by region and labelled with ISO3 codes for each country. See Figure 2 caption for ISO3 codes and corresponding country names. The bold black line in each panel connects the mean levels (across countries in that panel, unweighted for population) in 2001, 2010 and 2019. Regions are ordered by increasing mean probability of dying of the countries in each region for females in 2001.

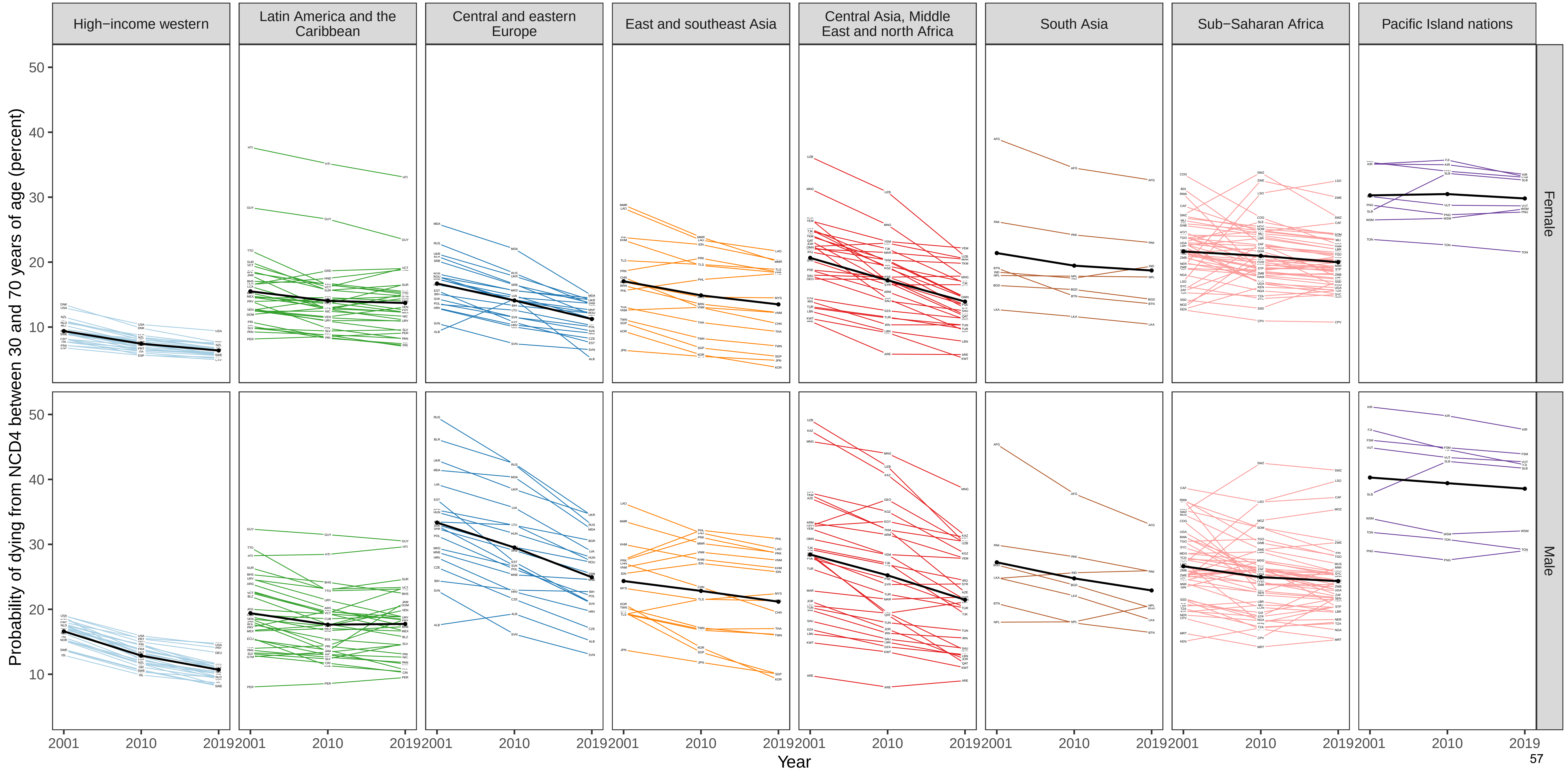
High−income western
Latin America and the Caribbean
Central and eastern Europe
East and southeast Asia
Central Asia, Middle East and north Africa
South Asia
Sub−Saharan Africa
Pacific Island nations
Female
Male
Probability of dying from NCD4 between 30 and 70 years of age (percent)
Year
2001
2010
2019
10
20
30
40
50

Appendix Figure 20. Contributions of mortality from different NCD4 causes of death and in different age groups to overall change in NCD mortality from 2010 to 2019.

Panel A shows the contribution of mutually exclusive, collectively exhaustive NCD4 causes of death to the change in the probability of dying from NCD4 between 30 and 70 years of age from 2010 to 2019. Each column represents a cause of death with causes arranged by NCD4 disease category. **Panel B** shows the contribution of five-year age groups to the change in this probability over the same period, with each column representing a five-year age group.

In both panels, each row represents a country. Results are shown for 63 countries, of which 51 were identified as having high-quality data and 12 were selected based on population size, as detailed in Methods. These 12 countries are denoted with asterisks. Countries are grouped and coloured by region and ordered from the largest decrease to the smallest decrease or largest increase in the probability of dying from NCD4 between 30 and 70 years of age from 2010 to 2019. Each tile shows the absolute contribution of a specific NCD4 cause of death or age group to the total change in this probability for one country. Two colour palettes are used: one for the overall change in NCD4 mortality from 2010 to 2019, and one for contributions of individual NCD4 causes of death or age groups. For overall change, black indicates a decrease, blue an increase, and white no change. For contributions to change, green indicates a contribution to lowering NCD4 mortality, red a contribution to increasing it, and white a contribution of zero.

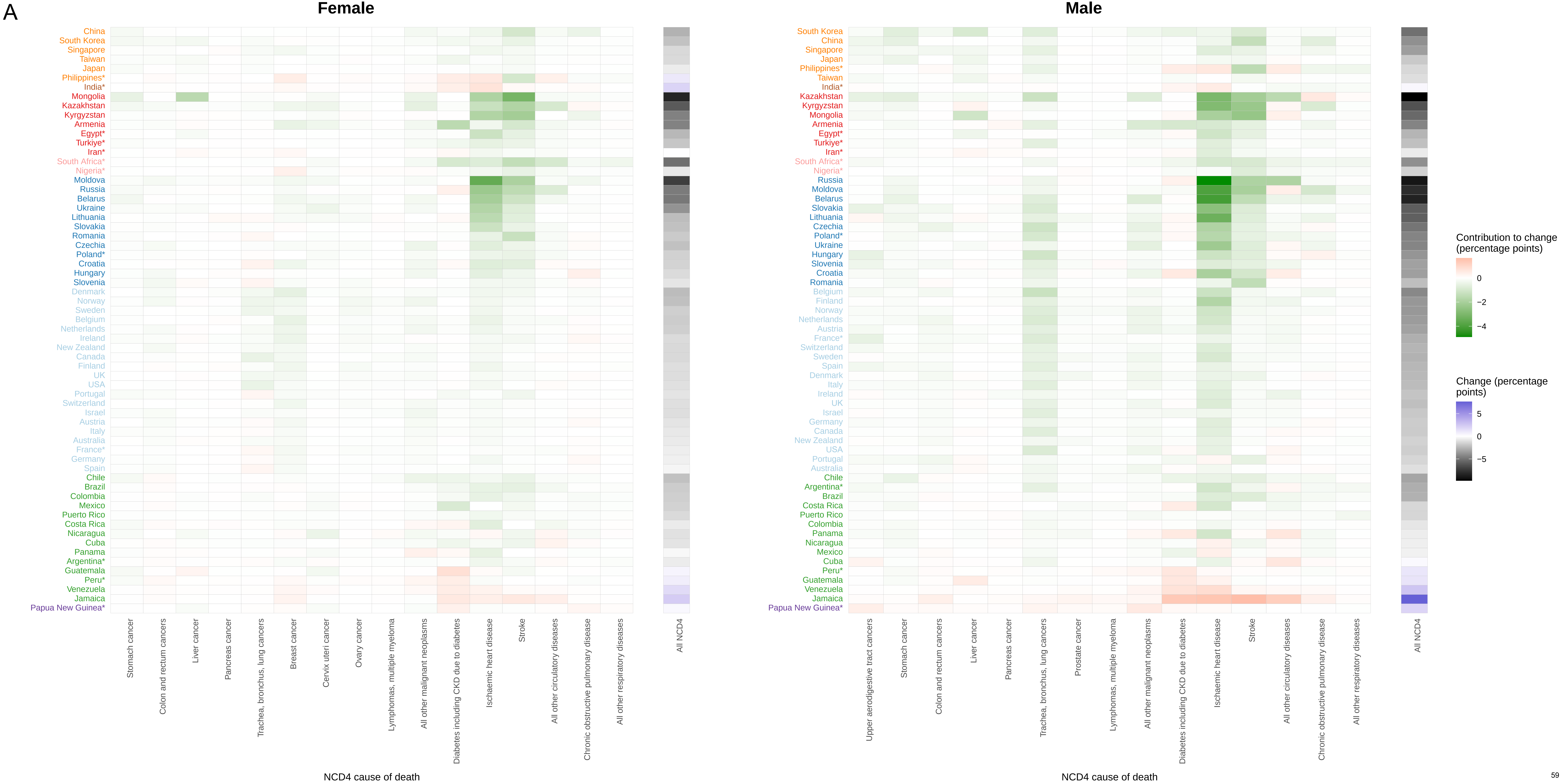
A
Female
Male
China
South Korea
Singapore
Taiwan
Japan
Philippines*
India*
Mongolia
Kazakhstan
Kyrgyzstan
Armenia
Egypt*
Turkiye*
Iran*
South Africa*
Nigeria*
Moldova
Russia
Belarus
Ukraine
Lithuania
Slovakia
Romania
Czechia
Poland*
Croatia
Hungary
Slovenia
Denmark
Norway
Sweden
Belgium
Netherlands
Ireland
New Zealand
Canada
Finland
UK
USA
Portugal
Switzerland
Israel
Austria
Italy
Australia
France*
Germany
Spain
Chile
Brazil
Colombia
Mexico
Puerto Rico
Costa Rica
Nicaragua
Cuba
Panama
Argentina*
Guatemala
Peru*
Venezuela
Jamaica
Papua New Guinea*
Stomach cancer
Colon and rectum cancers
Liver cancer
Pancreas cancer
Trachea, bronchus, lung cancers
Breast cancer
Cervix uteri cancer
Ovary cancer
Lymphomas, multiple myeloma
All other malignant neoplasms
Diabetes including CKD due to diabetes
Ischaemic heart disease
Stroke
All other circulatory diseases
Chronic obstructive pulmonary disease
All other respiratory diseases
All NCD4
NCD4 cause of death
South Korea
China
Singapore
Japan
Philippines*
Taiwan
India*
Kazakhstan
Kyrgyzstan
Mongolia
Armenia
Egypt*
Turkiye*
Iran*
South Africa*
Nigeria*
Russia
Moldova
Belarus
Slovakia
Lithuania
Czechia
Poland*
Ukraine
Hungary
Slovenia
Croatia
Romania
Belgium
Finland
Norway
Netherlands
Austria
France*
Switzerland
Sweden
Spain
Denmark
Italy
Ireland
UK
Israel
Germany
Canada
New Zealand
USA
Portugal
Australia
Chile
Argentina*
Brazil
Costa Rica
Puerto Rico
Colombia
Panama
Nicaragua
Mexico
Cuba
Peru*
Guatemala
Venezuela
Jamaica
Papua New Guinea*
Upper aerodigestive tract cancers
Stomach cancer
Colon and rectum cancers
Liver cancer
Pancreas cancer
Trachea, bronchus, lung cancers
Prostate cancer
Lymphomas, multiple myeloma
All other malignant neoplasms
Diabetes including CKD due to diabetes
Ischaemic heart disease
Stroke
All other circulatory diseases
Chronic obstructive pulmonary disease
All other respiratory diseases
All NCD4
NCD4 cause of death
Contribution to change (percentage points)
0
−2
−4
Change (percentage points)
5
0
−5

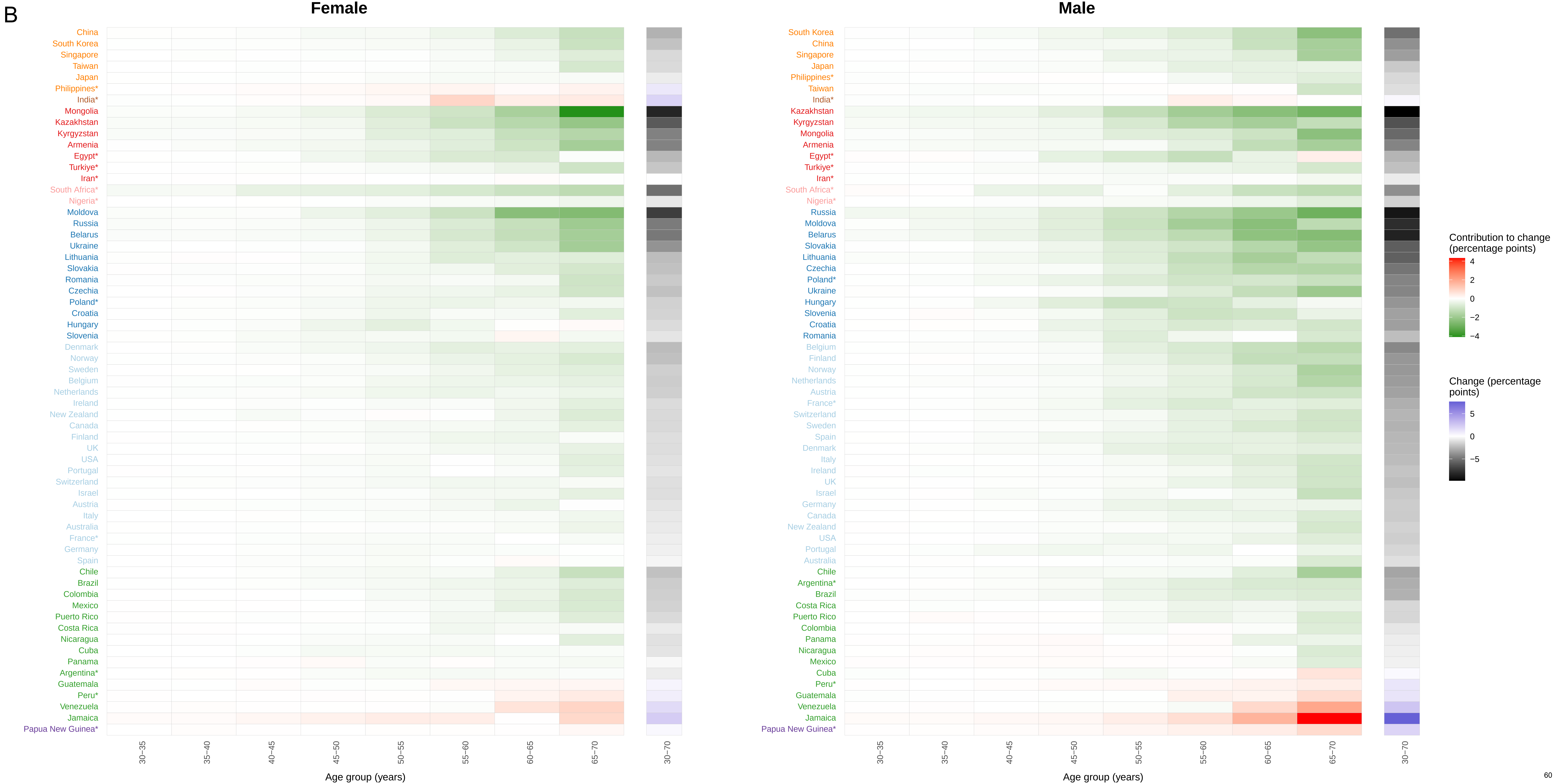

B
Female
Male
China
South Korea
Singapore
Taiwan
Japan
Philippines*
India*
Mongolia
Kazakhstan
Kyrgyzstan
Armenia
Egypt*
Turkiye*
Iran*
South Africa*
Nigeria*
Moldova
Russia
Belarus
Ukraine
Lithuania
Slovakia
Romania
Czechia
Poland*
Croatia
Hungary
Slovenia
Denmark
Norway
Sweden
Belgium
Netherlands
Ireland
New Zealand
Canada
Finland
UK
USA
Portugal
Switzerland
Israel
Austria
Italy
Australia
France*
Germany
Spain
Chile
Brazil
Colombia
Mexico
Puerto Rico
Costa Rica
Nicaragua
Cuba
Panama
Argentina*
Guatemala
Peru*
Venezuela
Jamaica
Papua New Guinea*
30–35
35–40
40–45
45–50
50–55
55–60
60–65
65–70
30–70
Age group (years)
Contribution to change (percentage points)
4
2
0
−2
−4
Change (percentage points)
5
0
−5

Appendix Figure 21. Contributions of mortality from different NCD4 causes of death and in different age groups to slowdown or acceleration of change in NCD4 mortality from 2010 to 2019 compared to change from 2001 to 2010.

Panel A shows the contribution of mutually exclusive, collectively exhaustive NCD4 causes of death to the difference in change in the probability of dying from NCD4 between 30 and 70 years of age between two decades (from 2010 to 2019, and from 2001 to 2010). Each column represents a cause of death with causes arranged by disease category. **Panel B** shows the contribution of five-year age groups to this decadal difference in change, with each column representing a five-year age group.

In both panels, each row represents a country. Results are shown for 63 countries, of which 51 were identified as having high-quality data and 12 were selected based on population size, as detailed in Methods. These 12 countries are denoted with asterisks. Countries are grouped and coloured by region and ordered from the largest improvement in NCD4 mortality from 2010 to 2019 compared to the preceding decade to the largest deterioration. Each tile shows the absolute contribution of a specific NCD4 cause of death or age group to the decadal difference in change in NCD4 mortality between the two decades for one country. Two colour palettes are used: one for the overall decadal difference in change, and one for contributions of individual NCD4 causes of death or age groups. For overall decadal difference in change, yellow indicates improvement (a larger decline, smaller increase or reversal of an increase), magenta deterioration (a smaller decline, reversal of a decline or a larger increase), and white no difference in the magnitude of change. For contributions to decadal difference in change, teal indicates a contribution to improvement of trend (a larger decline, smaller increase or reversal of an increase), brown a contribution to deterioration of trend (a smaller decline, reversal of a decline or a larger increase), and white a contribution of zero.

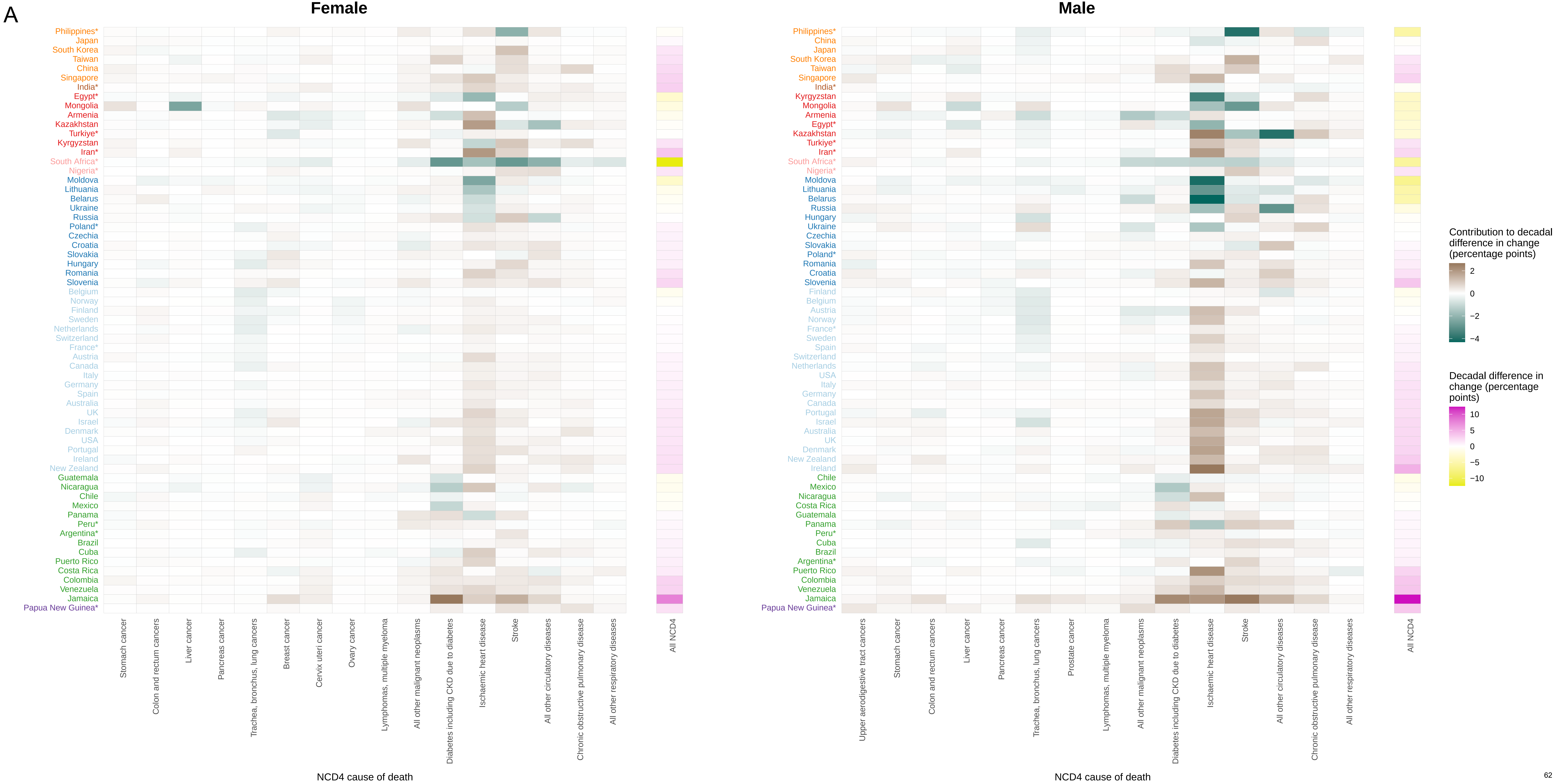
A
Female
Male
NCD4 cause of death
NCD4 cause of death
Stomach cancer
Colon and rectum cancers
Liver cancer
Pancreas cancer
Trachea, bronchus, lung cancers
Breast cancer
Cervix uteri cancer
Ovary cancer
Lymphomas, multiple myeloma
All other malignant neoplasms
Diabetes including CKD due to diabetes
Ischaemic heart disease
Stroke
All other circulatory diseases
Chronic obstructive pulmonary disease
All other respiratory diseases
All NCD4
Upper aerodigestive tract cancers
Prostate cancer
Contribution to decadal difference in change (percentage points)
2
0
−2
−4
Decadal difference in change (percentage points)
10
5
0
−5
−10

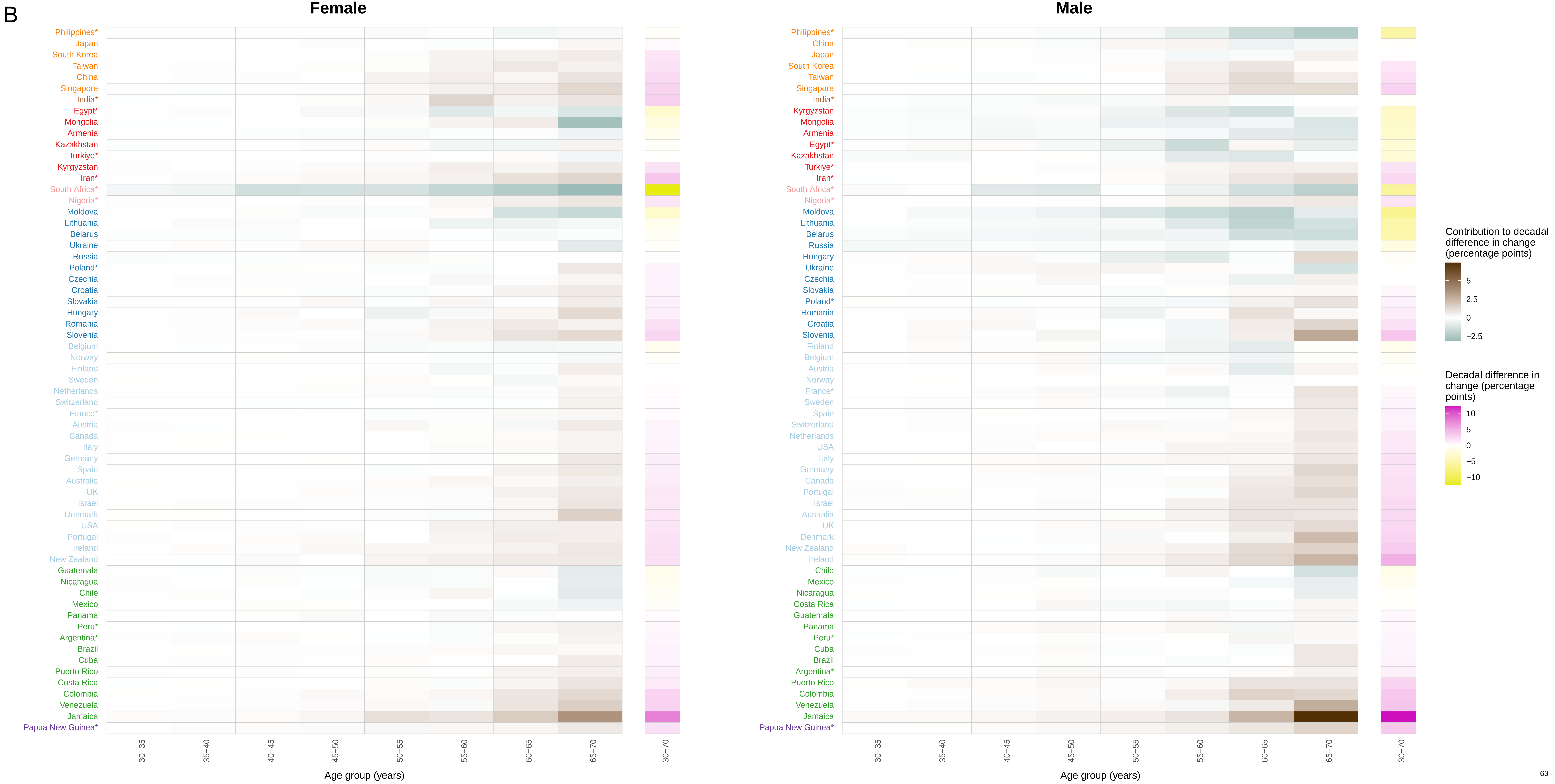
B
Female
Male
Philippines*
Japan
South Korea
Taiwan
China
Singapore
India*
Egypt*
Mongolia
Armenia
Kazakhstan
Turkiye*
Kyrgyzstan
Iran*
South Africa*
Nigeria*
Moldova
Lithuania
Belarus
Ukraine
Russia
Poland*
Czechia
Croatia
Slovakia
Hungary
Romania
Slovenia
Belgium
Norway
Finland
Sweden
Netherlands
Switzerland
France*
Austria
Canada
Italy
Germany
Spain
Australia
UK
Israel
Denmark
USA
Portugal
Ireland
New Zealand
Guatemala
Nicaragua
Chile
Mexico
Panama
Peru*
Argentina*
Brazil
Cuba
Puerto Rico
Costa Rica
Colombia
Venezuela
Jamaica
Papua New Guinea*
30–35
35–40
40–45
45–50
50–55
55–60
60–65
65–70
30–70
Age group (years)
Contribution to decadal difference in change (percentage points)
5
2.5
0
−2.5
Decadal difference in change (percentage points)
10
5
0
−5
−10

Appendix Figure 22. Contributions of mortality from different NCD4 causes of death and in different age groups to how much NCD4 mortality in each country lags its regional benchmark.

Panel A shows the contribution of mutually exclusive, collectively exhaustive NCD4 causes of death to the difference in the change in the probability of dying from NCD4 between 30 and 70 years of age from 2010 to 2019, relative to a country benchmark within each region. Benchmarks are identified as the country in each region with the largest reduction in NCD4 mortality over this period. Each column represents a cause of death with causes arranged by disease category. **Panel B** shows the contribution of five-year age groups to this difference, with each column representing a five-year age group.

In both panels, each row represents a country. Results are shown for 51 countries identified as having high-quality data, as detailed in Methods. Countries are grouped and coloured by region and ordered from the largest decrease to the smallest decrease or largest increase in the probability of dying from NCD4 between 30 and 70 years of age from 2010 to 2019. The benchmark for each region is the country in the first row of its region grouping and is shown in bold font. Each tile shows the absolute contribution of a specific NCD4 cause of death or age group to the difference in change relative to the benchmark country for one country. Two colour palettes are used: one for the overall difference in change relative to the benchmark, and one for contributions of individual NCD4 causes of death or age groups relative to those of the benchmark. For overall difference in change, black indicates a decrease relative to the benchmark, blue an increase relative to the benchmark, and white no difference in change. For contributions to difference in change, green indicates a contribution towards a larger decline or smaller increase relative to the benchmark, red a contribution towards a smaller decline or larger increase relative to the benchmark, and white a contribution of zero.

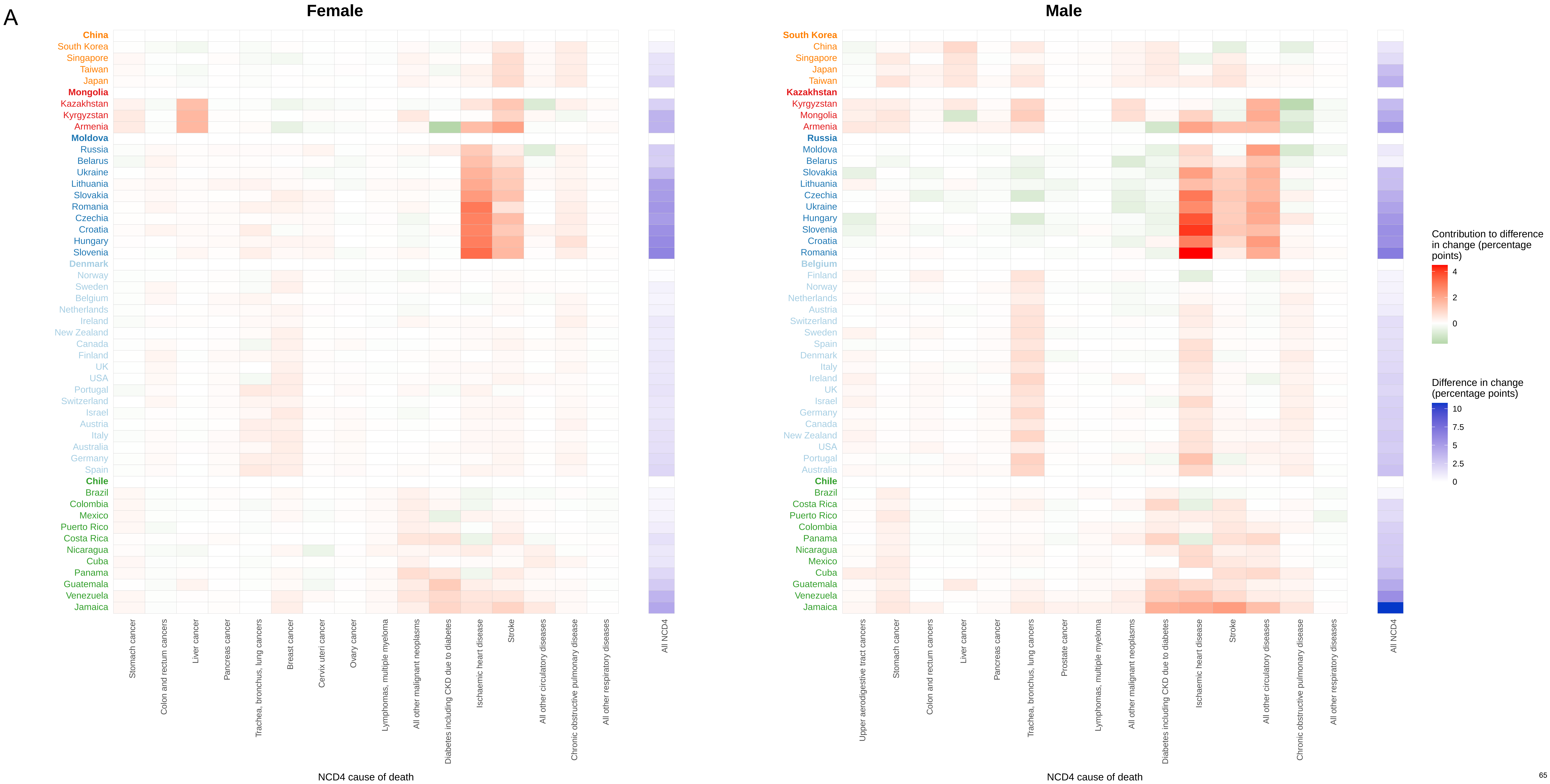

A
Female
Male
China
South Korea
Singapore
Taiwan
Japan
Mongolia
Kazakhstan
Kyrgyzstan
Armenia
Moldova
Russia
Belarus
Ukraine
Lithuania
Slovakia
Romania
Czechia
Croatia
Hungary
Slovenia
Denmark
Norway
Sweden
Belgium
Netherlands
Ireland
New Zealand
Canada
Finland
UK
USA
Portugal
Switzerland
Israel
Austria
Italy
Australia
Germany
Spain
Chile
Brazil
Colombia
Mexico
Puerto Rico
Costa Rica
Nicaragua
Cuba
Panama
Guatemala
Venezuela
Jamaica
Stomach cancer
Colon and rectum cancers
Liver cancer
Pancreas cancer
Trachea, bronchus, lung cancers
Breast cancer
Cervix uteri cancer
Ovary cancer
Lymphomas, multiple myeloma
All other malignant neoplasms
Diabetes including CKD due to diabetes
Ischaemic heart disease
Stroke
All other circulatory diseases
Chronic obstructive pulmonary disease
All other respiratory diseases
All NCD4
NCD4 cause of death
South Korea
China
Singapore
Japan
Taiwan
Kazakhstan
Kyrgyzstan
Mongolia
Armenia
Russia
Moldova
Belarus
Slovakia
Lithuania
Czechia
Ukraine
Hungary
Slovenia
Croatia
Romania
Belgium
Finland
Norway
Netherlands
Austria
Switzerland
Sweden
Spain
Denmark
Italy
Ireland
UK
Israel
Germany
Canada
New Zealand
USA
Portugal
Australia
Chile
Brazil
Costa Rica
Puerto Rico
Colombia
Panama
Nicaragua
Mexico
Cuba
Guatemala
Venezuela
Jamaica
Upper aerodigestive tract cancers
Stomach cancer
Colon and rectum cancers
Liver cancer
Pancreas cancer
Trachea, bronchus, lung cancers
Prostate cancer
Lymphomas, multiple myeloma
All other malignant neoplasms
Diabetes including CKD due to diabetes
Ischaemic heart disease
Stroke
All other circulatory diseases
Chronic obstructive pulmonary disease
All other respiratory diseases
All NCD4
NCD4 cause of death
Contribution to difference in change (percentage points)
4
2
0
Difference in change (percentage points)
10
7.5
5
2.5
0

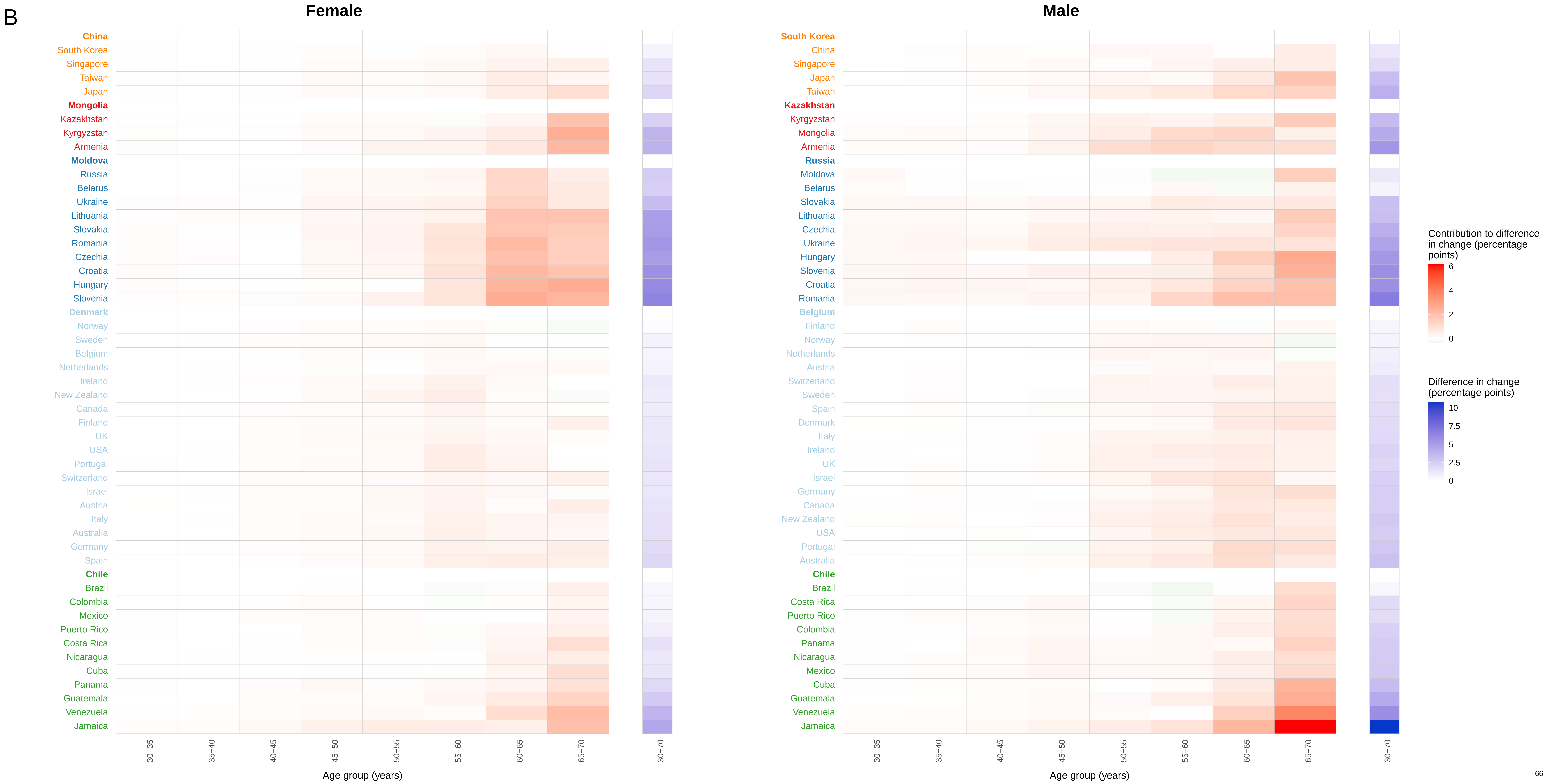
B
Female
Male
China
South Korea
Singapore
Taiwan
Japan
Mongolia
Kazakhstan
Kyrgyzstan
Armenia
Moldova
Russia
Belarus
Ukraine
Lithuania
Slovakia
Romania
Czechia
Croatia
Hungary
Slovenia
Denmark
Norway
Sweden
Belgium
Netherlands
Ireland
New Zealand
Canada
Finland
UK
USA
Portugal
Switzerland
Israel
Austria
Italy
Australia
Germany
Spain
Chile
Brazil
Colombia
Mexico
Puerto Rico
Costa Rica
Nicaragua
Cuba
Panama
Guatemala
Venezuela
Jamaica
South Korea
China
Singapore
Japan
Taiwan
Kazakhstan
Kyrgyzstan
Mongolia
Armenia
Russia
Moldova
Belarus
Slovakia
Lithuania
Czechia
Ukraine
Hungary
Slovenia
Croatia
Romania
Belgium
Finland
Norway
Netherlands
Austria
Switzerland
Sweden
Spain
Denmark
Italy
Ireland
UK
Israel
Germany
Canada
New Zealand
USA
Portugal
Australia
Chile
Brazil
Costa Rica
Puerto Rico
Colombia
Panama
Nicaragua
Mexico
Cuba
Guatemala
Venezuela
Jamaica
30–35
35–40
40–45
45–50
50–55
55–60
60–65
65–70
30–70
Age group (years)
Contribution to difference in change (percentage points)
6
4
2
0
Difference in change (percentage points)
10
7.5
5
2.5
0

Appendix Figure 23. Summary of how direction and size of change in NCD mortality differs when NCD4 between 30 and 70 years is used in place of all NCDs between birth and 80 years.

The Sankey diagrams summarise differences in the number of countries experiencing various directions and sizes of decadal change for two mortality metrics: the probability of dying from any NCD between birth and 80 years of age and the probability of dying from NCD4 between 30 and 70 years of age. Countries are divided into six categories based on direction and size of change over two timeframes (from 2001 to 2010 and from 2010 to 2019). The categories are the same as those in Figures 1–4 and Appendix Table 3.

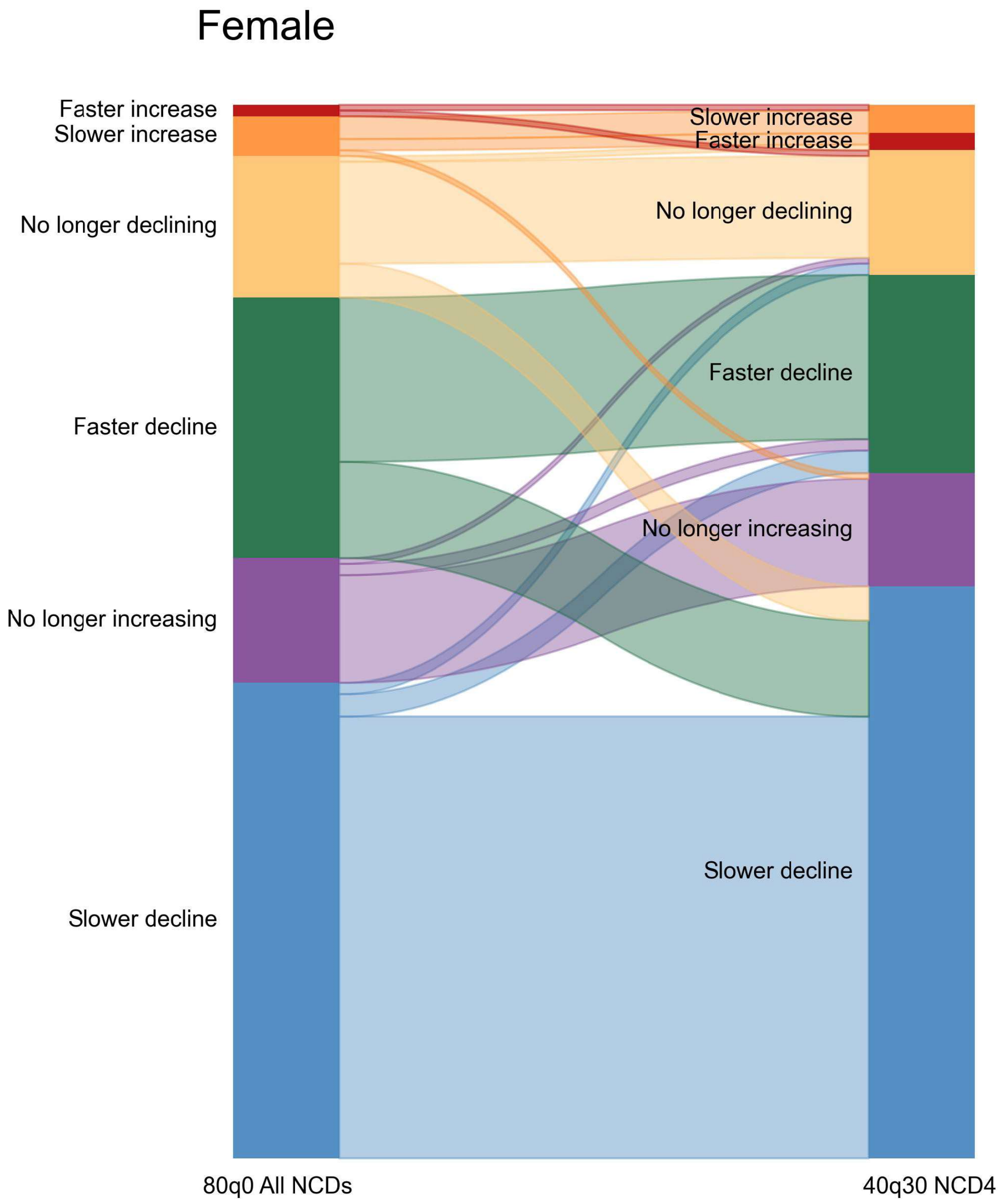
Female
Faster increase
Slower increase
No longer declining
Faster decline
No longer increasing
Slower decline
Slower increase
Faster increase
No longer declining
Faster decline
No longer increasing
Slower decline
80q0 All NCDs
40q30 NCD4

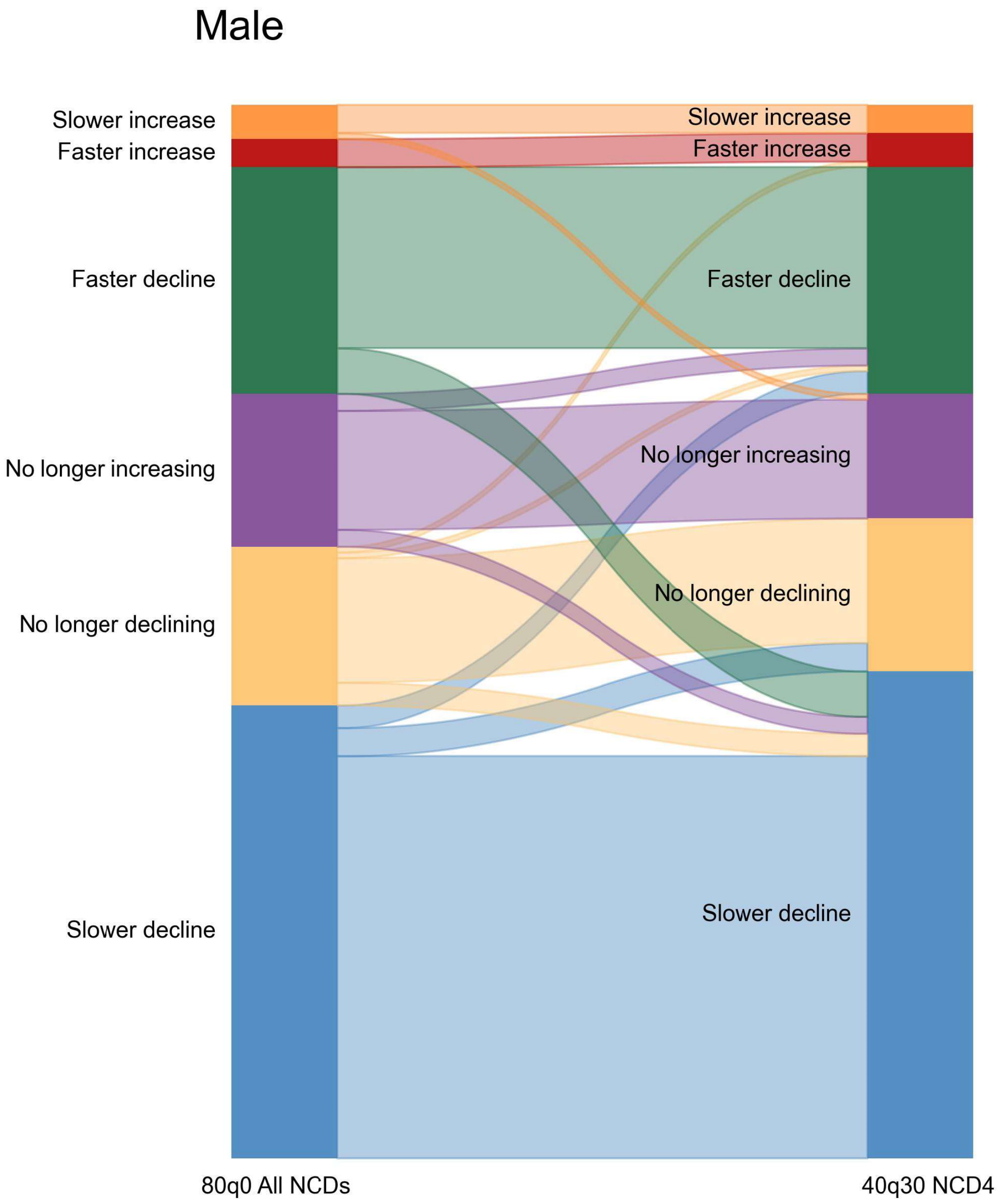
Male
Slower increase
Faster increase
Faster decline
No longer increasing
No longer declining
Slower decline
Slower increase
Faster increase
Faster decline
No longer increasing
No longer declining
Slower decline
80q0 All NCDs
40q30 NCD4

Appendix Figure 24. Change in NCD4 mortality from 2010 to 2019 in relation to the level of mortality in 2010.

Each point represents one country, coloured by region. Data are shown for 185 countries in eight regions.

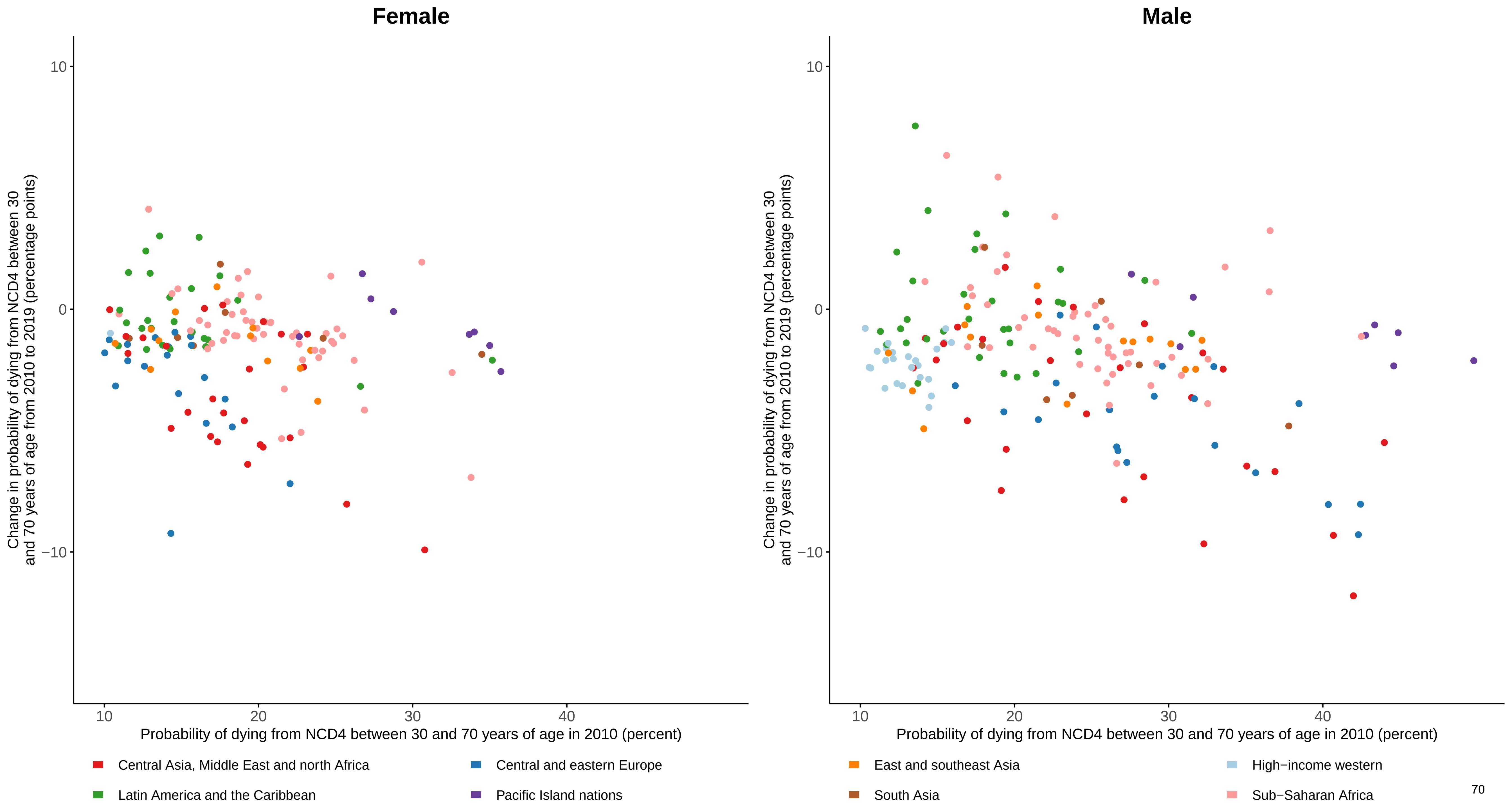
Female
Male
Change in probability of dying from NCD4 between 30 and 70 years of age from 2010 to 2019 (percentage points)
10
0
−10
Probability of dying from NCD4 between 30 and 70 years of age in 2010 (percent)
10
20
30
40
Central Asia, Middle East and north Africa
Central and eastern Europe
East and southeast Asia
High−income western
Latin America and the Caribbean
Pacific Island nations
South Asia
Sub−Saharan Africa

Appendix Figure 25. Change in NCD4 mortality from 2010 to 2019 in females and males.

Each point represents one country, coloured by region. Data are shown for 185 countries in eight regions. The dotted diagonal line represents equal change from 2010 to 2019 in the probability of death from NCD4 between 30 and 70 years of age for females and males. Segments of the plot are labelled to indicate the various relationships between the two sexes in the direction and size of change.

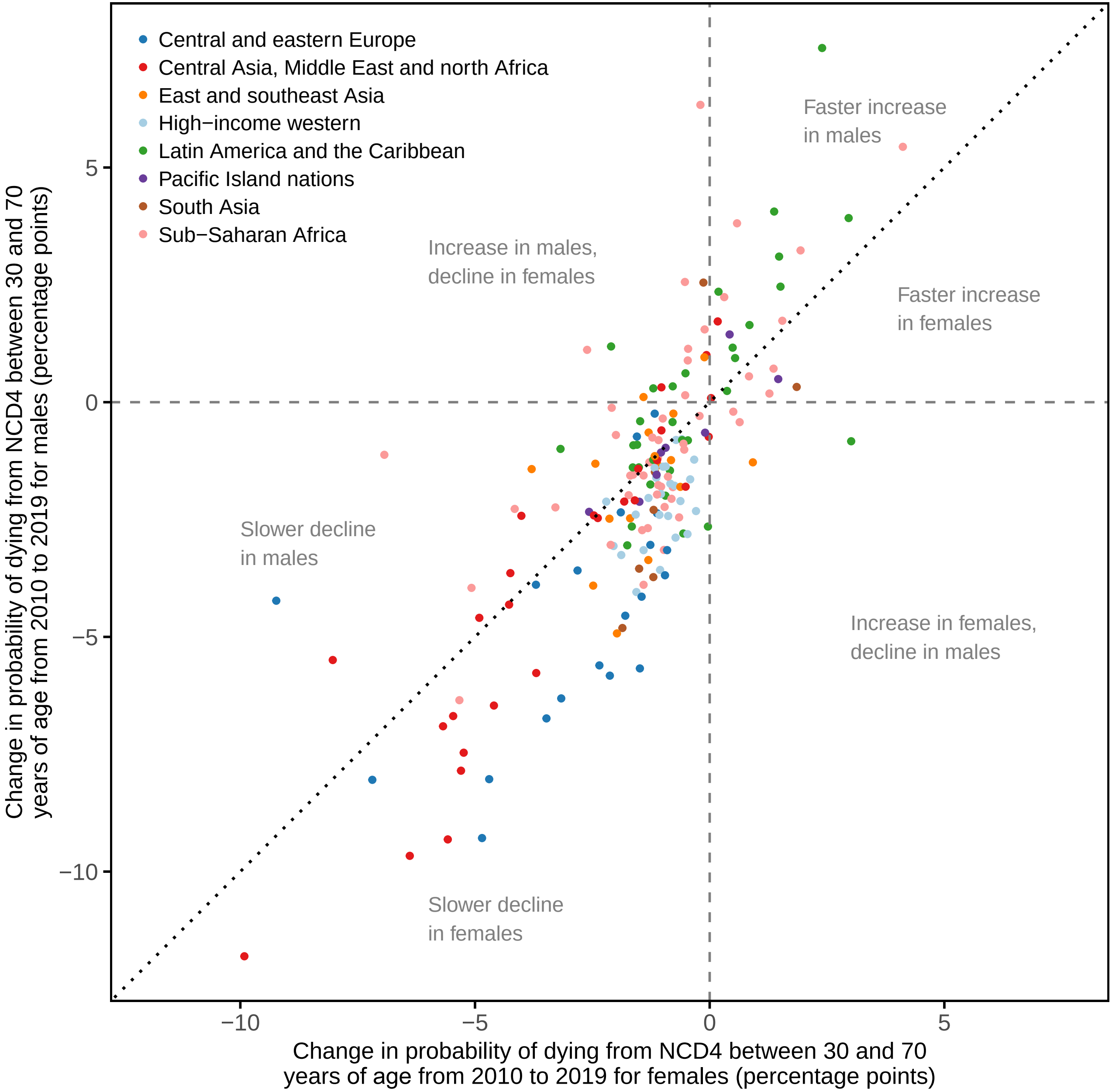

Central and eastern Europe
Central Asia, Middle East and north Africa
East and southeast Asia
High−income western
Latin America and the Caribbean
Pacific Island nations
South Asia
Sub−Saharan Africa
Faster increase in males
Increase in males, decline in females
Faster increase in females
Slower decline in males
Increase in females, decline in males
Slower decline in females
5
0
−5
−10
Change in probability of dying from NCD4 between 30 and 70 years of age from 2010 to 2019 for males (percentage points)
−10
−5
0
5
Change in probability of dying from NCD4 between 30 and 70 years of age from 2010 to 2019 for females (percentage points)

Appendix Figure 26. Age-specific death rates from NCD4 in 2001, 2010 and 2019.

Each line represents the death rate from NCD4 by five-year age group for one year. Death rates are shown for the years 2001, 2010, and 2019 for 63 countries, including 51 with high-quality data and 12 selected based on population size and medium data quality, as detailed in Methods. Death rates are displayed on the log scale.

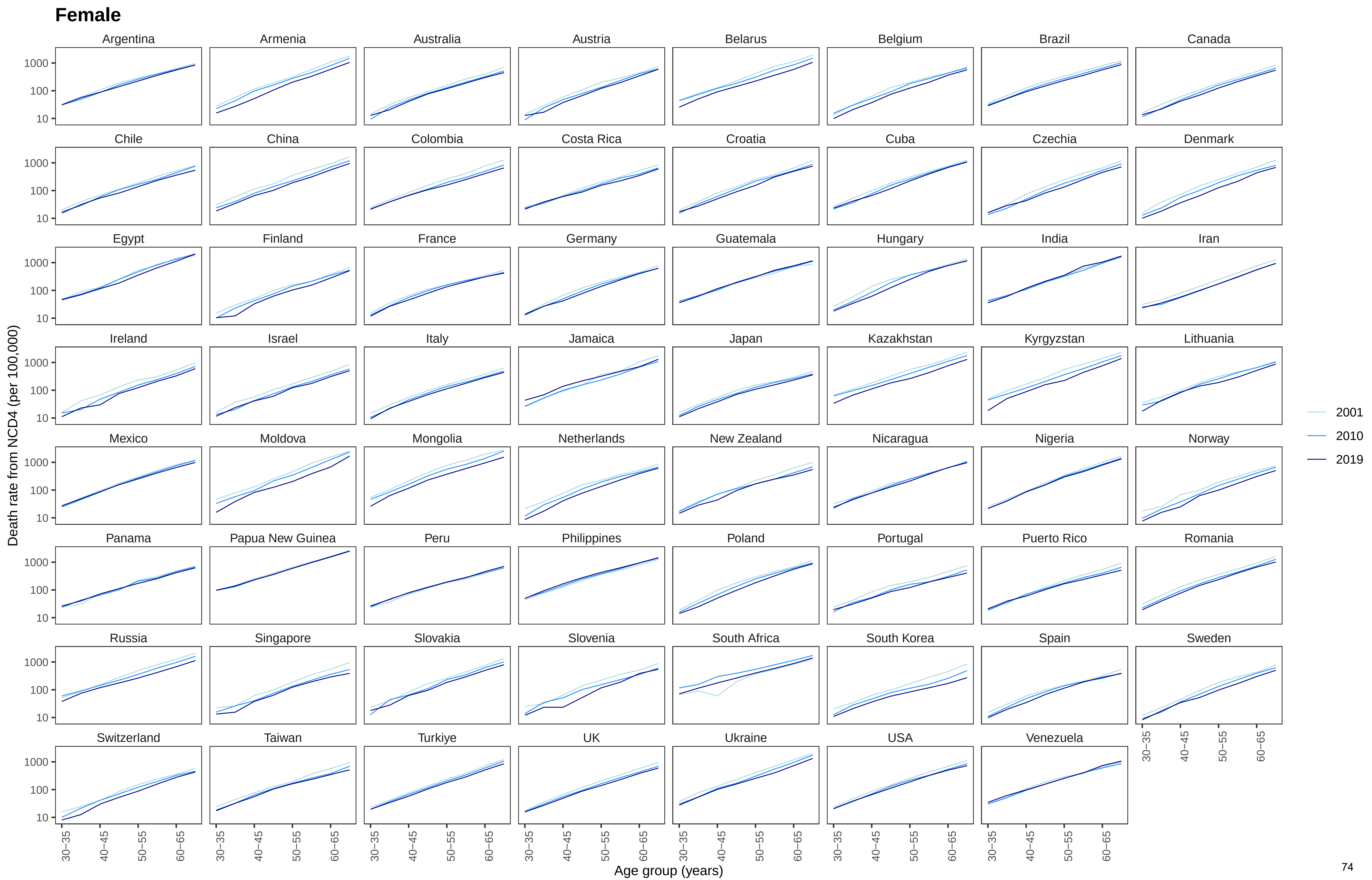
Female
Argentina
Armenia
Australia
Austria
Belarus
Belgium
Brazil
Canada
Chile
China
Colombia
Costa Rica
Croatia
Cuba
Czechia
Denmark
Egypt
Finland
France
Germany
Guatemala
Hungary
India
Iran
Ireland
Israel
Italy
Jamaica
Japan
Kazakhstan
Kyrgyzstan
Lithuania
Mexico
Moldova
Mongolia
Netherlands
New Zealand
Nicaragua
Nigeria
Norway
Panama
Papua New Guinea
Peru
Philippines
Poland
Portugal
Puerto Rico
Romania
Russia
Singapore
Slovakia
Slovenia
South Africa
South Korea
Spain
Sweden
Switzerland
Taiwan
Turkiye
UK
Ukraine
USA
Venezuela
Death rate from NCD4 (per 100,000)
1000
100
10
30–35
40–45
50–55
60–65
Age group (years)
2001
2010
2019

Male

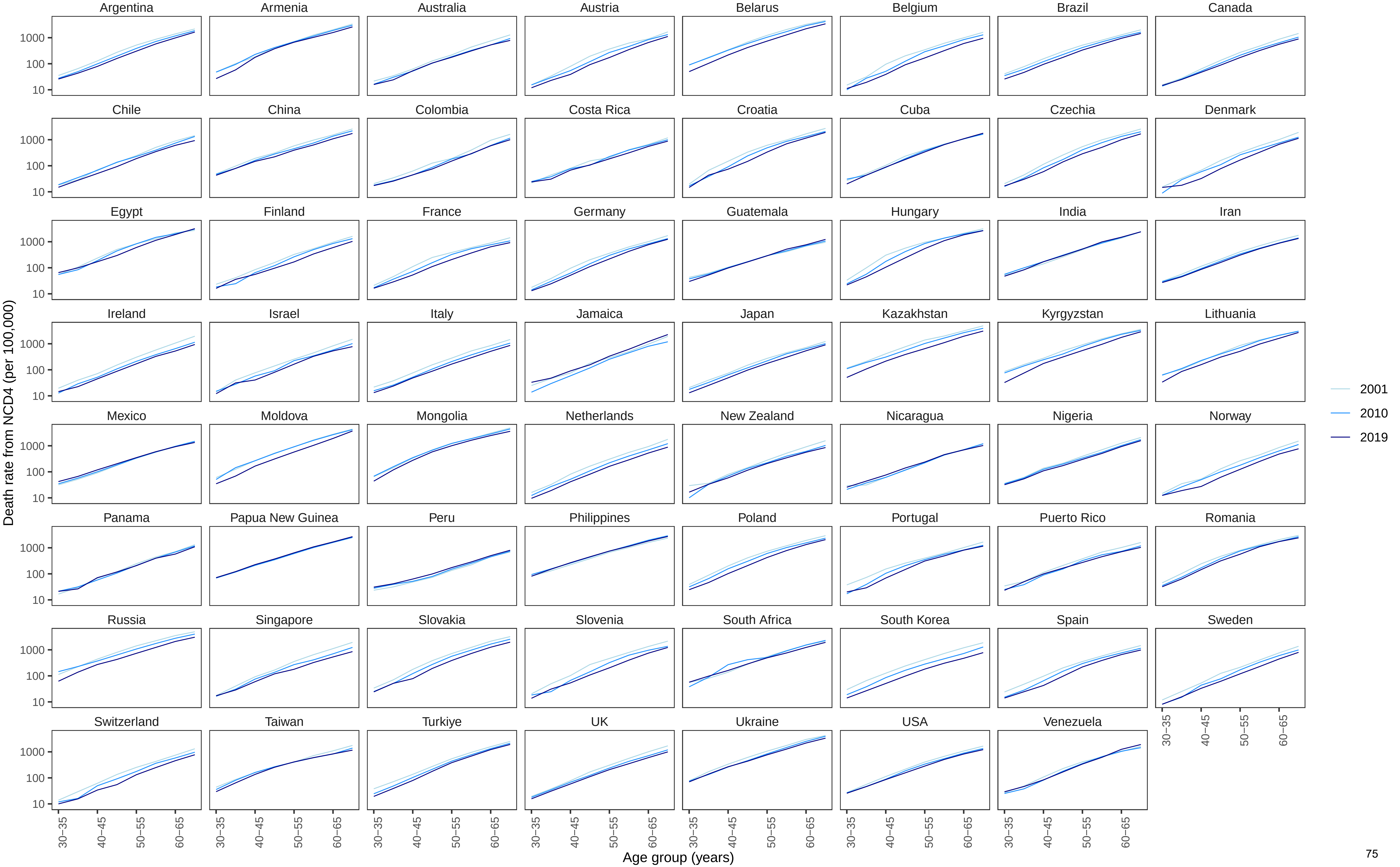
Argentina
Armenia
Australia
Austria
Belarus
Belgium
Brazil
Canada
Chile
China
Colombia
Costa Rica
Croatia
Cuba
Czechia
Denmark
Egypt
Finland
France
Germany
Guatemala
Hungary
India
Iran
Ireland
Israel
Italy
Jamaica
Japan
Kazakhstan
Kyrgyzstan
Lithuania
Mexico
Moldova
Mongolia
Netherlands
New Zealand
Nicaragua
Nigeria
Norway
Panama
Papua New Guinea
Peru
Philippines
Poland
Portugal
Puerto Rico
Romania
Russia
Singapore
Slovakia
Slovenia
South Africa
South Korea
Spain
Sweden
Switzerland
Taiwan
Turkiye
UK
Ukraine
USA
Venezuela
1000
100
10
30–35
40–45
50–55
60–65
Death rate from NCD4 (per 100,000)
Age group (years)
2001
2010
2019

Appendix Figure 27. Comparison of change in NCD4 mortality between 30 and 70 years of age from 2010 to 2019 with change from 2001 to 2010.

Each point represents one country, coloured by region. Data are shown for 185 countries in eight regions. The dotted diagonal line represents equal absolute change in probability of death from 2001 to 2010 and from 2010 to 2019. Segments of the plot are labelled to indicate whether countries experienced a change in the direction or magnitude of change in the probability of death from one decade to the next.

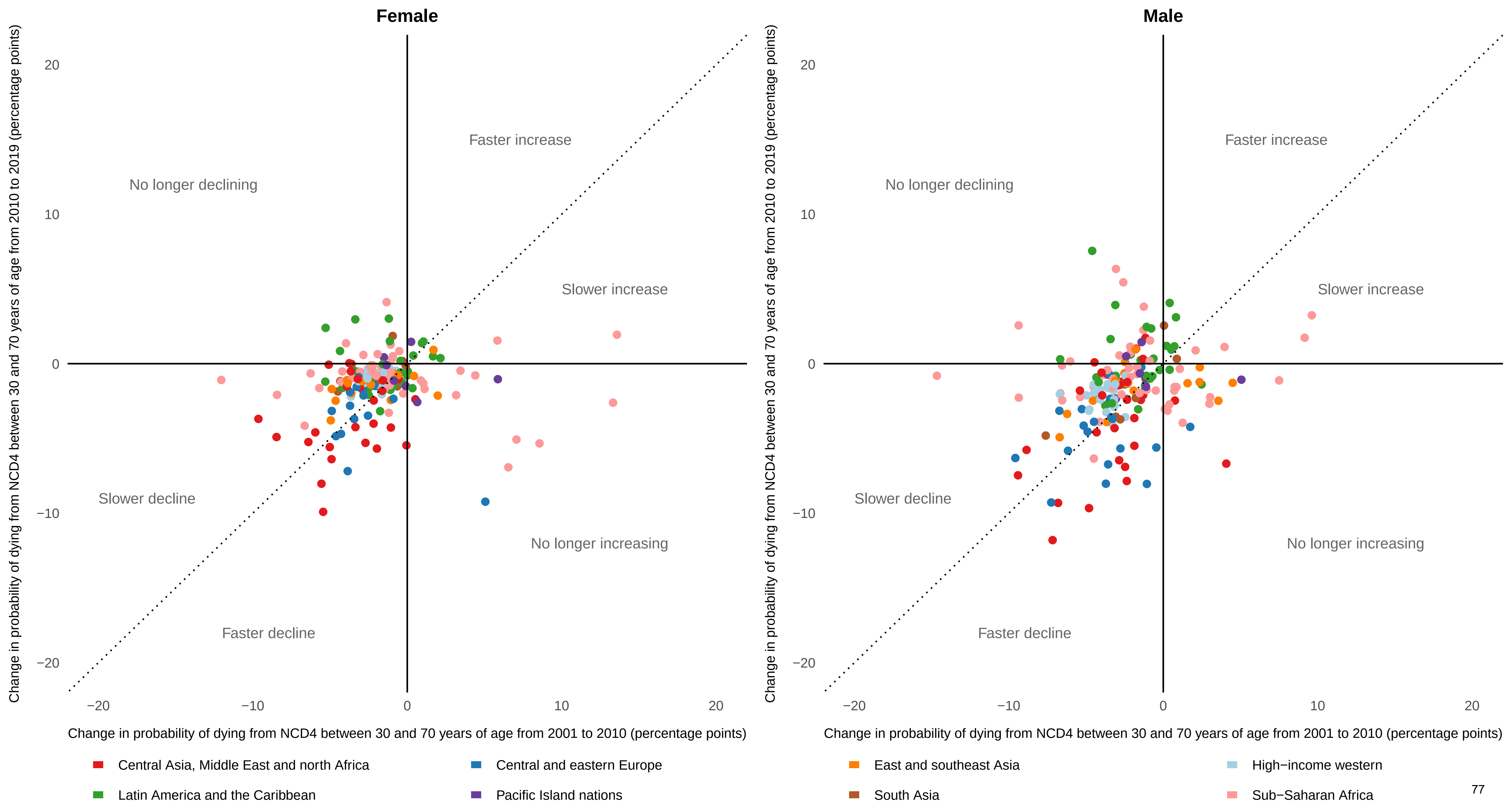

Female
Male
Faster increase
No longer declining
Slower increase
Slower decline
No longer increasing
Faster decline
Change in probability of dying from NCD4 between 30 and 70 years of age from 2010 to 2019 (percentage points)
Change in probability of dying from NCD4 between 30 and 70 years of age from 2001 to 2010 (percentage points)
20
10
0
−10
−20
Central Asia, Middle East and north Africa
Central and eastern Europe
East and southeast Asia
High−income western
Latin America and the Caribbean
Pacific Island nations
South Asia
Sub−Saharan Africa

References

1. Wadhera RK, Shen C, Gondi S, Chen S, Kazi DS, Yeh RW. Cardiovascular deaths during the COVID-19 pandemic in the United States. *JACC* 2021; **77**(2): 159-69.
2. British Heart Foundation (BHF). Excess deaths involving CVD in England since the onset of the COVID-19 pandemic: an analysis and explainer, 2023.
3. Tallack C, Krelle H. What has happened to non-COVID mortality during the pandemic? London: The Health Foundation, 2021.
4. Venter ZS, Aunan K, Chowdhury S, Lelieveld J. COVID-19 lockdowns cause global air pollution declines. *Proc Natl Acad Sci USA* 2020; **117**(32): 18984-90.
5. Sohi I, Chrystoja BR, Rehm J, et al. Changes in alcohol use during the COVID-19 pandemic and previous pandemics: a systematic review. *Alcohol Clin Exp Res* 2022; **46**(4): 498-513.
6. GBD 2021 Causes of Death Collaborators. Global burden of 288 causes of death and life expectancy decomposition in 204 countries and territories and 811 subnational locations, 1990-2021: a systematic analysis for the Global Burden of Disease Study 2021. *Lancet* 2024; **403**(10440): 2100-32.
7. World Health Organization (WHO). World health statistics 2024: monitoring health for the SDGs, Sustainable Development Goals. Geneva: World Health Organization, 2024.
8. NCD Countdown Collaborators. NCD Countdown 2030: worldwide trends in non-communicable disease mortality and progress towards Sustainable Development Goal target 3.4. *Lancet* 2018; **392**(10152): 1072-88.
9. Ndumele CE, Rangaswami J, Chow SL, et al. Cardiovascular-kidney-metabolic health: a presidential advisory from the American Heart Association. *Circulation* 2023; **148**(20): 1606-35.
10. Cheng G, Huang C, Deng H, Wang H. Diabetes as a risk factor for dementia and mild cognitive impairment: a meta-analysis of longitudinal studies. *Intern Med J* 2012; **42**(5): 484-91.
11. Kennelly SP, Lawlor BA, Kenny RA. Blood pressure and dementia - a comprehensive review. *Ther Adv Neurol Disord* 2009; **2**(4): 241-60.
12. Preston S, Elo I. Effects of age misreporting on mortality estimates at older ages. *Popul Stud* 1999; **53**: 165-77.
13. D'Amico M, Agozzino E, Biagino A, Simonetti A, Marinelli P. Ill-defined and multiple causes on death certificates--a study of misclassification in mortality statistics. *Eur J Epidemiol* 1999; **15**(2): 141-8.
14. Mieno MN, Tanaka N, Arai T, et al. Accuracy of death certificates and assessment of factors for misclassification of underlying cause of death. *J Epidemiol* 2016; **26**(4): 191-8.
15. Alpérovitch A, Bertrand M, Jougla E, et al. Do we really know the cause of death of the very old? Comparison between official mortality statistics and cohort study classification. *Eur J Epidemiol* 2009; **24**: 669-75.
16. Naghavi M, Makela S, Foreman K, O'Brien J, Pourmalek F, Lozano R. Algorithms for enhancing public health utility of national causes-of-death data. *Popul Health Metr* 2010; **8**: 9.
17. Global Health Estimates 2021: deaths by cause, age, sex, by country and by region, 2000-2021. Geneva: World Health Organization, 2024.
18. NCD Risk Factor Collaboration (NCD-RisC). Worldwide trends in underweight and obesity from 1990 to 2022: a pooled analysis of 3663 population-representative studies with 222 million children, adolescents, and adults. *Lancet* 2024; **403**(10431): 1027-50.
19. NCD Risk Factor Collaboration (NCD-RisC). Worldwide trends in diabetes prevalence and treatment from 1990 to 2022: a pooled analysis of 1108 population-representative studies with 141 million participants. *Lancet* 2024; **404**(10467): 2077-93.
20. World Health Organization (WHO). WHO methods and data sources for country-level causes of death 2000-2021. Geneva: World Health Organization, 2024.

21. Preston SH, Heuveline P, Guillot M. Demography: Measuring and Modeling Population Processes. Oxford: Blackwell Publishing; 2001.
22. Horiuchi S, Wilmoth JR, Pletcher SD. A decomposition method based on a model of continuous change. *Demography* 2008; **45**(4): 785-801.
23. Riffe T. DemoDecomp: Decompose Demographic Functions. CRAN, 2024.
24. NCD Countdown Collaborators. NCD Countdown 2030: pathways to achieving Sustainable Development Goal target 3.4. *Lancet* 2020; **396**(10255): 918-34.
25. GBD 2021 Europe Life Expectancy Collaborators. Changing life expectancy in European countries 1990-2021: a subanalysis of causes and risk factors from the Global Burden of Disease Study 2021. *Lancet Public Health* 2025; **10**(3): e172-e88.
26. Lopez AD, Adair T. Is the long-term decline in cardiovascular-disease mortality in high-income countries over? Evidence from national vital statistics. *Int J Epidemiol* 2019; **48**(6): 1815-23.
27. Islami F, Siegel RL, Jemal A. The changing landscape of cancer in the USA — opportunities for advancing prevention and treatment. *Nat Rev Clin Oncol* 2020; **17**(10): 631-49.
28. Shelton J, Zotow E, Smith L, et al. 25 year trends in cancer incidence and mortality among adults aged 35-69 years in the UK, 1993-2018: retrospective secondary analysis. *BMJ* 2024; **384**: e076962.
29. International Agency for Research on Cancer (IARC). Cancer research for cancer prevention. Lyon, France: GLOBOCAN, 2020.
30. Sung H, Jiang C, Bandi P, et al. Differences in cancer rates among adults born between 1920 and 1990 in the USA: an analysis of population-based cancer registry data. *Lancet Public Health* 2024; **9**(8): e583-e93.
31. Santucci C, Mignozzi S, Levi F, et al. European cancer mortality predictions for the year 2025 with focus on breast cancer. *Ann Oncol* 2025; **36**(4): 460-8.
32. Vaccarella S, Lortet-Tieulent J, Plummer M, Franceschi S, Bray F. Worldwide trends in cervical cancer incidence: impact of screening against changes in disease risk factors. *Eur J Cancer* 2013; **49**(15): 3262-73.
33. Vaccarella S, Li M, Bray F, et al. Prostate cancer incidence and mortality in Europe and implications for screening activities: population based study. *BMJ* 2024; **386**: e077738.
34. Lee YT, Wang JJ, Luu M, et al. The mortality and overall survival trends of primary liver cancer in the United States. *J Natl Cancer Inst* 2021; **113**(11): 1531-41.
35. Wojtyla C, Bertuccio P, Wojtyla A, La Vecchia C. European trends in breast cancer mortality, 1980-2017 and predictions to 2025. *Eur J Cancer* 2021; **152**: 4-17.
36. Jani C, Salciccioli I, Rupal A, et al. Trends in breast cancer mortality between 2001 and 2017: an observational study in the European Union and the United Kingdom. *JCO Glob Oncol* 2021; (7): 1682-93.
37. Kratzer TB, Bandi P, Freedman ND, et al. Lung cancer statistics, 2023. *Cancer* 2024; **130**(8): 1330-48.
38. Campbell C. Is dementia really on the rise? *The Actuary* 2017.
39. Wu YT, Beiser AS, Breteler MMB, et al. The changing prevalence and incidence of dementia over time - current evidence. *Nat Rev Neurol* 2017; **13**(6): 327-39.
40. Wolters FJ, Chibnik LB, Waziry R, et al. Twenty-seven-year time trends in dementia incidence in Europe and the United States: The Alzheimer Cohorts Consortium. *Neurology* 2020; **95**(5): e519-e31.
41. Stallard PJE, Ukraintseva SV, Doraiswamy PM. Changing story of the dementia epidemic. *JAMA* 2025.
42. Matthews FE, Arthur A, Barnes LE, et al. A two-decade comparison of prevalence of dementia in individuals aged 65 years and older from three geographical areas of England: results of the Cognitive Function and Ageing Study I and II. *Lancet* 2013; **382**(9902): 1405-12.

43. Ahmad S, Carey IM, Harris T, Cook DG, DeWilde S, Strachan DP. The rising tide of dementia deaths: triangulation of data from three routine data sources using the Clinical Practice Research Datalink. *BMC Geriatr* 2021; **21**(1): 375.
44. World Health Organization (WHO). Optimizing brain health across the life course: WHO position paper. Geneva: World Health Organization, 2022.
45. Khatibzadeh S, Farzadfar F, Oliver J, Ezzati M, Moran A. Worldwide risk factors for heart failure: a systematic review and pooled analysis. *Int J Cardiol* 2013; **168**(2): 1186-94.
